# SPICEiST: subcellular RNA pattern enhances cell clustering of imaging-based spatial transcriptomics

**DOI:** 10.1186/s44342-025-00056-1

**Published:** 2025-12-02

**Authors:** Sungwoo Bae, Yuchang Seong, Dongjoo Lee, Hongyoon Choi

**Affiliations:** 1 Portrai, Inc., 57, Seongsui-ro 22-gil, Seongdong-gu, Seoul, 04798 Republic of Korea; 2 Department of Nuclear Medicine, Seoul National University Hospital, 101, Daehak-ro, Jongno-gu, Seoul, 03080 Republic of Korea

**Keywords:** Imaging-based spatial transcriptomics, Gene panel, Graph autoencoder, Subcellular gene expression, Clustering, Tumor microenvironment

## Abstract

**Background:**

Imaging-based spatial transcriptomics (ST) enables the quantification of gene expression at single-cell resolution while preserving spatial context, but its utility is limited by small gene panels and challenges in accurate cell segmentation. To address these limitations, we present a graph autoencoder framework that integrates subcellular transcript distribution patterns with cell-level gene expression profiles for enhanced cell clustering in imaging-based ST (SPICEiST).

**Results:**

The clustering performance of SPICEiST was systematically evaluated across several cancer datasets and gene panel sizes. The results demonstrate that SPICEiST consistently outperforms the conventional cell-level gene expression-based methods in distinguishing subtle differences in cell states, as measured by the number of cell clusters and clustering indices, such as the CHI and DBI. Moreover, the findings indicate that SPICEiST can further enhance the performance, even with advancements in cell segmentation, particularly for datasets with small gene panels. Overall, these improvements in cell clustering indices, CHI and DBI, were more pronounced in datasets with small gene panels of around 300 genes, in contrast to those with large panels containing over a thousand genes. Notably, SPICEiST also reveals more spatially intermixed and less compartmentalized cell clusters, a characteristic that better reflects the complex and heterogeneous nature of tumor microenvironments. This effect was especially evident in the datasets with large panels.

**Conclusions:**

These findings highlight the value of leveraging subcellular transcript patterns to overcome the inherent limitations of imaging-based ST, particularly for small gene panels, and may provide new insights into tumor heterogeneity.

**Supplementary Information:**

The online version contains supplementary material available at 10.1186/s44342-025-00056-1.

## Background

Spatially resolved transcriptomics (ST) provides a deeper understanding of the spatial context of biological phenomena within the tissue at single-cell resolution [1, 2]. One notable technology, imaging-based ST, captures the locations of predefined RNA types and delineates cell boundaries using immunofluorescence images [1]. This enables the quantification of transcript abundance at the single-cell level while maintaining the spatial location of the cells. Imaging-based ST, such as Xenium, MERSCOPE, and CosMx SMI, achieve high efficiency in capturing transcripts and can obtain subcellular-scale transcript distributions [3–5]. However, the types of genes that can be targeted simultaneously, referred to as a “plex,” typically ranges from a few hundred to a few thousand. This limitation makes imaging-based ST less suitable for unbiased screening of the tissue microenvironment [6].

In most studies, imaging-based ST is analyzed similarly to single-cell RNA sequencing (scRNA-seq) datasets, starting with a cell-by-gene transcript count matrix [4]. However, imaging-based ST typically encompasses a significantly smaller number of genes compared to scRNA-seq, which captures transcriptome-wide expression profiles. Furthermore, in imaging-based ST, transcripts are allocated to cells by analyzing the images of cell membranes or the expansion of nuclear regions, instead of applying cell dissociation methods [4]. This technique can result in inaccuracies when assigning transcripts to cells, mainly because of the substantial overlap between closely situated cells in a 2D space. Consequently, in imaging-based ST, the combination of a limited plex and potential errors in cell segmentation leads to ambiguous distinctions between cell types based on unsupervised cell clustering [7].

Numerous strategies have been explored to address the challenges of limited plex and cell segmentation, aiming to enhance the clustering of cell types in imaging-based ST. Recent innovations in multiplexed imaging technologies have significantly expanded the gene panel in imaging-based ST, with some methods capable of analyzing up to approximately 5000 genes [8]. Additionally, other approaches utilize computational techniques to impute missing genes, thereby broadening the transcriptomic profile captured by imaging-based ST [9–12]. To address cell segmentation issues, newly introduced transcript density-based approaches provide more accurate delineation of cell boundaries [7]. Furthermore, one of the methods estimate the distribution of transcripts that are intermixed from adjacent cells and backgrounds, allowing for corrections in the cell-by-gene count matrix [13]. In addition to gene panels and cell segmentation, incorporating the spatial coordinates of each cell alongside gene expression can enhance cell clustering performance, highlighting the importance of the location of a cell within the tissue for accurate characterization [14, 15].

Another approach to enhancing cell clustering using imaging-based ST is examining subcellular transcript distribution, which is anticipated to yield additional insights beyond those obtained from cell-level gene expression. Subcellular transcript distribution can vary within the same cell type, depending on the state of the cell, including transcriptional processes and regulations [16, 17]. Previous studies have leveraged these subcellular patterns and RNA colocalization to finely characterize subcellular domains and their functional implications [18, 19]. Besides, one study enhanced the processes of cell segmentation and annotation in ST by employing a multi-scale topology-based approach [20].

However, the subcellular transcript patterns remain largely unexplored when it comes to enhancing cell clustering in imaging-based ST. The primary challenge lies in effectively capturing these patterns, which are distributed across cells that vary in size, shape, and transcript abundance. Graph-based models are well-suited for this task because they can naturally represent irregular spatial data with high flexibility [21]. Therefore, there is an unmet need for a method that uses a graph-based model to integrate these subcellular features with conventional cell-level expression profiles.

The objective of this study is to utilize a graph autoencoder for imaging-based ST of tumor tissues to extract subcellular gene expression patterns and integrate individual cellular gene expression profiles. By leveraging this approach, named SPICEiST (*S*ubcellular *P*attern *I*ntegration with *C*ellular *E*xpression in *I*maging-based *ST*), to extract subcellular patterns, it is expected to overcome the limitations associated with imaging-based ST, thereby facilitating the identification of biologically significant yet subtle variations among cells. Furthermore, this study will quantitatively assess the enhancements in clustering performance within tumors according to the gene plex and the granularity of the cell type clustering.

## Methods

### Composition of the publicly available datasets

#### Xenium human lung cancer: version 1 and prime 5 K

The Xenium dataset was derived from formalin-fixed paraffin-embedded (FFPE) tissues of a lung adenocarcinoma patient (https://www.10xgenomics.com/datasets/xenium-human-lung-cancer-post-xenium-technote). The tissue slide underwent analysis using two distinct platforms: version 1 (v1) and Prime 5 K. This approach allowed for a direct comparison of the panel sizes on the same tissue slide. The total number of detected transcripts reached 32,073,729 for v1 and 177,464,221 for 5 K. The types of transcripts identified, the gene panel size, were 289 and 5001, respectively. Additionally, the number of cells with a total count exceeding 10 was 268,072 for v1 and 275,556 for 5 K. Each slide was split into a 4 × 4 patches, with each individual patch serving as a separate dataset, and the patch with no assigned transcript in the cell was excluded from the downstream analysis.

#### Xenium human colorectal cancer

The Xenium dataset was derived from FFPE tissues of a colon adenocarcinoma patient (https://www.10xgenomics.com/datasets/human-colon-preview-data-xenium-human-colon-gene-expression-panel-1-standard). This dataset encompasses a total of 32,073,729 transcripts across 325 unique transcript types. Additionally, the number of cells with a total count exceeding 10 was 630,998. Each slide was split into a 4 × 4 patches, with each individual patch serving as a separate dataset, and the patch with no assigned transcript in the cell was excluded from the downstream analysis.

#### CosMx SMI—lung adenocarcinoma

The CosMx SMI dataset was created from a FFPE tissue slide sourced from primary tumors of a treatment-naive lung adenocarcinoma patients (Lung 5–1) [20, 22]. The tissue slide contains a total of 37,226,610 transcripts, representing 960 unique transcript types. The number of cells with a total count exceeding 10 was 99,181. Each slide was split into a 3 × 3 patches, with each individual patch serving as a separate dataset, and the patch with no assigned transcript in the cell was removed from the downstream analysis.

#### Single-cell RNA-sequencing datasets—Lung and colon adenocarcinoma

Publicly available single-cell RNA sequencing (RNA-seq) datasets were utilized to annotate cells in imaging-based spatial transcriptomics (ST). The lung adenocarcinoma (LUAD) tissues from untreated primary lung tumors and the colon adenocarcinoma (COAD) tissues from untreated primary colon tumors were selected for analysis [23, 24]. For LUAD and COAD, respectively, we used 45,149 cells with 29,634 genes and 21,657 cells with 22,276 genes for downstream analysis. We annotated the cell types using information from the original authors and the 3CA database [25].

### Dataset preparation

The whole slide of imaging-based ST was segmented into a NxN patches, each containing an average of over 10,000 cells. This enabled a comprehensive comparison of the gene expression-based cell clustering method (GEX) and the enhanced approach (SPICEiST) in a large number of cases. The cases in which there was no assigned transcript to cells were excluded from the downstream analysis. Additionally, this analysis included a comparison between v1 and the 5 K Xenium dataset obtained from the same tissue, which has 289 and 5001 genes in the panel, respectively. The cells that had a total count exceeding 10 were filtered for the subsequent analysis. To annotate the cell types, the TACCO method was employed to transfer the cell labels from the single-cell RNA-seq to the imaging-based ST datasets [26].

### Building cell graphs and constructing graph autoencoders

**Fig. 1:**
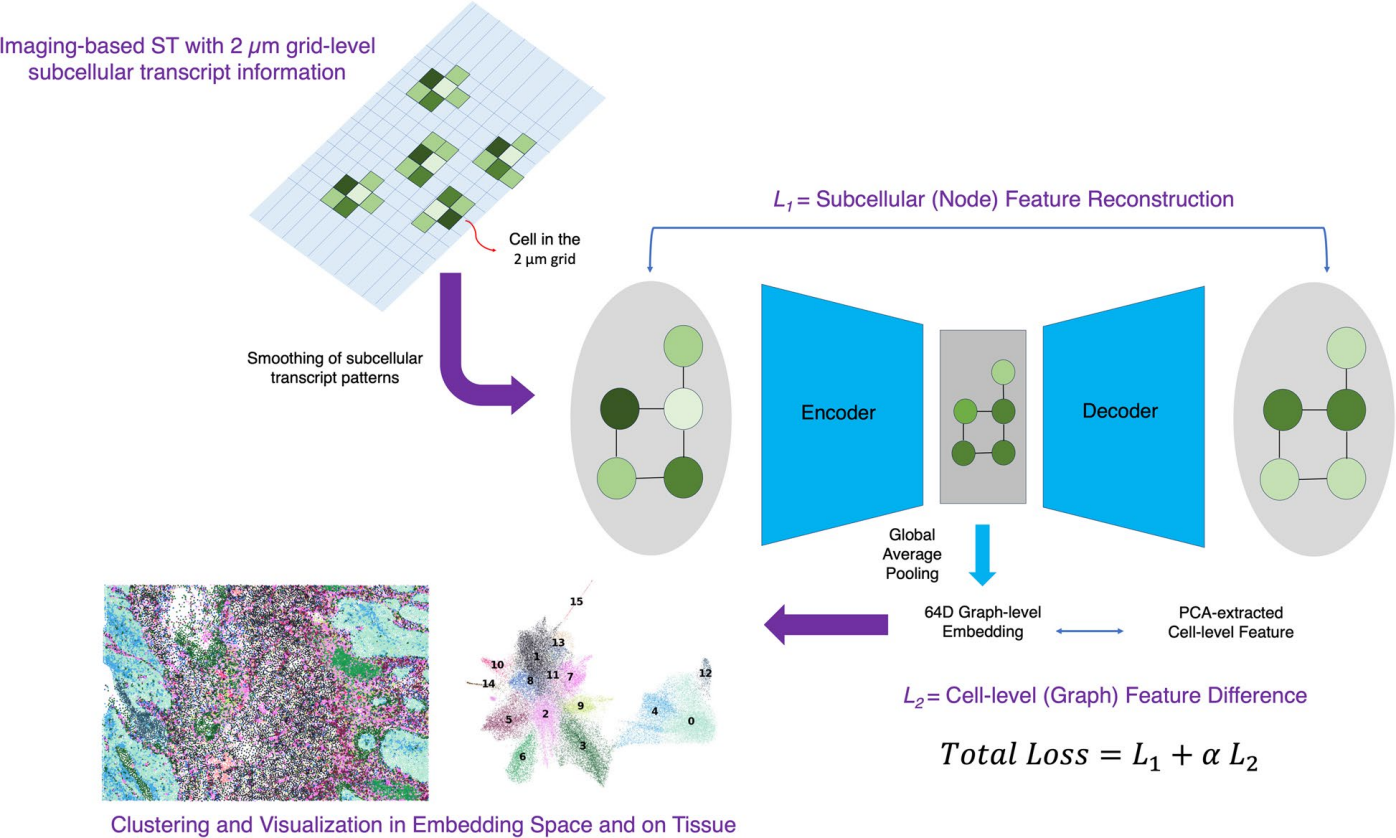
Schematic image for SPICEiST. To address the limitations of imaging-based ST, subcellular patterns of gene expression, along with cell-level gene expression, were utilized to extract core features that most accurately represent cellular states. For preprocessing, Gaussian smoothing was applied to the 2 µm subcellular-level count matrix, followed by L_2_ normalization. Graphs were constructed for each cell by designating the 2 µm grid as nodes, the 1-hop neighbor grid as connected edges, and the node features as subcellular-level gene expression. A graph autoencoder consists of an encoder, built with graph convolutional blocks, and a decoder for node feature reconstruction. Its training is guided by a weighted, dual-objective loss function. This function simultaneously minimizes the reconstruction error of subcellular features while enforcing similarity between the 64D latent embedding of the model and the cellular expression profile. Finally, cell clustering was performed using the integrated features, applying the Louvain algorithm to identify distinct cell clusters

The transcript coordinates for M types of genes (where M denotes the total number of genes in the panel) from the imaging-based ST dataset were utilized to assign each transcript to a distinct grid location with a designated grid size (formula 1). Subsequently, the cells were segmented using the vendor-provided method (10x) [27], which is based on the multimodal segmentation of images of the cell nucleus, cell boundary, and cell interior. Then, transcripts along with grids were allocated to each cell. The grid count matrix underwent smoothing with a truncated and normalized Gaussian kernel for each cell, utilizing a sigma (σ) value of 1.5 times the unit grid size (formula 2). Then, L_2_ normalization was applied as an input to the neural network (Fig. 1) (formula 3). The count matrix of individual cells was normalized to a total sum of 100, log-transformed (log1p), and then scaled with a mean of 0 and a standard deviation of 1 (formula 4, 5, 6) [4]. Principal component analysis was performed to extract the top 64 components that explain the variance of cell-level gene expression the most (formula 6).

For cell c, grid location $g\in {Grid}_{c}$ and gene $m\in \{1, 2, \dots ,M\}$,

1 $${x}_{c,g,m}=\sum_{t\in {T}_{c,m}}1 \left\{coord\left(t\right)\in grid\left(g\right)\right\}$$

2 $${\widetilde x}_{c,i,m}=\frac{\sum_{j\in N_\sigma(i)}e^{(-\frac{{\Arrowvert\overset\rightharpoonup{r_i}-\overset\rightharpoonup{r_j}\Arrowvert}^2}{2\sigma^2})}x_{c,j,m}}{\sum_{j\in N_\sigma(i)}e^{(-\frac{{\Arrowvert\overset\rightharpoonup{r_i}-\overset\rightharpoonup{r_j}\Arrowvert}^2}{2\sigma^2})}},N_\sigma\left(i\right)=\{j:\Arrowvert\overset\rightharpoonup{r_i}-\overset\rightharpoonup{r_j}\Arrowvert\leq3\sigma\}$$

3 $${\widehat{x}}_{c,g,m}=\frac{{\widetilde{x}}_{c,g,m}}{\sqrt{{\sum }_{g,m}{\widetilde{x}}_{c,g,m}^{2}}}$$

4 $${u}_{c,m}=\sum\limits _{g\in {Grid}_{c}}{x}_{c,g,m}$$

5 $${u}_{c,m}^{log}= \mathit{ln}\left(1+100\frac{{u}_{c,m}}{{\sum }_{{m}{\prime}=1}^{M}{u}_{c,{m}{\prime}}}\right)$$

6 $${z}_{c}=PC{A}_{64}\left(\frac{{{u}_{c,m}^{log}-\mu }_{m}}{{\sigma }_{m}}\right)\in {\mathbb{R}}^{64}$$

For each cell, a graph was defined that connects the four neighboring grids (up, down, right, left) with edges (formula 7). Node features were assigned in two layers: the first layer represented grid-level subcellular gene expression values, while the second layer represented cell-level gene expression values. This approach effectively transforms the transcripts within the cell into a graph that integrates both subcellular and cell-level gene expression profiles (Fig. 1).

7 $$G_c=\left(V_c,E_c\right),V_c={Grid}_c,E_c=\{\left(g,g'\right):g'\in NN(g)\}\},NN:\mathrm{nearest}\;\mathrm{neighbors}$$

The graph autoencoder was developed with a structure that includes an encoder and a decoder. The encoder consists of two convolutional blocks, each utilizing a graph convolutional neural network (GCN) [28], followed by batch normalization (BN), rectified linear unit (ReLU) activation, and dropout layers (Drop) set at a probability of 0.2. This process results in a latent feature representation with dimensions of 128 and 64 after each convolutional block (formula 8 and 9). In contrast, the decoder is structured with a fully connected layer (FC), batch normalization (BN), ReLU activation, a dropout layer (Drop), and concludes with another FC layer as the final output (formula 10) (Fig. 1).

8 $${H}_{c}^{\left(1\right)}\left(g\right)=Drop\left(ReLU\left(BN\left(GC{N}_{128}({\widehat{x}}_{c,g,1:M}, {E}_{c})\right)\right)\right)$$

9 $${H}_{c}^{\left(2\right)}\left(g\right)=Drop\left(ReLU\left(BN\left(GC{N}_{64}\left({H}_{c}^{\left(1\right)}\left(g\right), {E}_{c}\right)\right)\right)\right)$$

10 $${\widehat{X}}_{c,g,1:M}=FC\left(Drop\left(ReLU\left(BN\left(FC\left({H}_{c}^{2}\left(g\right)\right)\right)\right)\right)\right)$$

### Training the graph autoencoder

The loss function is divided into two components. The first component (*L*_*1*_) calculates the mean squared error (MSE) loss for the reconstruction of subcellular node features that have passed through both the encoder and decoder, compared to the original subcellular features (formula 11). The second component (*L*_*2*_) defines the MSE loss between the averaged node features across the nodes, with the final feature number of 64 (formula 12), and the PCA-derived cell-level gene expression feature (formula 13). The weight term, alpha (α), was applied to the second loss component and then combined with the first loss (formula 14). A higher value of alpha signifies that the cell-level expression profiles exert a greater influence on the training loss (Fig. 1). Conversely, a lower alpha indicates that the subcellular expression profiles have a more pronounced effect on the training loss. The optimization process employs the Adam optimizer, with the learning rate configured at 10^–3^.

11 $${L}_{1}=\frac{1}{C}{\sum }_{c}\frac{1}{{|V}_{c}|M}{\sum }_{g\in {V}_{c}}\sum_{m=1}^{M}{\left({\widehat{X}}_{c,g,m}-{\widehat{x}}_{c,g,m}\right)}^{2}$$

12 $${\overline{h} }_{c}=\frac{1}{{|V}_{c}|}{\sum }_{g\in {V}_{c}}{H}_{c}^{\left(2\right)}(g)\in {\mathbb{R}}^{64}$$

13 $${L}_{2}=\frac{1}{C}{\sum }_{c}{\Vert {\overline{h} }_{c}-{z}_{c}\Vert }_{2}^{2}$$

14 $$L={L}_{1}+\alpha {L}_{2}$$

The datasets were partitioned into training and validation sets at an 80:20 ratio and trained for 50 epochs. During each epoch, training involved randomly selecting 32 batches and computing the average loss. After each epoch, the trained model was utilized to assess validation loss by selecting 32 batches without shuffling, followed by the calculation of the average loss for that epoch. An early stopping strategy was employed, enabling the training process to persist until the validation loss failed to decrease by more than 10^–4^ over three consecutive iterations compared to the lowest loss observed. The model with the lowest validation loss was selected.

### Assessment of cell clustering performance

To perform cell clustering using the integrated features of cell and grid-level subcellular expression, we utilized a trained model to calculate a 64D latent representation of the graph structure for each cell (Fig. 1). This latent feature was subsequently utilized to compute the 16-nearest neighbor graphs, after which the Louvain algorithm was applied to cluster the cells into distinct cell groups. The resolution of these clusters was varied from 0.3 to 1.8 (specifically at 0.3, 0.6, 0.9, 1.2, 1.5, and 1.8) to adjust the granularity of the clusters. We repeated this process for the cell-level gene expression features (referred to as GEX), which were represented by 64 principal components (PCs), and defined the cell clusters in a similar manner.

The clustering performance was evaluated using three primary indices: the Average Silhouette Width (ASW), the Calinski-Harabasz index (CHI), and the Davies-Bouldin index (DBI). They assess how closely the cells within the same clusters are grouped together and how well the cells in different clusters are separated in the feature space [29–32]. ASW and CHI are indices where higher values indicate better performance, while DBI is an index where lower values signify superior clustering effectiveness. These indices were computed based on the latent representations (e.g., from 64D embedding of SPICEiST), utilizing cell cluster labels generated by the Louvain algorithm. This approach serves as a performance measure, employing embeddings to evaluate unsupervised clustering.

Furthermore, to assess the spatial coherence of the cell clustering results, we computed the node assortativity coefficient based on a spatial-proximity network considering the 16 nearest neighbors as connected. In this framework, a high coefficient demonstrates strong spatial assortativity, meaning that data points belonging to the same cell clusters are significantly more likely to be spatial neighbors than would be expected by chance [33]. In other words, a lower coefficient indicates less organized cell clusters within the tissue. This provides a single, robust measure of how well the identified clusters are segregated into spatially compact regions.

In addition to unsupervised metrics, the performance of the clustering was evaluated using homogeneity and completeness scores. This was done by comparing the Louvain cluster labels to the ground-truth cell type annotations from TACCO algorithm. The homogeneity score measures cluster purity. A high homogeneity score indicates that each cluster consists of cells from only one ground-truth cell type. In contrast, the completeness score measures group integrity. A high completeness score indicates that all cells of a specific ground-truth cell type are grouped together within a single cluster. Often, there is a trade-off between these two metrics. A combination of high homogeneity and low completeness is particularly insightful because it signifies that a single ground-truth cell type has been split into multiple Louvain clusters. This pattern, known as over-clustering, is often desirable from a biological standpoint because it suggests that the cell clustering method has successfully identified meaningful cell subtypes within a broader known cell population.

Clustering performance was evaluated across two distinct imaging-based platforms, Xenium and CosMx SMI, with a focus on two types of cancer: lung and colon adenocarcinoma. Initially, the paired Xenium dataset for human lung cancer, derived from the v1 and 5 K platforms, was utilized. This dataset comprises 289 and 5001 genes, respectively, and was employed to assess the enhancement of clustering performance through subcellular gene expression patterns, while also comparing the effects based on different panel sizes. Next, datasets from Xenium for human colon cancer and CosMx for human lung cancer were analyzed to evaluate the scalability of the method in improving clustering outcomes.

### Comparison of the cell clustering performance

The performance of cell clustering was evaluated by comparing three distinct approaches. The first was a non-spatial baseline method (GEX), which treated imaging-based ST data as a single-cell RNA-seq dataset. This method relied solely on cell-level gene expression profiles. The second approach was SpaceFlow, a spatial clustering method that incorporates the spatial coordinates of cells alongside their gene expression. SpaceFlow was selected based on a benchmark study that demonstrated its high performance with imaging-based ST data [14, 34]. The third method was our proposed SPICEiST, which integrates subcellular and cellular-level expression information.

Furthermore, we sought to confirm whether applying SPICEiST provides additional benefits beyond those of a more accurate cell segmentation method alone. To test this theory, we repeated the comparison using Proseg, a state-of-the-art method which has demonstrated superior performance in terms of cell segmentation in imaging-based ST [7]. This enabled us to isolate the impact of subcellular transcript distribution from that of the cell segmentation algorithm itself.

## Results

A graph autoencoder was constructed that represents each cell as a spatial graph of its subcellular expression patterns. In these graphs, nodes correspond to unit grids and are featured with both local (subcellular-level) and global (cell-level) gene expression data. Using graph convolutional layers, the autoencoder learns a 64D latent embedding for each cell. Training is guided by a dual-objective loss function that reconstructs fine subcellular details while ensuring the latent space reflects the overall cellular expression profile. A hyperparameter, alpha, balances these two objectives (Fig. 1).

### Parameter optimization for SPICEiST: loss weight and grid size

The parameter optimization was performed in the randomly selected six patches from a total of 15 patches for both Xenium v1 and Prime 5 K datasets. A comparative analysis of the clustering indices ASW, CHI, and DBI was conducted in relation to alterations in two parameters: loss weight (α) and grid size (Supplementary Fig. S1). When the loss weight increased from 0.25 to 2.0 (0.25, 0.5, 1.0, and 2.0), there was no significant difference in ASW, CHI, and DBI indexes. On the other hand, when the grid size increased from 1 to 4 µm (1.0, 1.5, 2.0, 2.5, 3.0, and 4.0 μm), the ASW and DBI showed different patterns of change. For the v1 dataset, the median of ASW and DBI peaked and troughed at a grid size of 2.0 within the range of 1.0 to 3.0, and the cluster resolution below 1.5. For the 5 K dataset, the ASW showed a similar trend, while the DBI showed no significant change. There were no significant changes in the CHI with grid size.

For the loss weight, since a smaller alpha value indicates greater importance in the training loss for subcellular gene expression, we set alpha to 0.25 for further analysis. For the grid size, 2 µm offers an optimal balance between performance and efficiency, considering the significant memory and processing costs.

### Comparison of performance according to cell segmentation and clustering methods in the Xenium lung cancer dataset

In addition to evaluating the performance of SPICEiST across its various parameters, we also compared its performance against other methods for improving cell clustering in imaging-based ST. The GEX method was used as a baseline for comparison with SpaceFlow [34], the suggested spatial clustering method in the benchmark study [14], and SPICEiST. Subsequently, comparisons were made, both in the vendor-provided cell segmentation method (10x) and another method, Proseg [7], to demonstrate that the incorporation of subcellular gene expression patterns results in a superior cell clustering, in addition to the simple improvement of cell segmentation. The Xenium lung cancer dataset, which had been used for parameter optimization previously, was employed again in this study. However, the remaining nine patches, excluding the previous six for the parameter optimization, were used for cell clustering performance comparison.

**Fig. 2:**
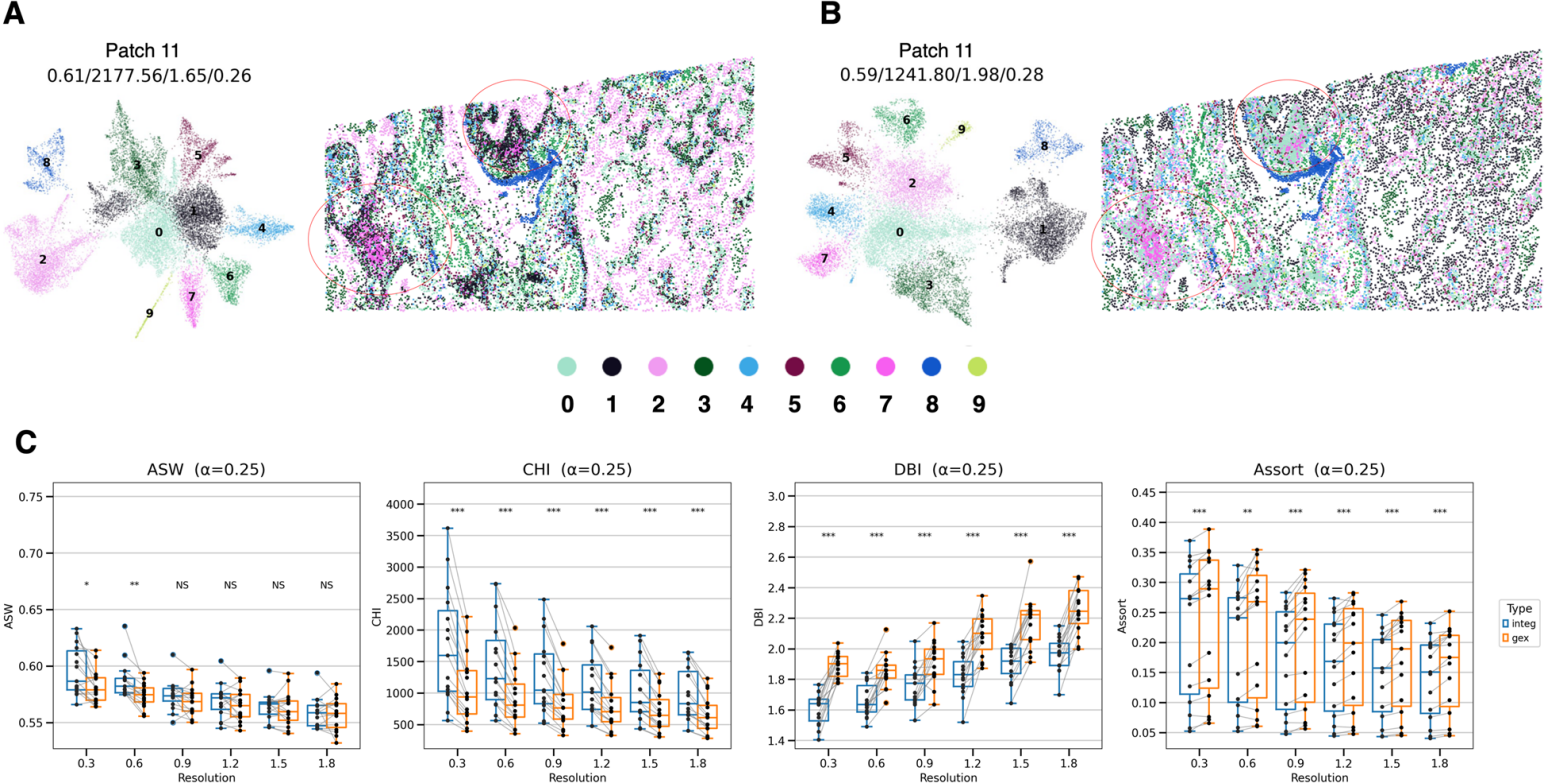
Comparison of the SPICEiST clustering results with those obtained from cell-level gene expression-based clustering in the Xenium human lung cancer dataset with v1 panel. The alpha weight for the loss was set to 0.25 during training. UMAP plots (left panel in each subplot) and the spatial distribution of cells (right panel in each subplot) illustrate the cell-level embedding and spatial distribution derived from (**A**) SPICEiST and (**B**) GEX-based clustering, respectively. The four numerical values at the top of the plot represent ASW, CHI, DBI, and assortativity coefficient. The tissue sample was obtained from patch number 11, and the cluster resolution was set to 0.3. Each dot in the plot represents cells, with the color indicating the identity of the clusters. **C** The boxplot compares four distinct clustering performance indices: ASW, CHI, DBI, and the assortativity coefficient, across varying resolutions of cell clusters. The notation X–Y represents the cell clustering method X and the cell segmentation method Y. For example, SPICEiST-10x indicates that the SPICEiST clustering method was applied with the 10x cell segmentation. The stars above the boxplots show the results of statistical comparisons (Wilcoxon signed-rank test) of GEX-10x with SpaceFlow-10x and SPICEiST-10x, and of GEX-proseg with SpaceFlow-proseg and SPICEiST-proseg. Multiple comparison correction was performed using the Benjamini–Hochberg method. *: *p* $\le$ 0.05, **: *p* $\le$ 0.01, ***: *p* $\le$ 0.001, ****: *p* $\le$ 0.0001

The clusters were visualized on a UMAP plot, and the clustering performance indices (ASW, CHI, and DBI) were compared between the SPICEiST, SpaceFlow, and the gene expression-based method (GEX) for a v1 gene panel. This analysis shows how clustering performance varies according to cell clustering method, as well as cell cluster granularity. Visualization of the cell clusters in the low-dimensional UMAP plot revealed that the SPICEiST provided a clearer distinction between the cell clusters than the GEX and SpaceFlow did at a cluster resolution of 0.3 with the 10x cell segmentation method (Fig. 2A, B and Supplementary Fig. S2A, C, E).

To quantitatively evaluate clustering performance across cell segmentation, clustering, and cell cluster granularity, we employed indices describing clustering patterns within and between clusters. Using the 10x cell segmentation method, CHI and DBI demonstrated consistently superior performance in SPICEiST compared to the SpaceFlow and GEX models across varying cell granularity, as indicated by Louvain cluster resolution (Fig. 2C). Besides, when Proseg was used for cell segmentation, SPICEiST showed a trend of superior performance compared to the SpaceFlow and GEX models, as represented by the median value. On the other hand, ASW exhibited improved clustering performance in SPICEiST compared to SpaceFlow using both cell segmentation methods at all cluster resolutions and in GEX at lower resolutions (Fig. 2C**)**.

The SPICEiST model exhibited improved performance on standard clustering indices. However, this improvement was accompanied by a compromise in spatial organization (Fig. 2A, B and Supplementary Fig. S2B, D, F). Notably, the assortativity coefficient was significantly lower in SPICEiST than in SpaceFlow and GEX, regardless of cell segmentation methods (Fig. 2C and Supplementary Fig. S2). While the SPICEiST generated distinct clusters in gene expression, it identified cell types that exhibited a more spatially dispersed and less compartmentalized arrangement compared to those identified by the SpaceFlow and GEX models. This finding may more accurately represent the disorganized and heterogeneous nature of the tumor than other models do. For instance, cell clusters from patch number 11, selected from a total of 15 patches, were mapped to the corresponding tissue and analyzed across both methods (Fig. 2C and Supplementary Fig. S2, 3). The visual examination of the spatial distribution of the clusters revealed that the SPICEiST exhibited a globally well-structured pattern. In contrast, it also displayed a more intricate local intermixture of cell clusters compared to the GEX model, highlighting the complexity of the cell organization (red-circled regions in Fig. 2C). This trend of differences was observed across multiple different patches, with the local intermixture of cells being more prominent in the SPICEiST (Supplementary Fig. S2B, D, F).

**Fig. 3:**
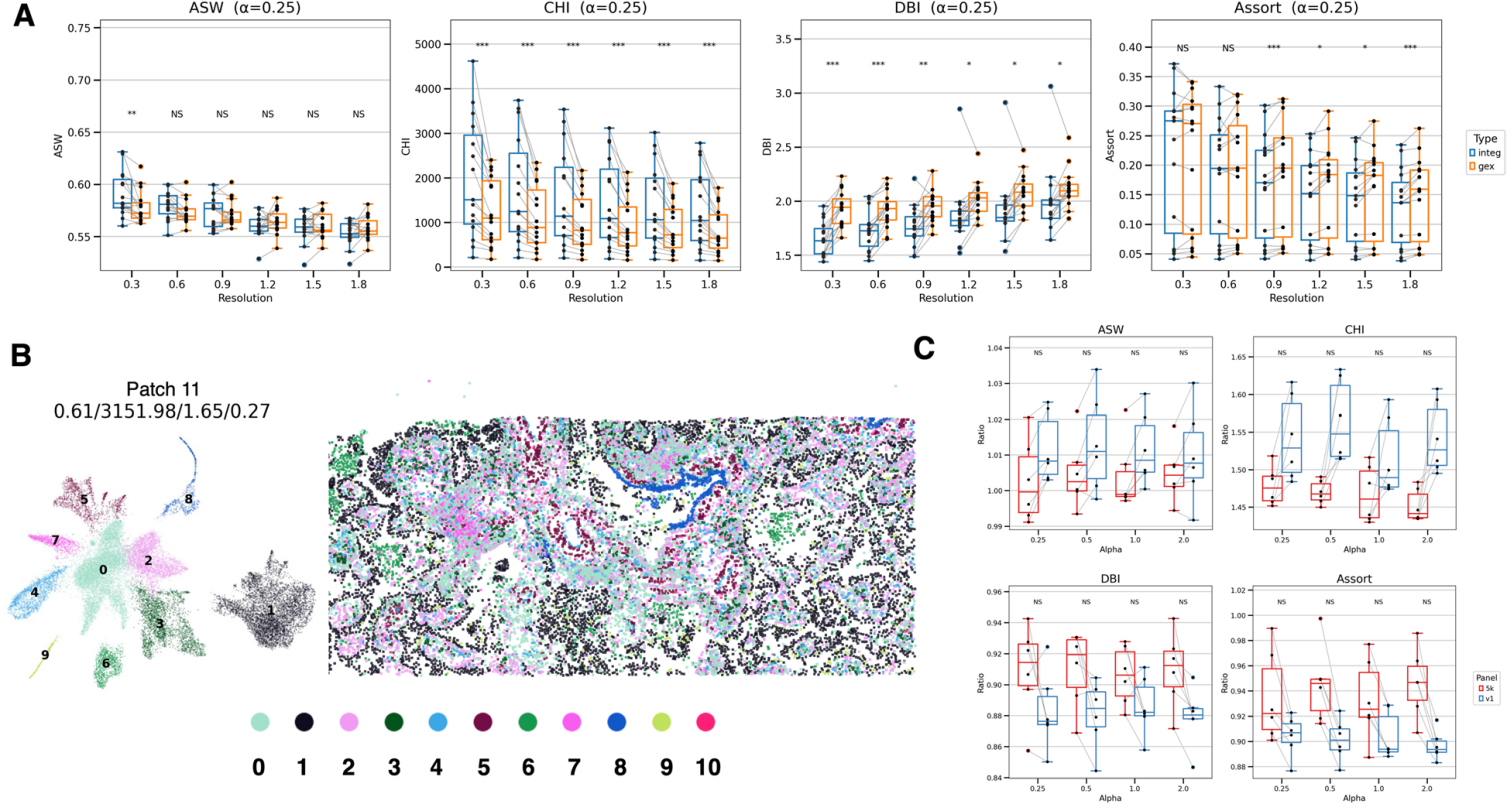
Comparison of the SPICEiST clustering results with those obtained from cell-level gene expression-based clustering in the Xenium human lung cancer dataset with 5 K panel. **A** The boxplot compares four distinct clustering performance indices: ASW, CHI, DBI, and the assortativity coefficient, across varying resolutions of cell clusters. The alpha weight for the loss was set to 0.25 during training. The notation X–Y represents the cell clustering method X and the cell segmentation method Y. For example, SPICEiST-10x indicates that the SPICEiST clustering method was applied with the 10x cell segmentation. The stars above the boxplots show statistical comparisons (Wilcoxon signed-rank test) of GEX-10 x with SpaceFlow-10x and SPICEiST-10x, and of GEX-proseg with SpaceFlow-proseg and SPICEiST-proseg. Multiple comparison correction was performed using the Benjamini–Hochberg method.. UMAP plots (left panel in each subplot) and the spatial distribution of cells (right panel in each subplot) illustrate the cell-level embedding and spatial distribution derived from (**B**) SPICEiST-based clustering. The tissue sample was obtained from patch number 11, and the cluster resolution was set to 0.3. The four numerical values at the top of the plot represent ASW, CHI, DBI, and assortativity coefficient. Each dot in the plot represents cells, with the color indicating the identity of the clusters. **C** The boxplot compares the log mean ratio of clustering performance indices between SPICEiST and other methods on the Xenium v1 and Prime 5 K platforms. The notation X–Y in the title represents the ratio calculated between methods X and Y (X/Y). The stars above the boxplots show the results of statistical comparisons (Wilcoxon signed-rank test) between Xenium v1 and 5 K. Multiple comparison correction was performed using the Benjamini–Hochberg method. NS: Not significant, *: *p* $\le$ 0.05, **: *p* $\le$ 0.01, ***: *p* $\le$ 0.001, ****: *p* $\le$ 0.0001

Then, the ASW, CHI, and DBI were compared for a 5 K gene panel between the SPICEiST and GEX models. This analysis shows the clustering performance according to cell segmentation methods with varying cluster granularities. Using the 10x cell segmentation method, CHI and DBI demonstrated consistently superior performance in SPICEiST compared to the SpaceFlow and GEX models across varying cluster resolutions (Fig. 3A). However, when Proseg was used for cell segmentation, SPICEiST showed a trend of superior performance compared to the GEX model, though it did not outperform the SpaceFlow, particularly for the CHI. Meanwhile, when using the 10x cell segmentation method, ASW exhibited enhanced clustering performance in SPICEiST compared to SpaceFlow and GEX at lower cluster resolution. When Proseg was used, however, SPICEiST showed a trend of lower performance compared to SpaceFlow and GEX (Fig. 3A).

Regarding assortativity coefficient, a significant decrease in the value was noted in the SPICEiST compared to the SpaceFlow and GEX models, irrespective of the cell segmentation method. However, this decrease was only observed in cases of high cell cluster resolution above 0.6 (Fig. 3A). The cell clusters from patch number 11, the same patch used in the v1 platform for visualization, were mapped to the corresponding tissue and analyzed across SPICEiST, SpaceFlow, and GEX methods for 10x segmentation method. The visual assessment of the spatial distribution of the clusters at a resolution of 0.3 revealed that the SPICEiST did not show significant differences in the global and local patterns of clusters across multiple patches, including the patch 11 (Fig. 3B and Supplementary Fig. S4).

In addition to clustering metrics and spatial indices, we compared the number of clusters generated by SPICEiST, SpaceFlow, and GEX models across different imaging-based ST platforms and cell segmentation methods (Supplementary Fig. S5). On the Xenium v1 platform, SPICEiST produced a significantly higher number of clusters than the other methods at resolutions above 0.9, regardless of the segmentation approach. In contrast, on the Xenium Prime 5 K platform, SPICEiST identified slightly more cell clusters when paired with 10x cell segmentation method at resolutions above 0.6, with no significant difference when using Proseg. These results suggest that SPICEiST resolves cell types with greater granularity when applied to datasets with smaller gene panels.

To evaluate the effect size of the difference between the SPICEiST and the GEX models across two distinct panel sizes (5 K and v1 platforms) and in two cell segmentation methods (10x and Proseg), the log mean ratio of the index for the SPICEiST relative to either SpaceFlow or GEX model was analyzed. All clustering indices demonstrated a trend suggesting a higher ratio on the v1 platform compared to the 5 K in ASW and CHI and a lower ratio in the DBI (Fig. 3C). However, the assortativity coefficient exhibited a reduced decline in the SPICEiST relative to the other models, in v1 compared to the 5 K platform. The trend was consistent across all conditions, with the exception of the SPICEiST versus GEX comparison utilizing the 10x segmentation method (Fig. 3C).

The findings from clustering performance metrics suggest that the SPICEiST is more effective for describing the intratumoral heterogeneity of cell states within tissue, particularly when using imaging-based ST with a small gene panel. However, this enhanced resolution of cell states does not extend to spatial organization. The model's capacity to precisely capture the spatial intermixture of cell types is, in most cases, more robust when a larger gene panel is utilized, although SPICEiST consistently produces more disorganized spatial patterns of clusters compared to other cell clustering methods.

### Resolving fine-grained cell subtypes in the Xenium lung cancer dataset

To assess the biological significance of the clustering results, homogeneity and completeness scores were calculated for nine patches, excluding those used for parameter optimization. These metrics quantify the correspondence between the Louvain clusters generated by SPICEiST and GEX and the annotated cell types, which are considered the ground truth. A high homogeneity score indicates that the clusters are pure, meaning they consist of cells from a single type. Conversely, a low completeness score signifies that a single annotated cell type is distributed across multiple clusters. Thus, high homogeneity combined with low completeness strongly indicates that the model successfully captures fine-grained cell subtypes that were not resolved by the initial cell type annotations.

Both SPICEiST and GEX demonstrated moderate cluster purity in Xenium v1 platform, achieving homogeneity scores above 0.55 for all resolutions except at 0.3 (Supplementary Fig. S6A). Notably, SPICEiST yielded significantly lower completeness scores than GEX. This combination of high homogeneity and low completeness indicates that SPICEiST can separate individual ground-truth cell types into multiple distinct clusters, demonstrating its ability to identify fine-grained cell subtypes. In contrast, there was no significant difference in homogeneity or completeness scores between SPICEiST and GEX on the Xenium 5 K platform (Supplementary Fig. S6B). This reflects greater performance improvement of SPICEiST in resolving fine-grained cell subtypes in v1 than on the Xenium 5 K platform.

### Performance assessment based on cell cluster granularity in the Xenium colon cancer dataset

**Fig. 4:**
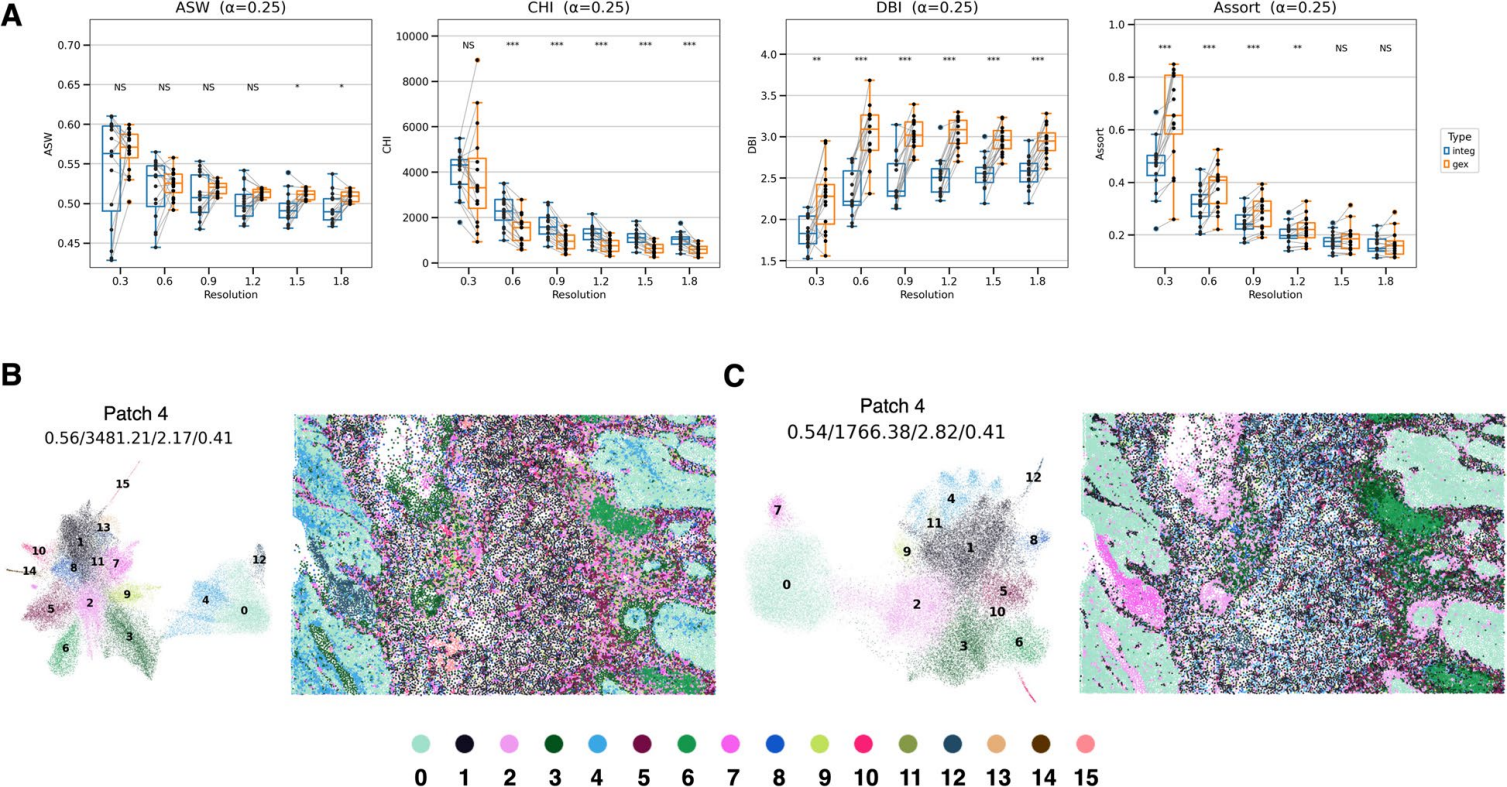
**Co**mparison of the SPICEiST clustering results with those obtained from GEX-based clustering in the Xenium human colorectal cancer dataset with v1 panel. **A** The boxplot compares three distinct clustering performance indices: ASW, CHI, DBI, and the assortativity coefficient across varying resolutions of cell clusters. The alpha weight for the loss was set to 0.25 during training. The stars above the boxplots show the results of statistical comparisons (Wilcoxon signed-rank test). Multiple comparison correction was performed using the Benjamini–Hochberg method. NS: Not significant, *: *p* $\le$ 0.05, **: *p* $\le$ 0.01, ***: *p* $\le$ 0.001, ****: *p* $\le$ 0.0001.. UMAP plots (left panel in each subplot) and the spatial distribution of cells (right panel in each subplot) illustrate the cell-level embedding and spatial distribution derived from (**B**) SPICEiST and (**C**) GEX-based clustering, respectively. The tissue sample was obtained from patch number 4, and the cluster resolution was set to 0.6. The four numerical values at the top of the plot represent ASW, CHI, DBI, and assortativity coefficient. Each dot in the plot represents cells, with the color indicating the identity of the clusters

In the Xenium colon cancer dataset, we compared the ASW, CHI, DBI, and assortativity coefficients between the SPICEiST and GEX methods, utilizing a panel size of 325. When quantitatively assessing clustering performance, the ASW indicated no significant differences between the SPICEiST and GEX models at cluster resolutions up to 1.2 (Fig. 4A). However, at higher resolutions, the SPICEiST demonstrated a notably lower ASW. In contrast, the CHI increased in the SPICEiST, while both the DBI and assortativity coefficients decreased when compared to the GEX model (Fig. 4A). These trends in CHI, DBI, and assortativity coefficients between SPICEiST and GEX were consistent with those observed in the human lung cancer Xenium v1 dataset, which featured a similarly sized gene panel.

Then, the UMAP visualization was performed at a cluster resolution of 0.6. This resolution revealed significant differences in the CHI, DBI, and assortativity coefficient between the two models (Fig. 4A). UMAP plots revealed a clearer separation among clusters in the SPICEiST than in the GEX model across 16 different patches (Fig. 4B, Cand Supplementary Fig. S7A, C). Furthermore, cells and their cluster identities in the 16 patches were mapped to their respective locations and compared between the two methods (Fig. 4B, C and Supplementary Fig. S7B, D). In comparison to the GEX model, the SPICEiST offered a more comprehensive depiction of the tumor microenvironment, showing the intricate intermixture between distinct cell types. For example, in patch number 4, which was selected from a total of 15 patches, cell clusters 0 and 4 from SPICEiST (Fig. 4B) were considered a single cluster in the GEX model (Fig. 4C). These clusters were both malignant cell subtypes with different functional implications (Supplementary Fig. S8).

### Resolving fine-grained cell subtypes in the Xenium colorectal cancer dataset

Both SPICEiST and GEX demonstrated moderate cluster purity, achieving homogeneity scores above 0.4 across all resolutions greater than 0.3 (Supplementary Fig. S9). Notably, at a resolution of 0.3, SPICEiST exhibited significantly higher scores compared to those of GEX. Besides, SPICEiST yielded significantly lower completeness scores than GEX at resolutions below 1.5. This combination of higher homogeneity and lower completeness suggests that SPICEiST effectively partitions ground-truth cell types into distinct clusters, a strength that is particularly evident at lower cluster granularities.

To investigate the biological significance of the cell clusters identified by SPICEiST, we performed a marker gene analysis on clusters 0, 4, and 12, which predominantly consisted of malignant cells (Supplementary Fig. S8A). Using a log fold change threshold of 1.0 and an adjusted p-value of 0.05, we identified marker genes for each cluster. Then, we performed Gene Ontology (GO) and Reactome pathway enrichment analyses. The analysis revealed distinct functional profiles: Cluster 0 was associated with fibronectin matrix formation and epithelial cell differentiation, Cluster 4 with regulation of vascular wound healing, and Cluster 12 with G1, S phase-specific transcription and one-carbon metabolic processes (Supplementary Fig. S8B). These distinctions in function between malignant cell clusters highlight the ability of SPICEiST to refine broad cell categories into more specific and biologically relevant subtypes.

### Performance assessment based on cell cluster granularity in the CosMx SMI lung cancer dataset

**Fig. 5:**
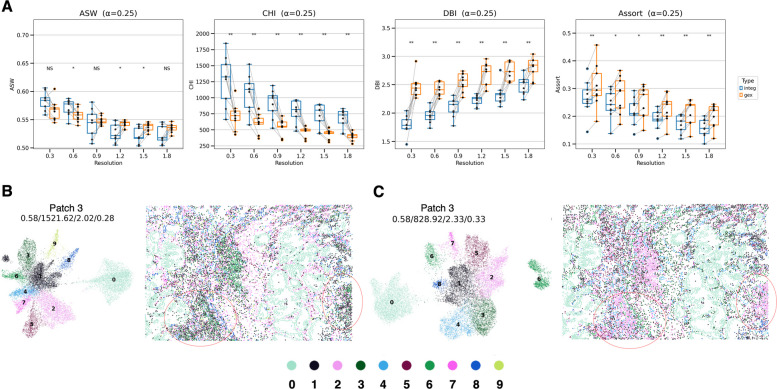
Comparison of the SPICEiST clustering results with those obtained from GEX-based clustering in the CosMx SMI human lung cancer dataset. **A** The boxplot compares three distinct clustering performance indices: ASW, CHI, DBI, and the assortativity coefficient across varying resolutions of cell clusters. The alpha weight for the loss was set to 0.25 during training. The stars above the boxplots show the results of statistical comparisons (Wilcoxon signed-rank test). Multiple comparison correction was performed using the Benjamini–Hochberg method. NS: Not significant, *: *p* $\le$ 0.05, **: *p* $\le$ 0.01, ***: *p* $\le$ 0.001, ****: *p* $\le$ 0.0001.. UMAP plots (left panel in each subplot) and the spatial distribution of cells (right panel in each subplot) illustrate the cell-level embedding and spatial distribution derived from (**B**) SPICEiST and (**C**) GEX-based clustering, respectively. The tissue sample was obtained from patch number 4, and the cluster resolution was set to 0.6. The four numerical values at the top of the plot represent ASW, CHI, DBI, and assortativity coefficient. Each dot in the plot represents cells, with the color indicating the identity of the cluster

The performance of the SPICEiST and GEX models was evaluated in lung adenocarcinoma tissue using another imaging-based ST platform, specifically the CosMx SMI, which has a panel size of 1,001 genes. The ASW, CHI, DBI, and assortativity coefficients between the integ and gex methods were compared (Fig. 5A). Notably, the ASW was significantly higher in the integ model at a resolution of 0.6, but it decreased at higher resolutions of 1.2 and 1.5. In contrast, the CHI showed an increase in the SPICEiST, while both the DBI and assortativity coefficients decreased when compared to the GEX model. The observed patterns in CHI, DBI, and assortativity coefficients exhibited congruence with those described in the human lung cancer Xenium dataset for v1 and 5 K panels.

Cell clusters from representative patch number 3, chosen from a total of 9 patches, were mapped to the corresponding tissue and compared across SPICEiST and GEX models (Fig. 5B, C). The cluster resolution of 0.6 was selected on the basis of its demonstration of significantly different values between the two models across all indices. Visual inspection revealed that the SPICEiST effectively represented the highly intermixed regions of cell infiltration that the GEX model failed to capture (red-circled regions in Fig. 5B, C). However, many of other patches did not show a clear distinction between the two models, which suggests that the SPICEiST may be less beneficial in a dataset with a large panel size (Supplementary Fig. S10).

### Resolving fine-grained cell subtypes in the CosMx SMI lung cancer dataset

Both SPICEiST and GEX demonstrated moderate cluster purity, with homogeneity scores increasing as the resolution increased (Supplementary Fig. S11). At resolutions greater than 0.3, both methods achieved scores above 0.4. However, scores of SPICEiST were significantly lower than those of GEX in this range. Additionally, SPICEiST yielded significantly lower completeness scores than GEX at resolutions greater than 0.9. This combination of high homogeneity and lower completeness indicates that SPICEiST may effectively partition ground-truth cell types into distinct clusters. This strength is particularly evident at higher cluster granularities.

## Discussion

Imaging-based ST has emerged as a vital platform for capturing single-cell gene expression profiles while preserving spatial context. However, its smaller coverage of genes along with the difficulties in cell segmentation, compared to single-cell RNA sequencing, limits its ability to accurately characterize cell states within tissues [7]. One feasible approach to overcoming these limitations is leveraging subcellular gene expression patterns, which reflect transcriptional processing and regulation [16, 17]. In heterogeneous tissues like tumors, subtle differences in cell states are often indistinguishable to conventional methods because the cells may have similar overall gene expression levels. However, their distinct functional states can be revealed by unique RNA localization patterns linked to processing, transport, and localized translation [17]. In complex environments where cell states are not defined by RNA abundance alone, this additional subcellular information can be crucial for achieving a more accurate and interpretable mapping of cell identity.

In this regard, we employed a graph autoencoder to integrate cell-level gene expression profiles with subcellular transcript patterns at a resolution of 2 μm. This approach aimed to identify an optimal representation of cellular states that would effectively elucidate the tumor microenvironment. Using the 10x cell segmentation method, SPICEiST consistently outperformed the conventional cell-level gene expression-based model (GEX) and SpaceFlow in clustering performance, as indicated by the CHI and DBI (Figs. 2C and 3A). Using Proseg for cell segmentation, SPICEiST demonstrated superior CHI and DBI values, especially in imaging-based ST platforms with small gene panels, such as Xenium v1. The findings suggest that SPICEiST provides benefits in cell clustering, particularly in datasets with small gene panels, even when the state-of-the-art cell segmentation tool is applied. This effectively isolates the benefits of SPICEiST from the quality of the initial cell masks, thereby confirming the unique contribution of the method to enhancing clustering. Conversely, the clustering index ASW, which reflects the compactness of cells within the feature space, did not exhibit a consistent trend across the dataset (Figs. 2C, 3A, 4A, and 5A). However, there was a noticeable trend where the median value of the SPICEiST was relatively lower than that of the GEX model as the cluster resolution increased. This suggests that as the resolution of the cell clusters rises, subcellular gene expression patterns may lead to over-clustering, resulting in less compact clusters for the SPICEiST model, while the GEX model maintains better compactness in the feature space, leading to a relative decrease in ASW for SPICEiST.

The spatially clustered patterns, as measured by the assortativity coefficient, consistently demonstrated a trend of lower values in the SPICEiST compared to the GEX and SpaceFlow, regardless of the gene panel sizes (Figs. 2C and 3A). This observation suggests that the SPICEiST may offer a more detailed representation of the cellular organization of the tissue compared to the GEX and SpaceFlow, which is characterized by a more dispersed arrangement of cells. Therefore, it can be inferred that the SPICEiST model generally effectively represents disorganization of cells, possibly influenced by tumor heterogeneity.

The performance of the SPICEiST was quantitatively compared based on gene panel size within the same lung cancer tissue (Fig. 3C). The results indicated that both ASW and CHI demonstrated a greater increase in index ratios when using a smaller panel in comparison to other cell clustering methods. Conversely, DBI exhibited a greater decrease in ratios under the same conditions. These findings suggest that the additive effects of integrating subcellular expression patterns are more pronounced in imaging-based ST when a smaller gene panel is utilized. On the other hand, an examination of the spatial clustering pattern as indicated by the assortativity coefficient suggests that the SPICEiST model accentuates spatial disorganization to a lesser extent in the smaller panel relative to the larger one, except for the case when 10x cell segmentation method is used and compared with GEX. This enhancement may result in a less comprehensive explanation of the spatial intermixture of cells and the heterogeneous nature of tumors in the small panel dataset, which may indicate limitations.

In addition to the unsupervised cluster analysis, we performed a supervised evaluation to assess the biological relevance of the clustering results. Using cell type annotations obtained using TACCO algorithm as a reference, we compared the correspondence between the labels and the clusters generated by SPICEiST and GEX. This analysis revealed that SPICEiST excels at resolving fine-grained cellular identities (Supplementary Figs. 6, 9, and 11). While the GEX model often grouped broad, annotated cell types into single clusters, SPICEiST successfully partitioned these same populations into multiple, more granular clusters. Subsequent functional enrichment analysis confirmed that these refined subtypes were not arbitrary divisions, but rather represented distinct biological states with unique pathway associations. These results demonstrate superior ability of SPICEiST to uncover meaningful cellular heterogeneity by integrating subcellular transcript patterns.

There are additional considerations to keep in mind when interpreting the trends in index differences and their implications. The observed variations in trends for feature space-based indices, such as CHI, DBI, and ASW, can be attributed to the unique local patterns of the clusters. For example, non-convex and elongated cluster shapes, along with the presence of outliers, can significantly affect ASW and CHI more than DBI, thereby influencing overall outcomes [35, 36]. Consequently, the trends of relatively lower ASW in the SPICEiST at higher resolutions may stem from the elongated distribution patterns of fine clusters within the feature space.

## Conclusions

The limitations of imaging-based ST can be addressed by utilizing subcellular gene expression patterns alongside cell-level gene expression profiles through a graph autoencoder, SPICEiST. This approach proves especially effective in distinguishing subtle difference in cell states within small gene panel-based imaging ST platforms, thereby enhancing the understanding of tumor heterogeneity.

## Supplementary Information

Additional file 1: Supplementary Figures. Figure S1. Comparison of cell clustering performance according to changes in loss weight and grid size. Figure S2. Visualization of the SPICEiST clustering results compared to the GEX-based clustering in the Xenium lung cancer dataset with the v1 panel and 10x cell segmentation at a resolution of 0.3. Figure S3. Visualization of the SPICEiST clustering results compared to the SpaceFlow and GEX-based clustering in the Xenium lung cancer dataset with the v1 panel and 10x cell segmentation at a resolution of 0.6. Figure S4. Visualization of the SPICEiST clustering results compared to the GEX clustering in the Xenium lung cancer dataset with the 5 K panel and 10x cell segmentation method at a resolution of 0.3. Figure S5. Comparison of the cluster number from SPICEiST, SpaceFlow, and GEX across different imaging-based ST platforms (v1 and Prime 5 K) and cell segmentation methods in the Xenium human lung cancer dataset. Figure S6. Comparison of the homogeneity and completeness scores from SPICEiST and GEX across different imaging-based ST platforms (v1 and Prime 5 K) in the Xenium human lung cancer dataset. Figure S7. Visualization of the SPICEiST clustering results compared to the GEX-based clustering in the Xenium colorectal cancer dataset with the v1 panel and 10x cell segmentation method at a resolution of 0.6. Figure S8. Analysis of cell type correspondence and functional implications of malignant cell subtypes in patch number 4 of the Xenium colorectal cancer dataset. Figure S9. Comparison of the homogeneity and completeness scores from SPICEiST and GEX in the Xenium colorectal cancer dataset. Figure S10. Visualization of the SPICEiST clustering results compared to the GEX-based clustering in the CosMx SMI lung cancer dataset with 10x cell segmentation method at a resolution of 0.6. Figure S11. Comparison of the homogeneity and completeness scores from SPICEiST and GEX in the CosMx SMI lung cancer dataset.

## Data Availability

Imaging-based ST datasets were utilized for the training and evaluation of the SPICIEiST model. Xenium ST datasets for lung adenocarcinoma tissue were acquired from both the v1 and Prime 5 K platforms. Both datasets were downloaded from the 10x Genomics dataset repository (https://www.10xgenomics.com/datasets/xenium-human-lung-cancer-post-xenium-technote). Additionally, the Xenium v1 dataset from a colon adenocarcinoma patient was downloaded (https://www.10xgenomics.com/datasets/human-colon-preview-data-xenium-human-colon-gene-expression-panel-1-standard). Furthermore, the CosMx SMI dataset from a lung adenocarcinoma patient, designated Lung 5–1, was retrieved from the NanoString dataset repository (https://nanostring.com/products/cosmx-spatial-molecular-imager/ffpe-dataset/) [19]. Single-cell RNA-seq datasets for the lung adenocarcinoma patients were downloaded from the GSE131907 ( https://www.ncbi.nlm.nih.gov/geo/query/acc.cgi?acc=GSE131907) and colon adenocarcinoma from the 3CA repository (https://www.weizmann.ac.il/sites/3CA/). Source codes (in Python) for SPICIEiST are accessible at https://github.com/portrai-io/SPICEiST.

## Abbreviations

Assort: Assortativity coefficient
ASW: Average silhouette width
CHI: Calinski-Harabasz index
CS: Completeness score
DBI: Davies-Bouldin index
HS: Homogeneity score
GEX: Conventional method which uses cell-level gene expression profiles for cell clustering
ST: Spatial Transcriptomics
