# Supplementary material for: SPICEiST: subcellular RNA pattern enhances cell clustering of imaging-based spatial transcriptomics
Source: Genomics Inform. 2025 Dec 2;23:23. doi: 10.1186/s44342-025-00056-1 (PMC12670746; doi:10.1186/s44342-025-00056-1)
Supplement: Supplementary file 1 — Additional file 1: Supplementary Figures. Figure S1. Comparison of cell clustering performance according to changes in loss weight and grid size. Figure S2. Visualization of the SPICEiST clustering results compared to the GEX-based clustering in the Xenium lung cancer dataset with the v1 panel and 10x cell segmentation at a resolution of 0.3. Figure S3. Visualization of the SPICEiST clustering results compared to the SpaceFlow and GEX-based clustering in the Xenium lung cancer dataset with the v1 panel and 10x cell segmentation at a resolution of 0.6. Figure S4. Visualization of the SPICEiST clustering results compared to the GEX clustering in the Xenium lung cancer dataset with the 5 K panel and 10x cell segmentation method at a resolution of 0.3. Figure S5. Comparison of the cluster number from SPICEiST, SpaceFlow, and GEX across different imaging-based ST platforms (v1 and Prime 5 K) and cell segmentation methods in the Xenium human lung cancer dataset. Figure S6. Comparison of the homogeneity and completeness scores from SPICEiST and GEX across different imaging-based ST platforms (v1 and Prime 5 K) in the Xenium human lung cancer dataset. Figure S7. Visualization of the SPICEiST clustering results compared to the GEX-based clustering in the Xenium colorectal cancer dataset with the v1 panel and 10x cell segmentation method at a resolution of 0.6. Figure S8. Analysis of cell type correspondence and functional implications of malignant cell subtypes in patch number 4 of the Xenium colorectal cancer dataset. Figure S9. Comparison of the homogeneity and completeness scores from SPICEiST and GEX in the Xenium colorectal cancer dataset. Figure S10. Visualization of the SPICEiST clustering results compared to the GEX-based clustering in the CosMx SMI lung cancer dataset with 10x cell segmentation method at a resolution of 0.6. Figure S11. Comparison of the homogeneity and completeness scores from SPICEiST and GEX in the CosMx SMI lung cancer dataset. [file 44342_2025_56_MOESM1_ESM.pdf]

SPICEiST: Subcellular RNA Pattern Enhances Cell Clustering of Imaging-Based Spatial Transcriptomics

Sungwoo Bae[1, *], Yuchang Seong[1], Dongjoo Lee[1], Hongyoon Choi[1, 2, *]

[1]Portrai, Inc. Republic of Korea; [2]Department of Nuclear Medicine, Seoul National University Hospital, Seoul, Republic of Korea

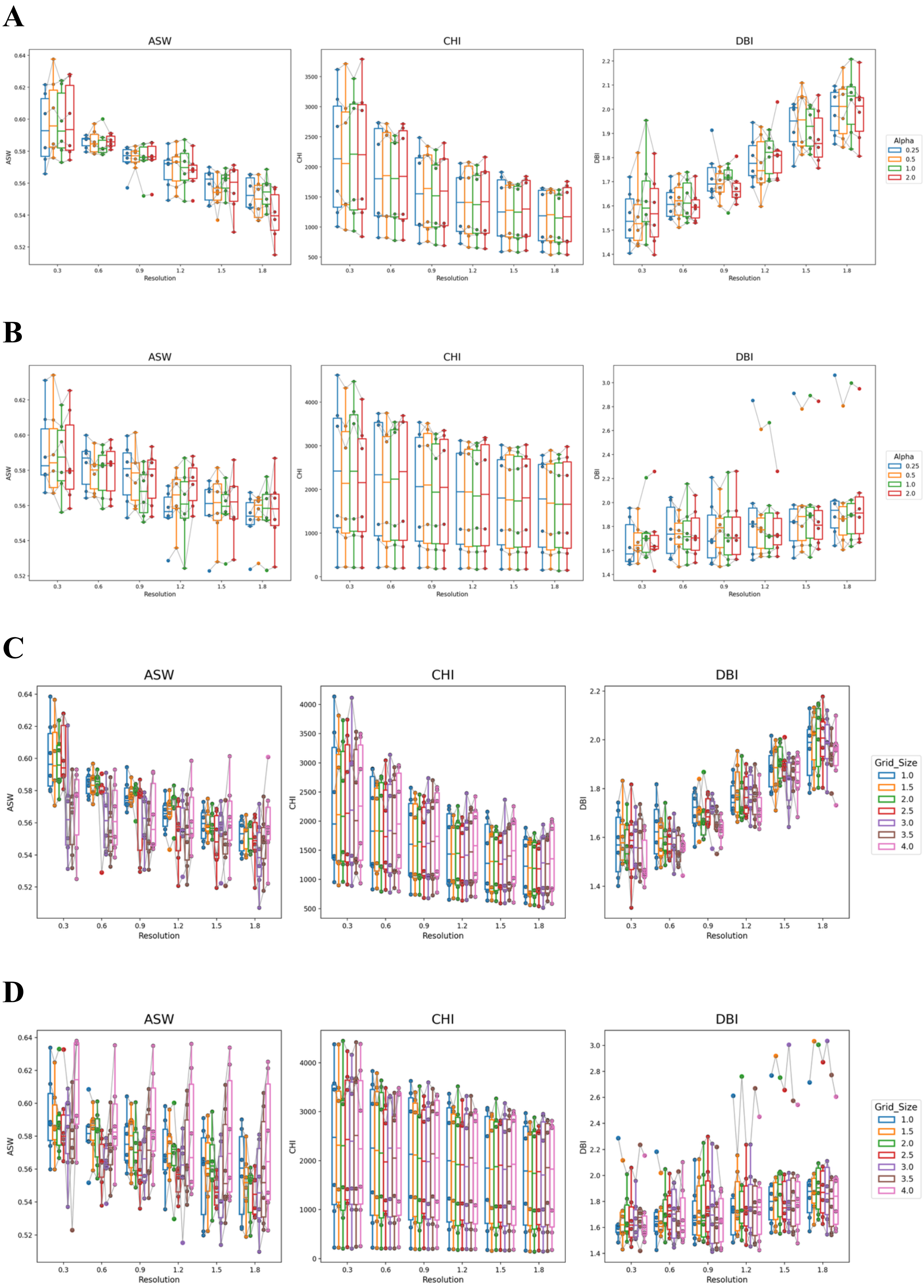
A
ASW
CHI
DBI
Resolution
Alpha
0.25
0.5
1.0
2.0
B
ASW
CHI
DBI
Resolution
Alpha
0.25
0.5
1.0
2.0
C
ASW
CHI
DBI
Resolution
Grid_Size
1.0
1.5
2.0
2.5
3.0
3.5
4.0
D
ASW
CHI
DBI
Resolution
Grid_Size
1.0
1.5
2.0
2.5
3.0
3.5
4.0

Supplementary Figure S1. Comparison of cell clustering performance according to changes in loss weight and grid size.

The boxplots compare three distinct clustering performance indices: ASW, CHI, and DBI — across different alpha ranges (0.25, 0.5, 1.0, and 2.0) and resolutions of cell clusters in (A) Xenium v1 and (B) Prime 5K datasets. Additionally, the boxplots compare the three performance indices across different grid sizes (1.0, 1.5, 2.0, 2.5, 3.0, and 4.0 μm) and varying cell cluster resolutions in the Xenium v1 (C) and Prime 5K (D) datasets.

A

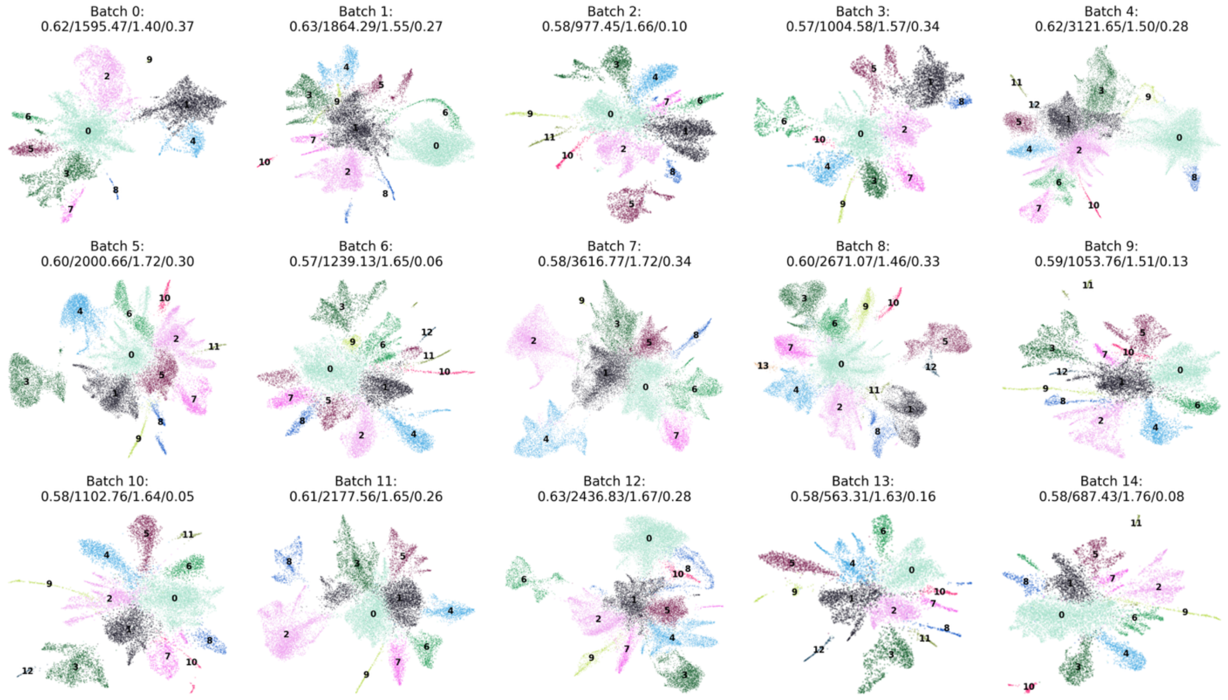
Batch 0:
0.62/1595.47/1.40/0.37
Batch 1:
0.63/1864.29/1.55/0.27
Batch 2:
0.58/977.45/1.66/0.10
Batch 3:
0.57/1004.58/1.57/0.34
Batch 4:
0.62/3121.65/1.50/0.28
Batch 5:
0.60/2000.66/1.72/0.30
Batch 6:
0.57/1239.13/1.65/0.06
Batch 7:
0.58/3616.77/1.72/0.34
Batch 8:
0.60/2671.07/1.46/0.33
Batch 9:
0.59/1053.76/1.51/0.13
Batch 10:
0.58/1102.76/1.64/0.05
Batch 11:
0.61/2177.56/1.65/0.26
Batch 12:
0.63/2436.83/1.67/0.28
Batch 13:
0.58/563.31/1.63/0.16
Batch 14:
0.58/687.43/1.76/0.08

B

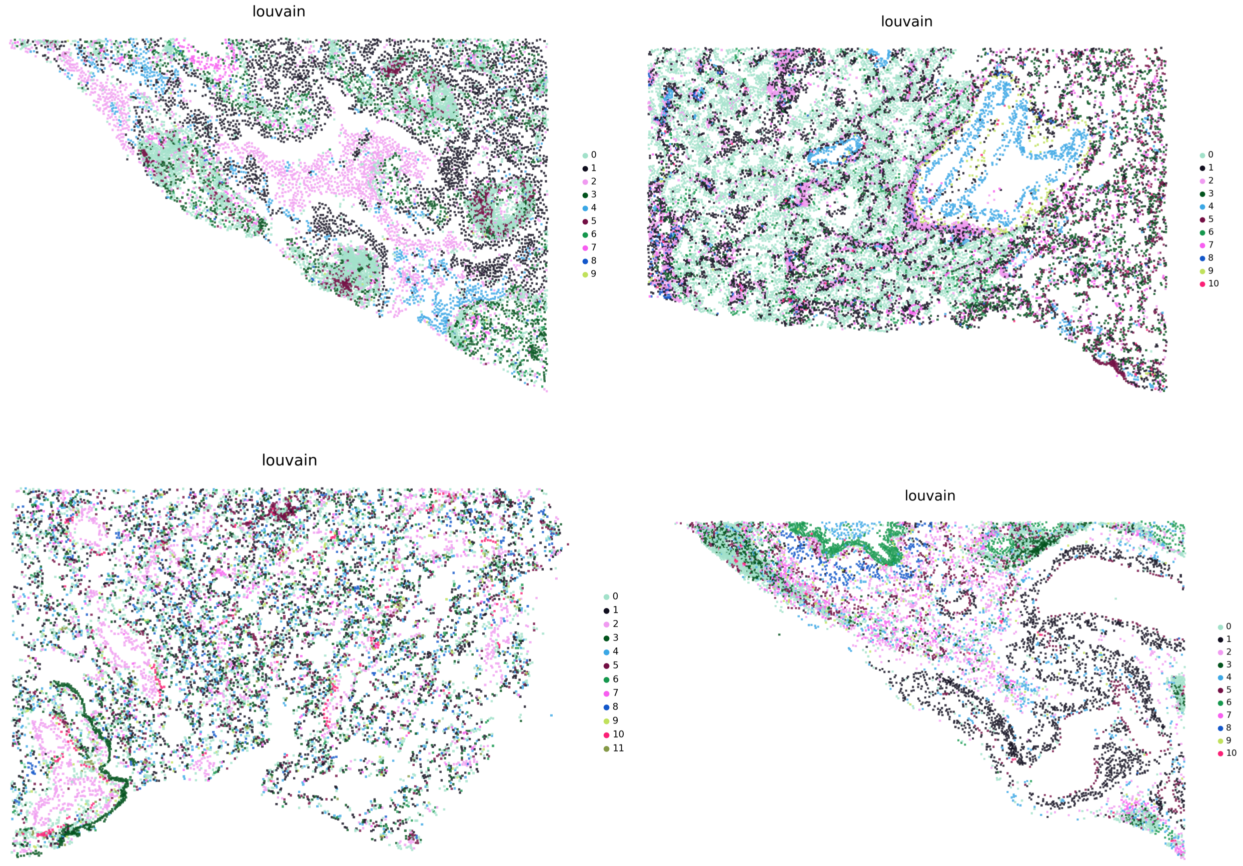
louvain
louvain
louvain
louvain

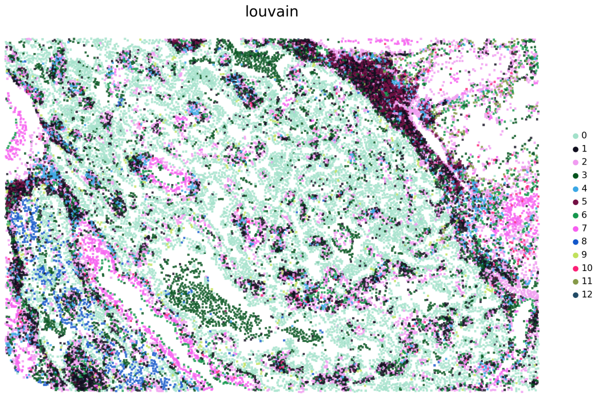
louvain
0
1
2
3
4
5
6
7
8
9
10
11
12

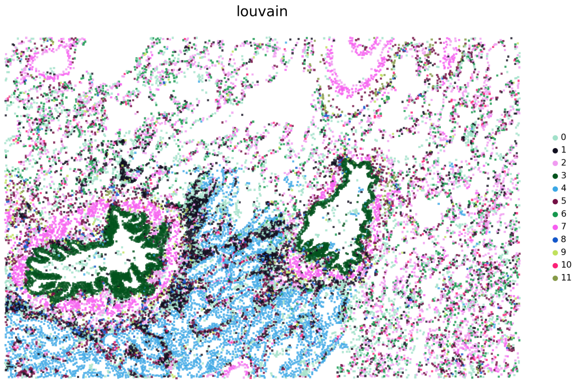
louvain
0
1
2
3
4
5
6
7
8
9
10
11

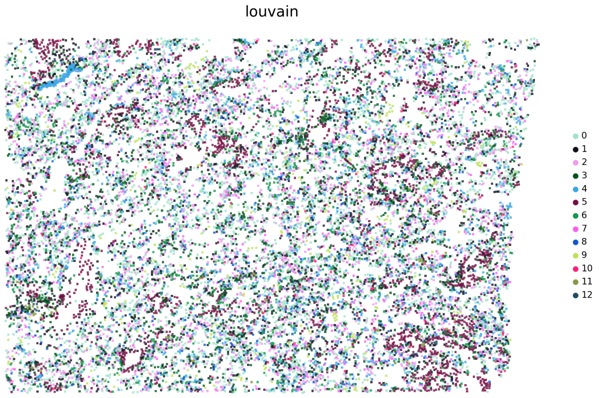
louvain
0
1
2
3
4
5
6
7
8
9
10
11
12

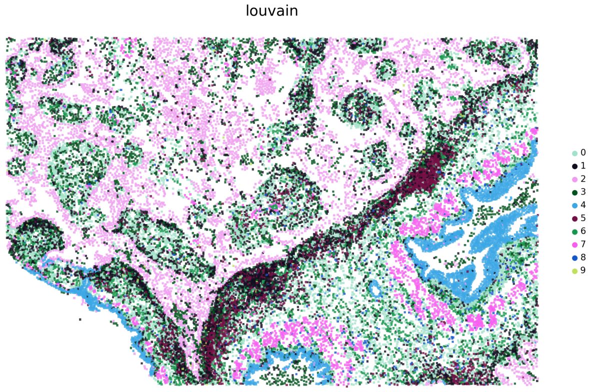
louvain
0
1
2
3
4
5
6
7
8
9

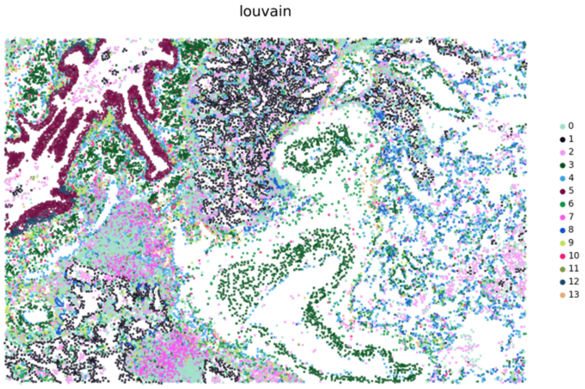
louvain
0
1
2
3
4
5
6
7
8
9
10
11
12
13

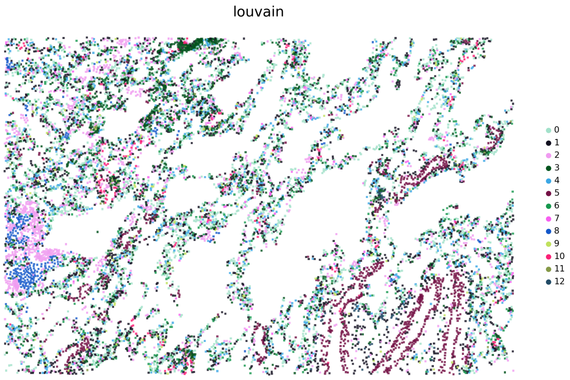
louvain
0
1
2
3
4
5
6
7
8
9
10
11
12

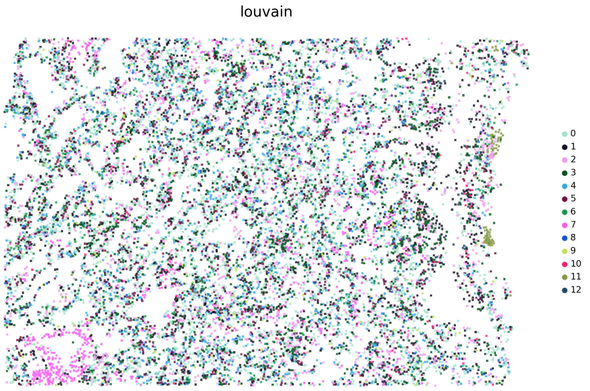
louvain
0
1
2
3
4
5
6
7
8
9
10
11
12

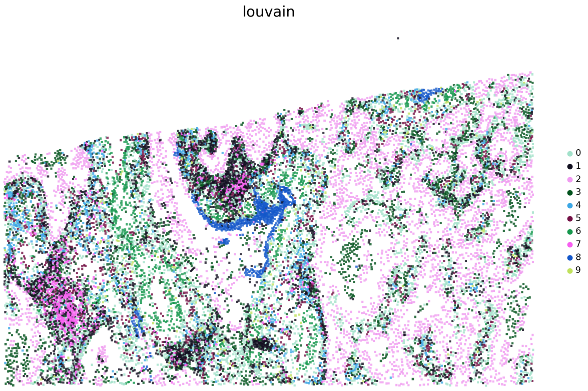
louvain
0
1
2
3
4
5
6
7
8
9

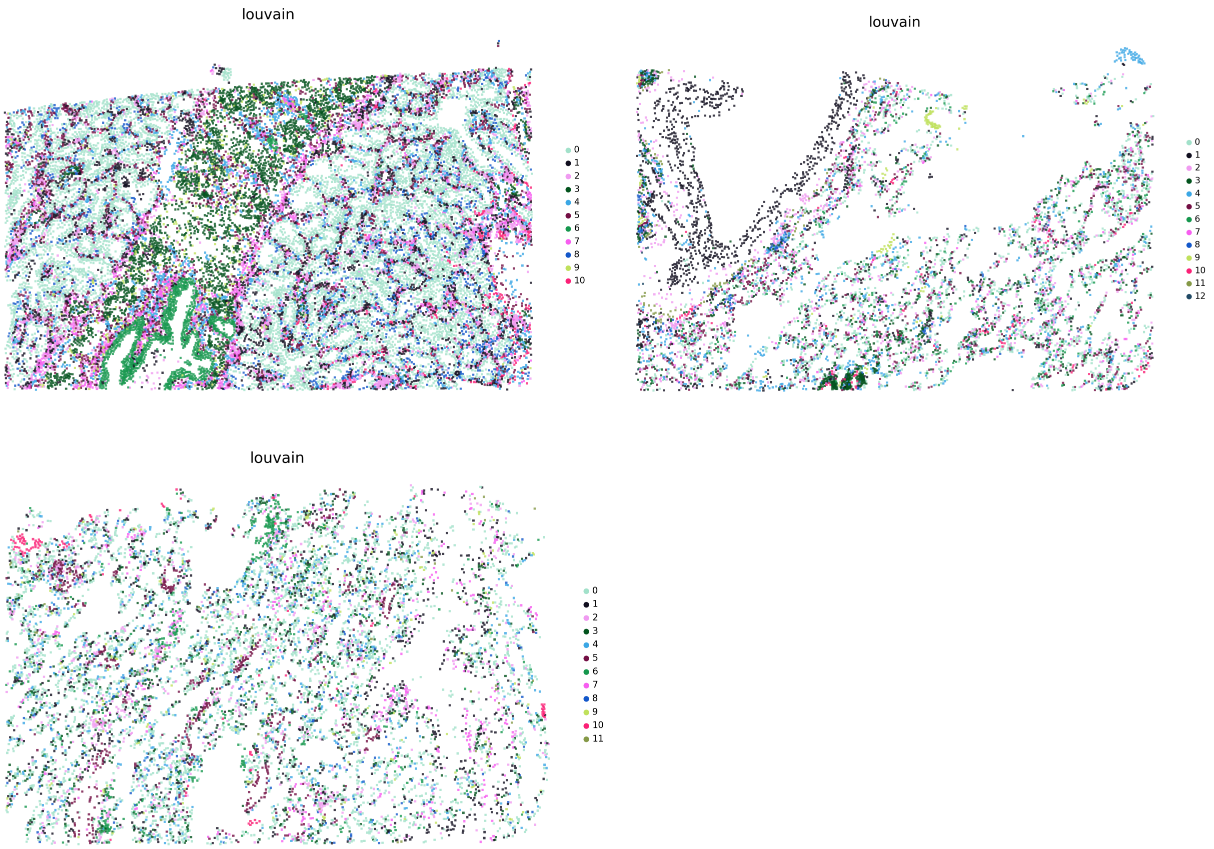
louvain
0 1 2 3 4 5 6 7 8 9 10
louvain
0 1 2 3 4 5 6 7 8 9 10 11 12
louvain
0 1 2 3 4 5 6 7 8 9 10 11

C

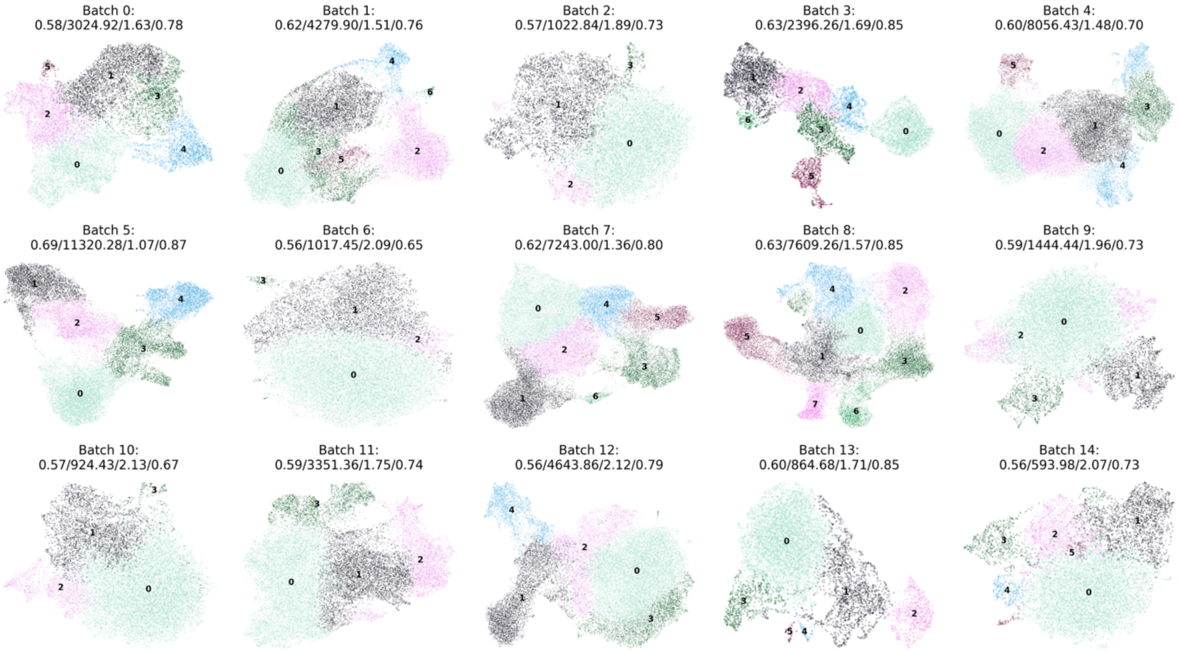
Batch 0:
0.58/3024.92/1.63/0.78
Batch 1:
0.62/4279.90/1.51/0.76
Batch 2:
0.57/1022.84/1.89/0.73
Batch 3:
0.63/2396.26/1.69/0.85
Batch 4:
0.60/8056.43/1.48/0.70
Batch 5:
0.69/11320.28/1.07/0.87
Batch 6:
0.56/1017.45/2.09/0.65
Batch 7:
0.62/7243.00/1.36/0.80
Batch 8:
0.63/7609.26/1.57/0.85
Batch 9:
0.59/1444.44/1.96/0.73
Batch 10:
0.57/924.43/2.13/0.67
Batch 11:
0.59/3351.36/1.75/0.74
Batch 12:
0.56/4643.86/2.12/0.79
Batch 13:
0.60/864.68/1.71/0.85
Batch 14:
0.56/593.98/2.07/0.73

D

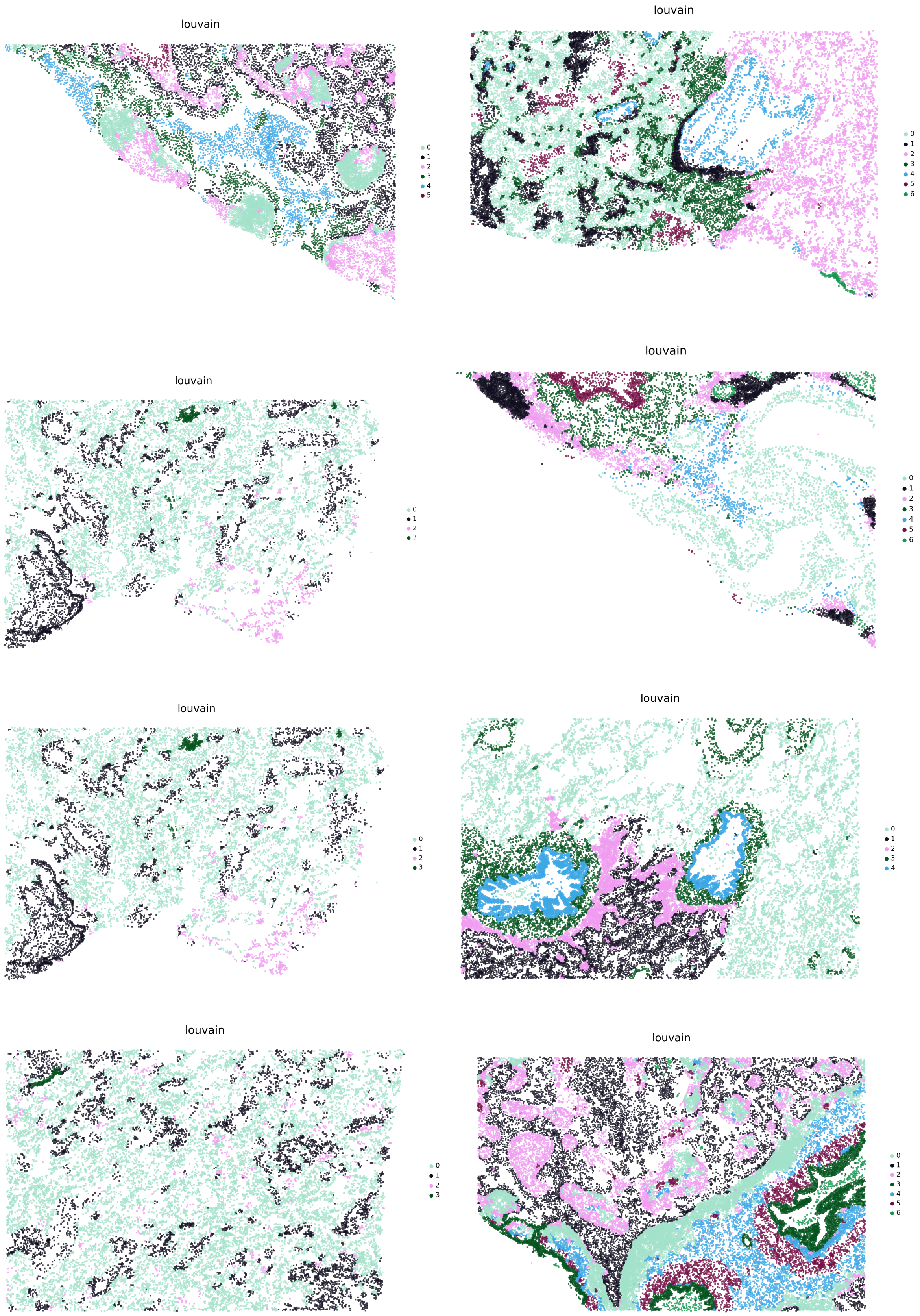
louvain
0 1 2 3 4 5
louvain
0 1 2 3 4 5 6
louvain
0 1 2 3
louvain
0 1 2 3 4 5 6
louvain
0 1 2 3
louvain
0 1 2 3 4
louvain
0 1 2 3
louvain
0 1 2 3 4 5 6

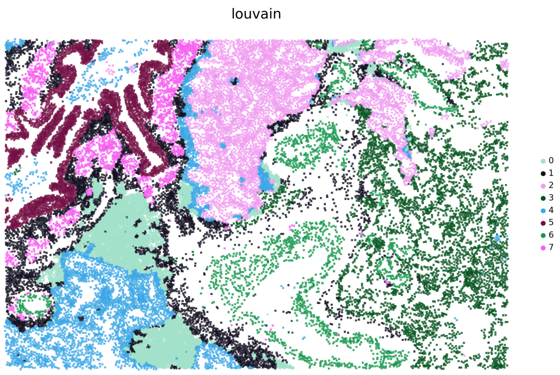
louvain
0
1
2
3
4
5
6
7

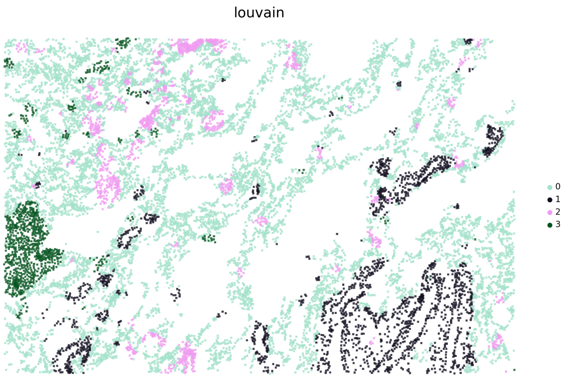
louvain
0
1
2
3

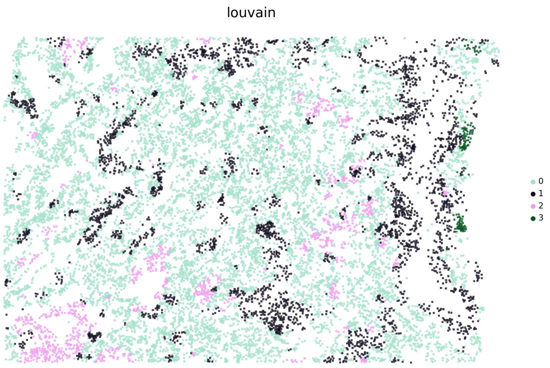
louvain
0
1
2
3

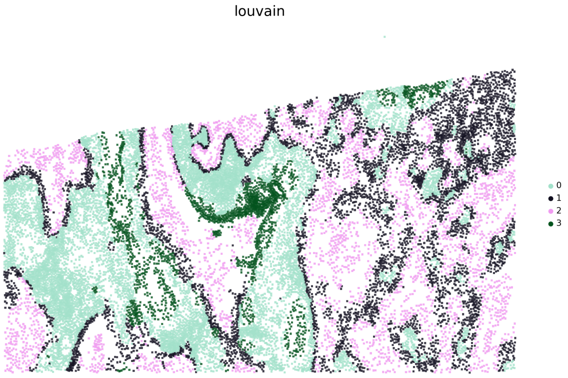
louvain
0
1
2
3

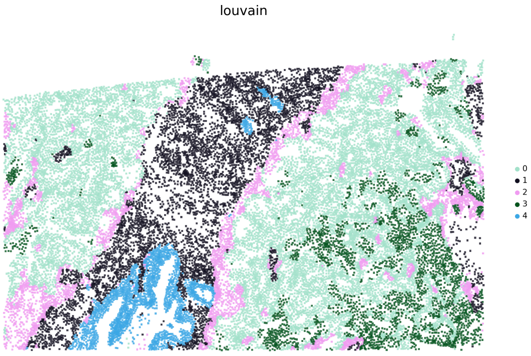
louvain
0
1
2
3
4

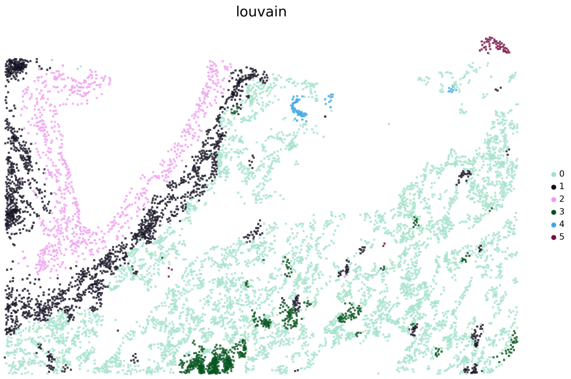
louvain
0
1
2
3
4
5

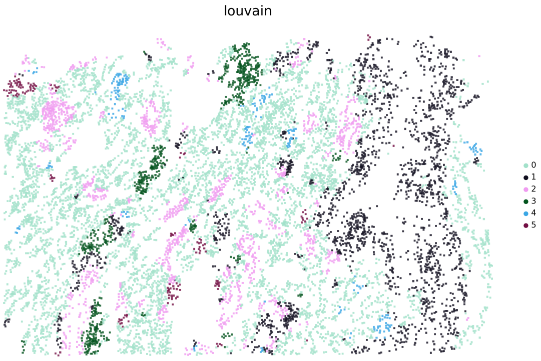
louvain
0
1
2
3
4
5

E

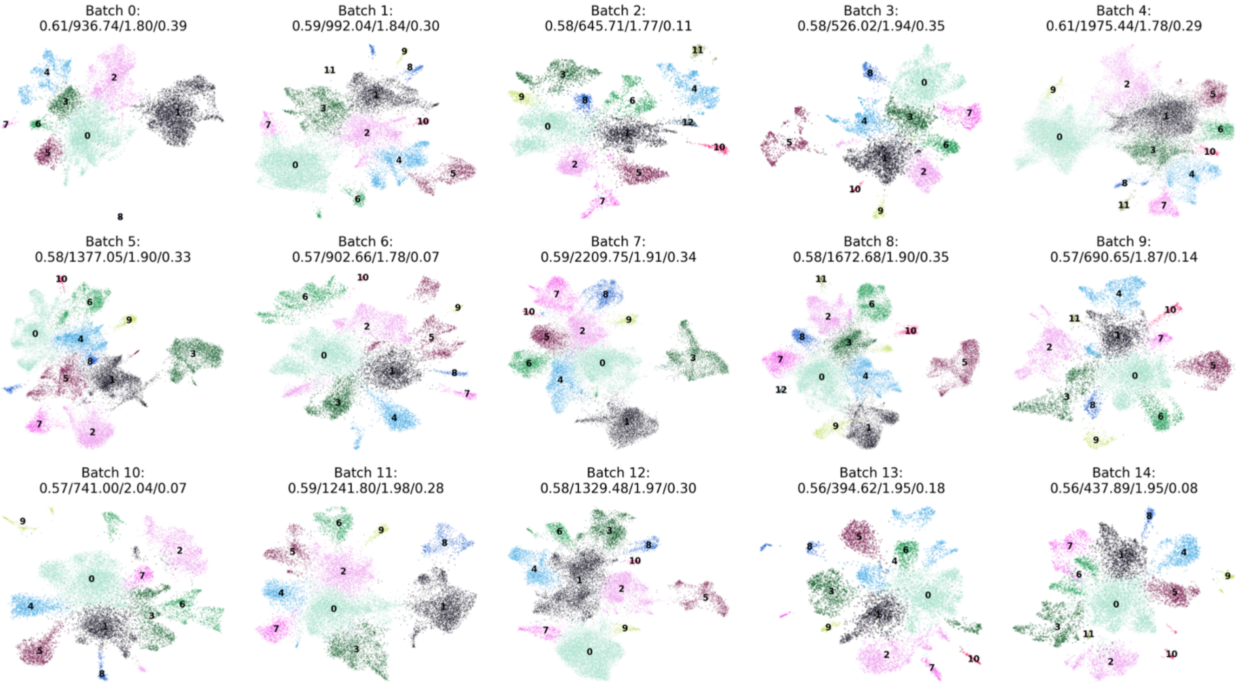
Batch 0:
0.61/936.74/1.80/0.39
Batch 1:
0.59/992.04/1.84/0.30
Batch 2:
0.58/645.71/1.77/0.11
Batch 3:
0.58/526.02/1.94/0.35
Batch 4:
0.61/1975.44/1.78/0.29
Batch 5:
0.58/1377.05/1.90/0.33
Batch 6:
0.57/902.66/1.78/0.07
Batch 7:
0.59/2209.75/1.91/0.34
Batch 8:
0.58/1672.68/1.90/0.35
Batch 9:
0.57/690.65/1.87/0.14
Batch 10:
0.57/741.00/2.04/0.07
Batch 11:
0.59/1241.80/1.98/0.28
Batch 12:
0.58/1329.48/1.97/0.30
Batch 13:
0.56/394.62/1.95/0.18
Batch 14:
0.56/437.89/1.95/0.08

F

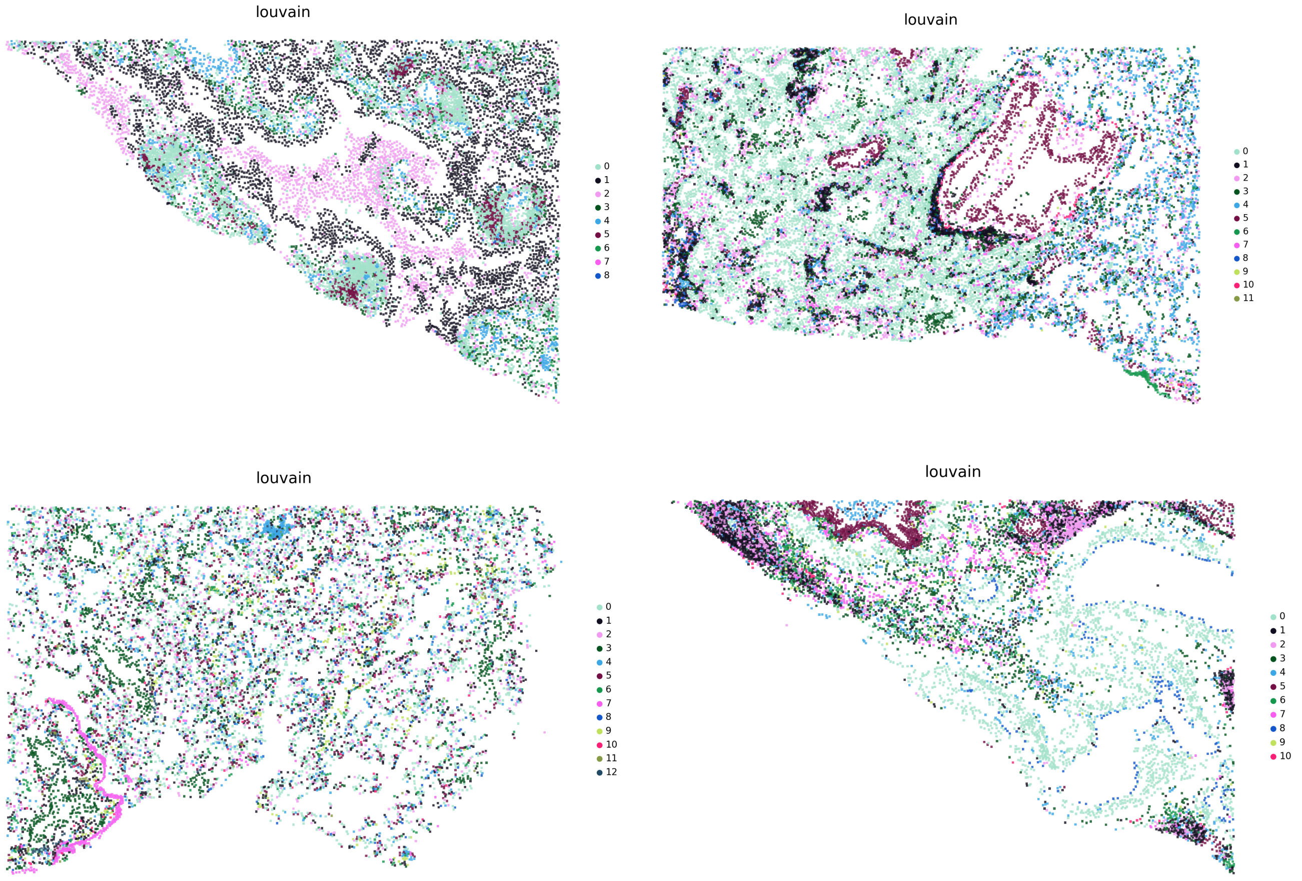
louvain
louvain
louvain
louvain

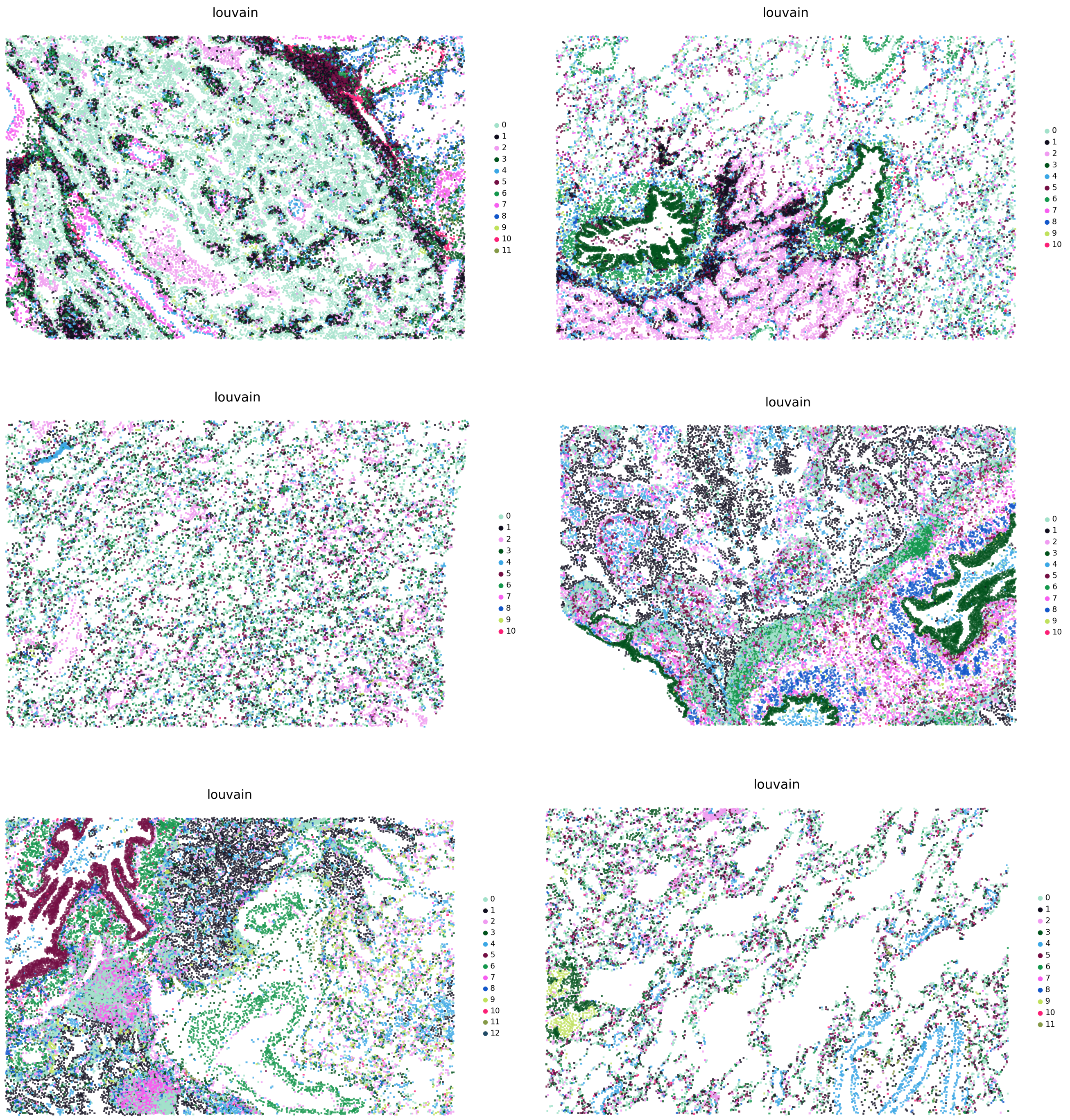
louvain
0
1
2
3
4
5
6
7
8
9
10
11
louvain
0
1
2
3
4
5
6
7
8
9
10
louvain
0
1
2
3
4
5
6
7
8
9
10
louvain
0
1
2
3
4
5
6
7
8
9
10
louvain
0
1
2
3
4
5
6
7
8
9
10
11
12
louvain
0
1
2
3
4
5
6
7
8
9
10
11

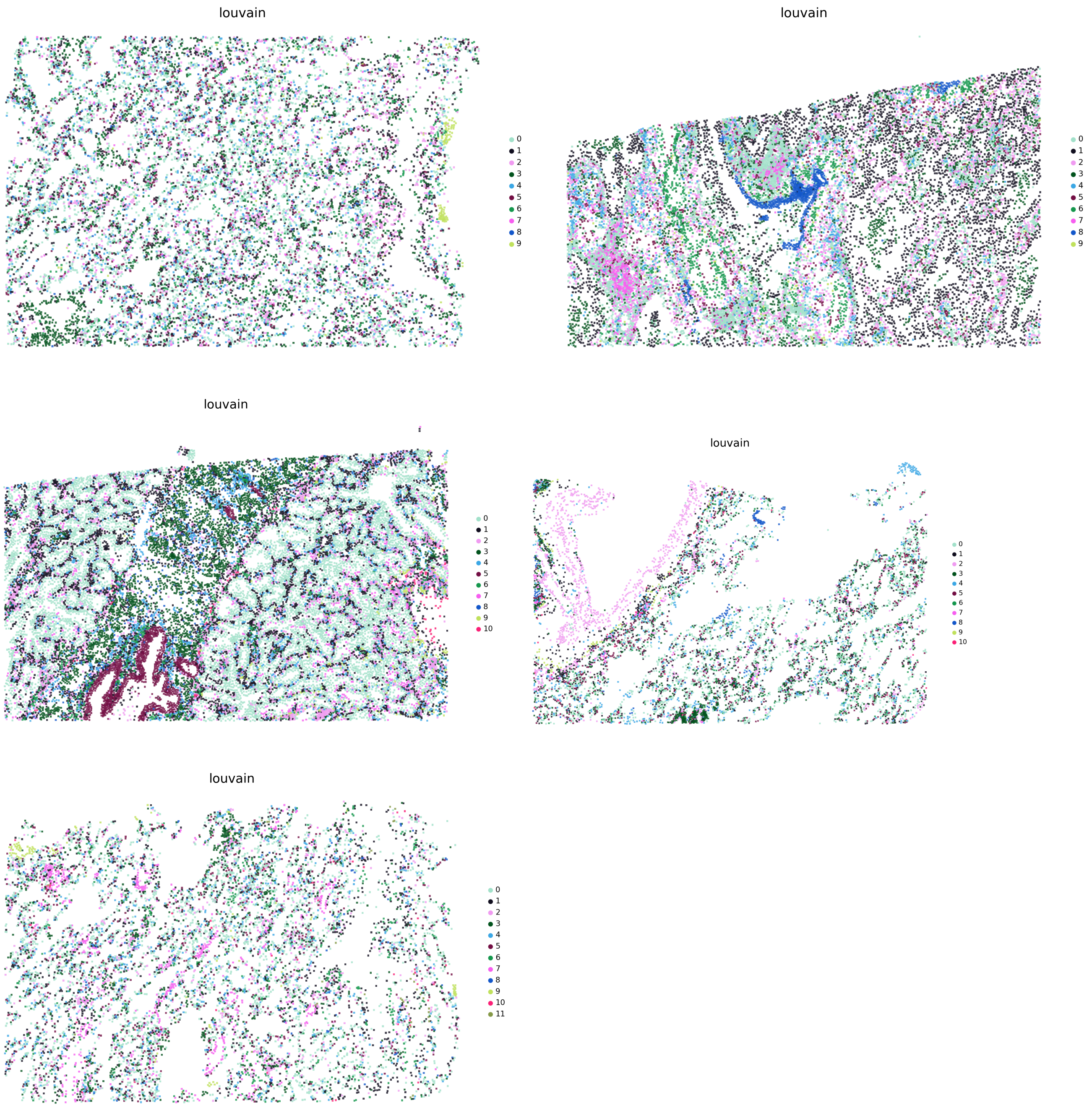

Supplementary Figure S2. Visualization of the SPICEiST clustering results compared to the GEX-based clustering in the Xenium lung cancer dataset with the v1 panel and 10x cell segmentation at a resolution of 0.3.

UMAP plots and the spatial distribution of cells illustrate the cell-level embedding and spatial distribution derived from SPICEiST-based analysis (SPICEiST; A: UMAP, B: spatial distribution of cells), SpaceFlow (SpaceFlow; C: UMAP, D: spatial distribution of cells), and cell-level gene expression-based clustering (GEX; E: UMAP, F: spatial distribution of cells), respectively. The tissue samples, obtained from patch numbers 0 to 14, are presented serially from left to right and top to bottom. The four numerical values at the top of the UMAP plot represent the ASW, CHI, DBI, and assortativity coefficient. Each dot in the plot corresponds to a cell, with color indicating the identity of the clusters.

A

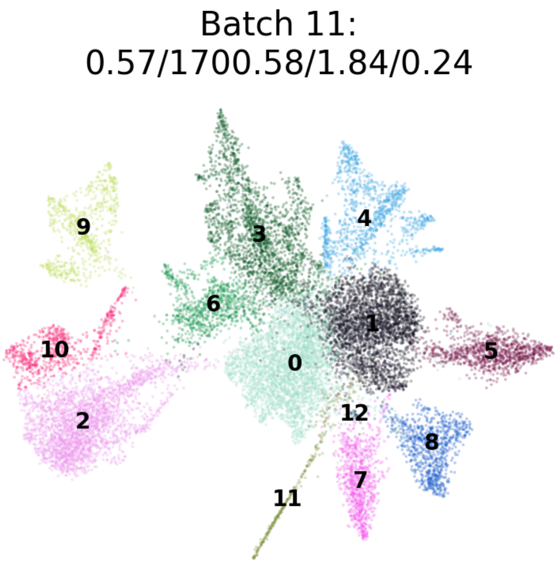

Batch 11:
0.57/1700.58/1.84/0.24
9
3
4
6
1
10
0
5
2
12
8
7
11

B

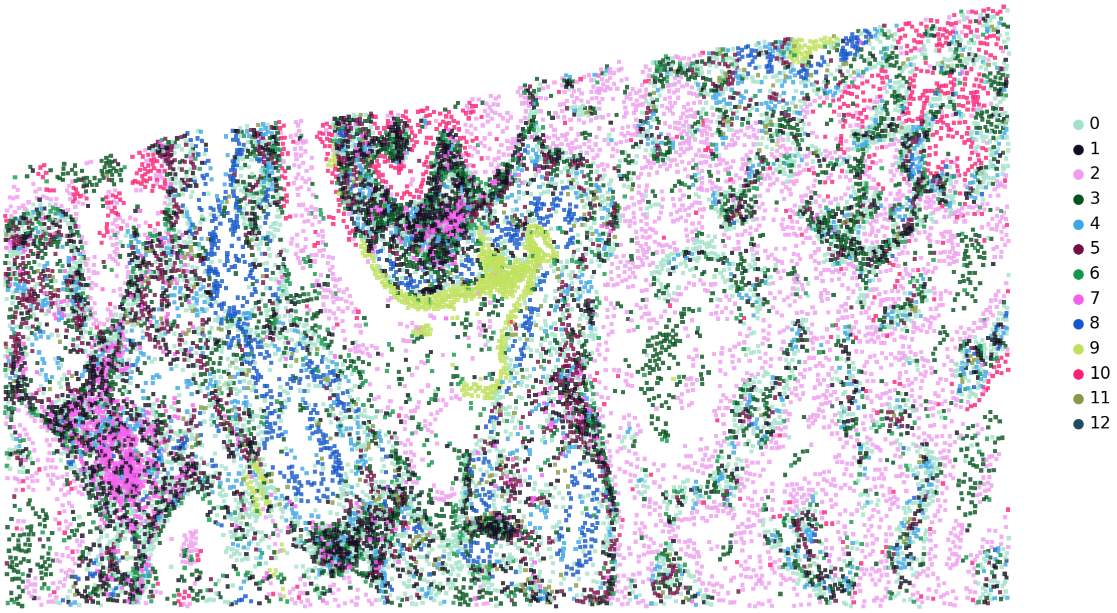

0
1
2
3
4
5
6
7
8
9
10
11
12

C

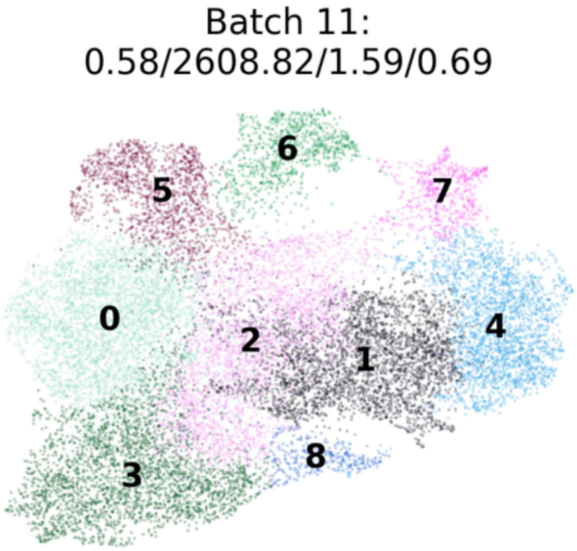

D

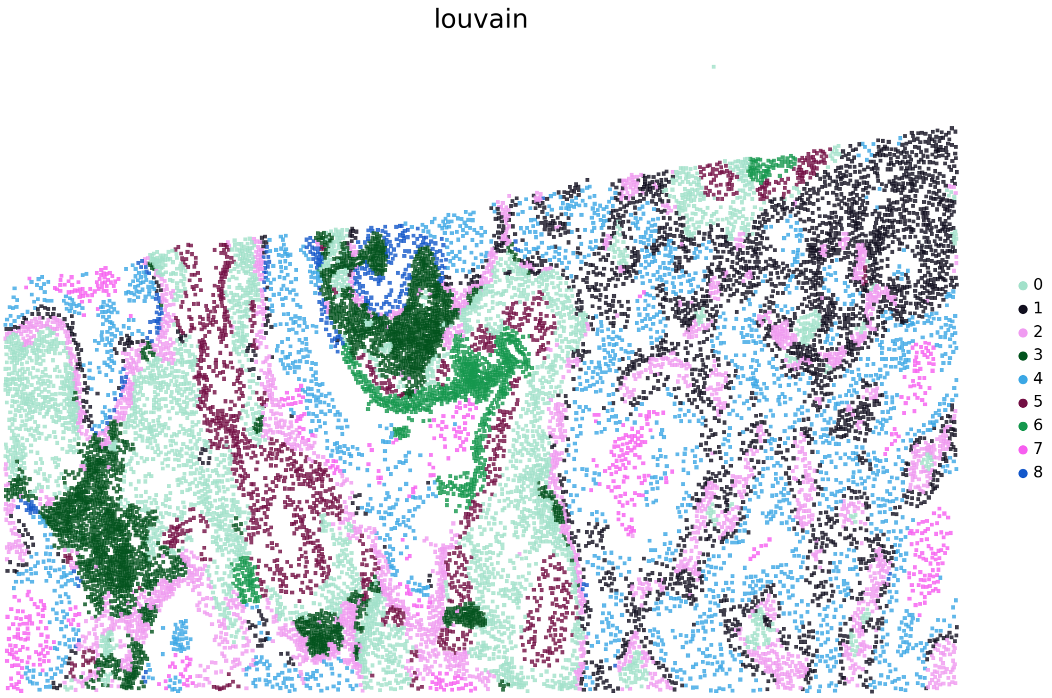

E

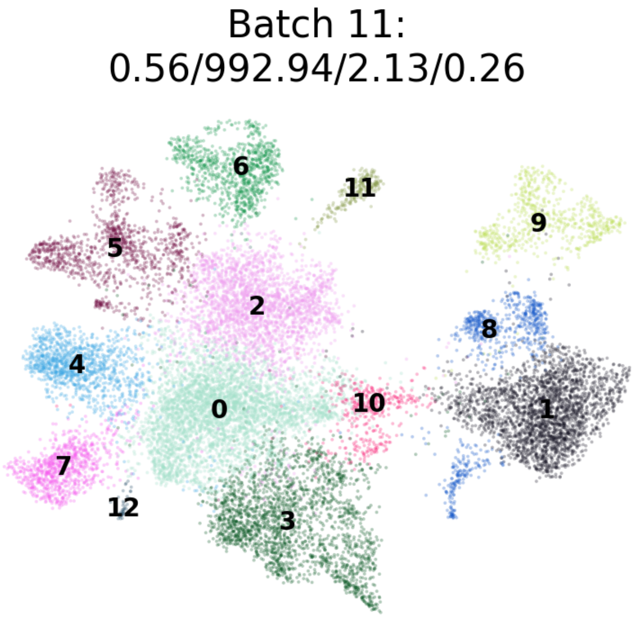

F

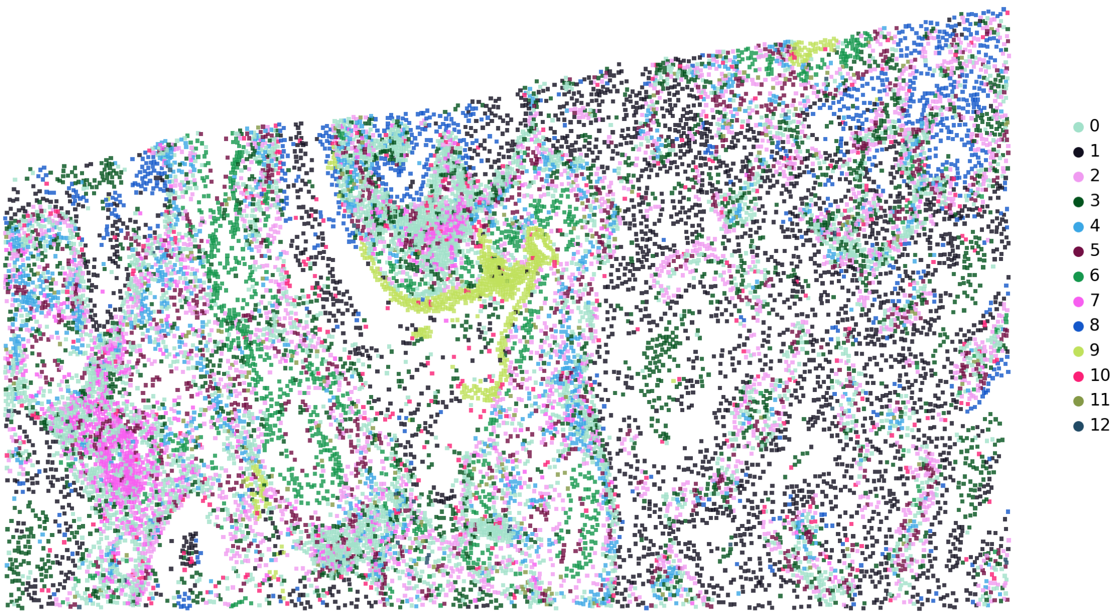

Supplementary Figure S3. Visualization of the SPICEiST clustering results compared to the SpaceFlow and GEX-based clustering in the Xenium lung cancer dataset with the v1 panel and 10x cell segmentation at a resolution of 0.6.

UMAP plots and the spatial distribution of cells illustrate the cell-level embedding and spatial distribution derived from SPICEiST-based analysis (SPICEiST; A: UMAP, B: spatial distribution of cells), SpaceFlow (SpaceFlow; C: UMAP, D: spatial distribution of cells) and

cell-level gene expression-based clustering (GEX; E: UMAP, F: spatial distribution of cells), respectively. The tissue sample, obtained from patch number 11 is presented, and the four numerical values at the top of the UMAP plot represent the ASW, CHI, DBI, and assortativity coefficient. Each dot in the plot corresponds to a cell, with color indicating the identity of the clusters.

A

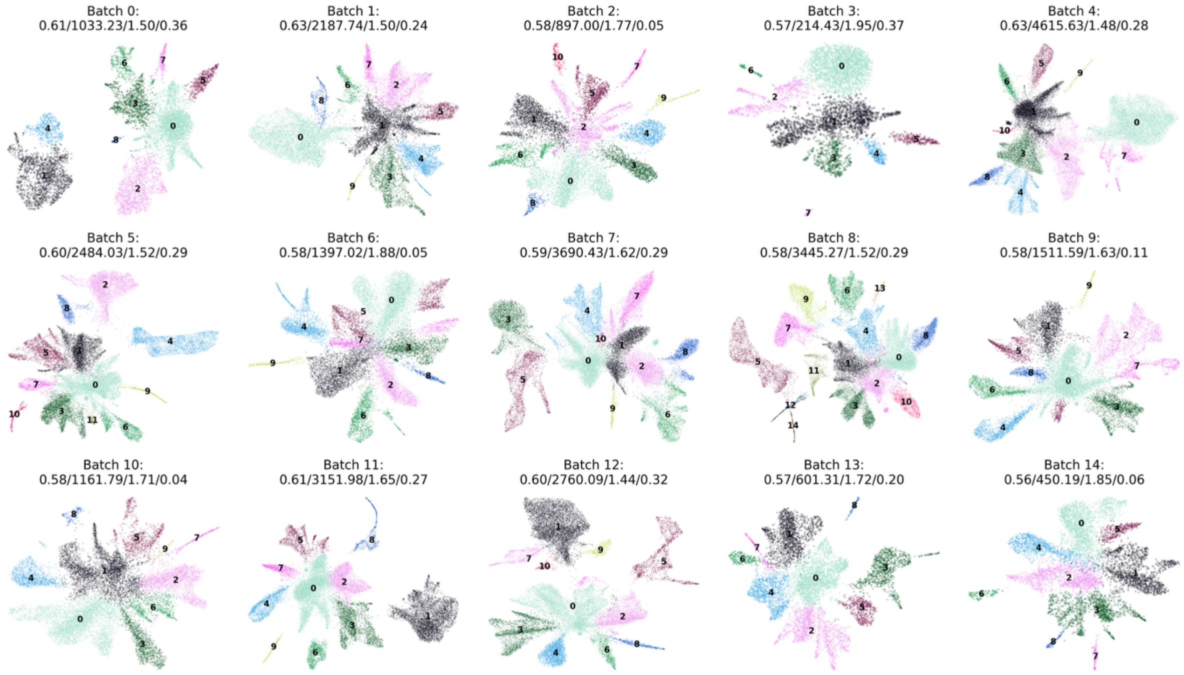
Batch 0:
0.61/1033.23/1.50/0.36
Batch 1:
0.63/2187.74/1.50/0.24
Batch 2:
0.58/897.00/1.77/0.05
Batch 3:
0.57/214.43/1.95/0.37
Batch 4:
0.63/4615.63/1.48/0.28
Batch 5:
0.60/2484.03/1.52/0.29
Batch 6:
0.58/1397.02/1.88/0.05
Batch 7:
0.59/3690.43/1.62/0.29
Batch 8:
0.58/3445.27/1.52/0.29
Batch 9:
0.58/1511.59/1.63/0.11
Batch 10:
0.58/1161.79/1.71/0.04
Batch 11:
0.61/3151.98/1.65/0.27
Batch 12:
0.60/2760.09/1.44/0.32
Batch 13:
0.57/601.31/1.72/0.20
Batch 14:
0.56/450.19/1.85/0.06

B

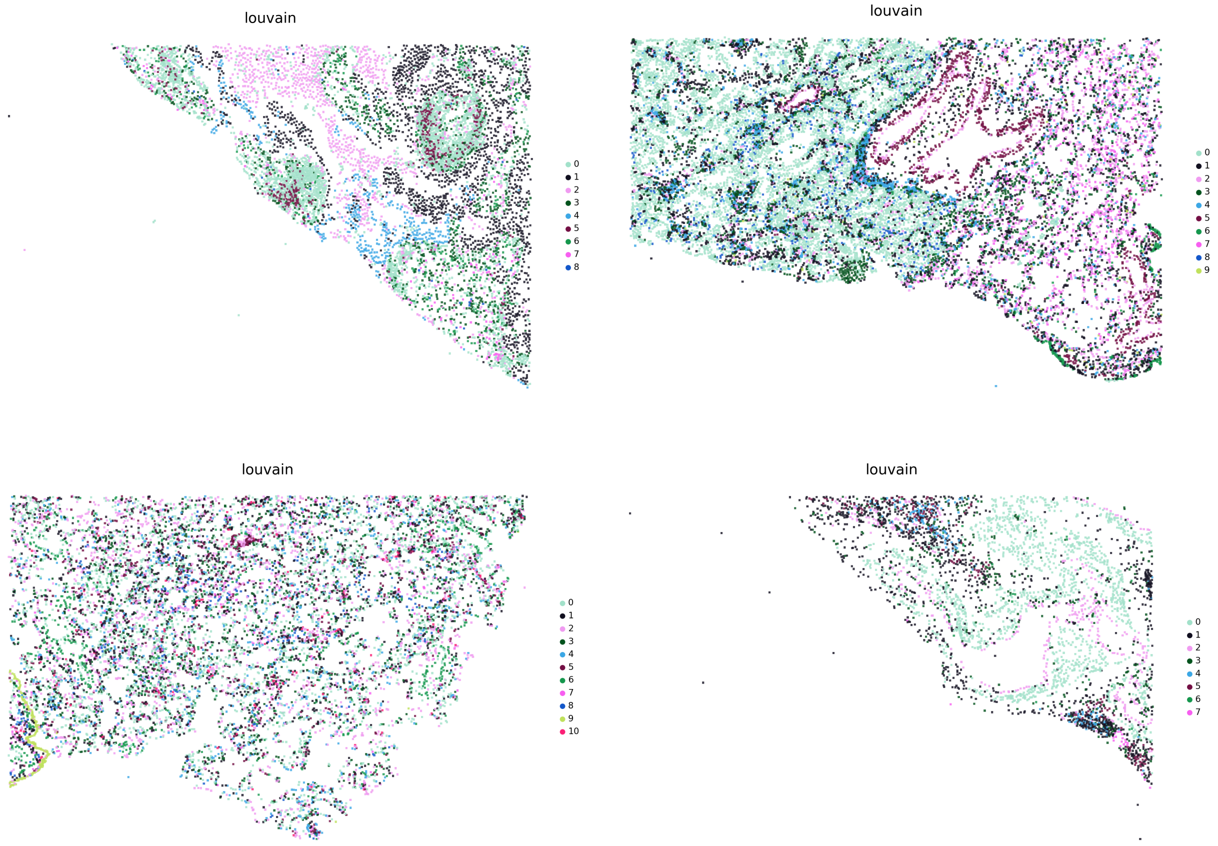
louvain
louvain
louvain
louvain

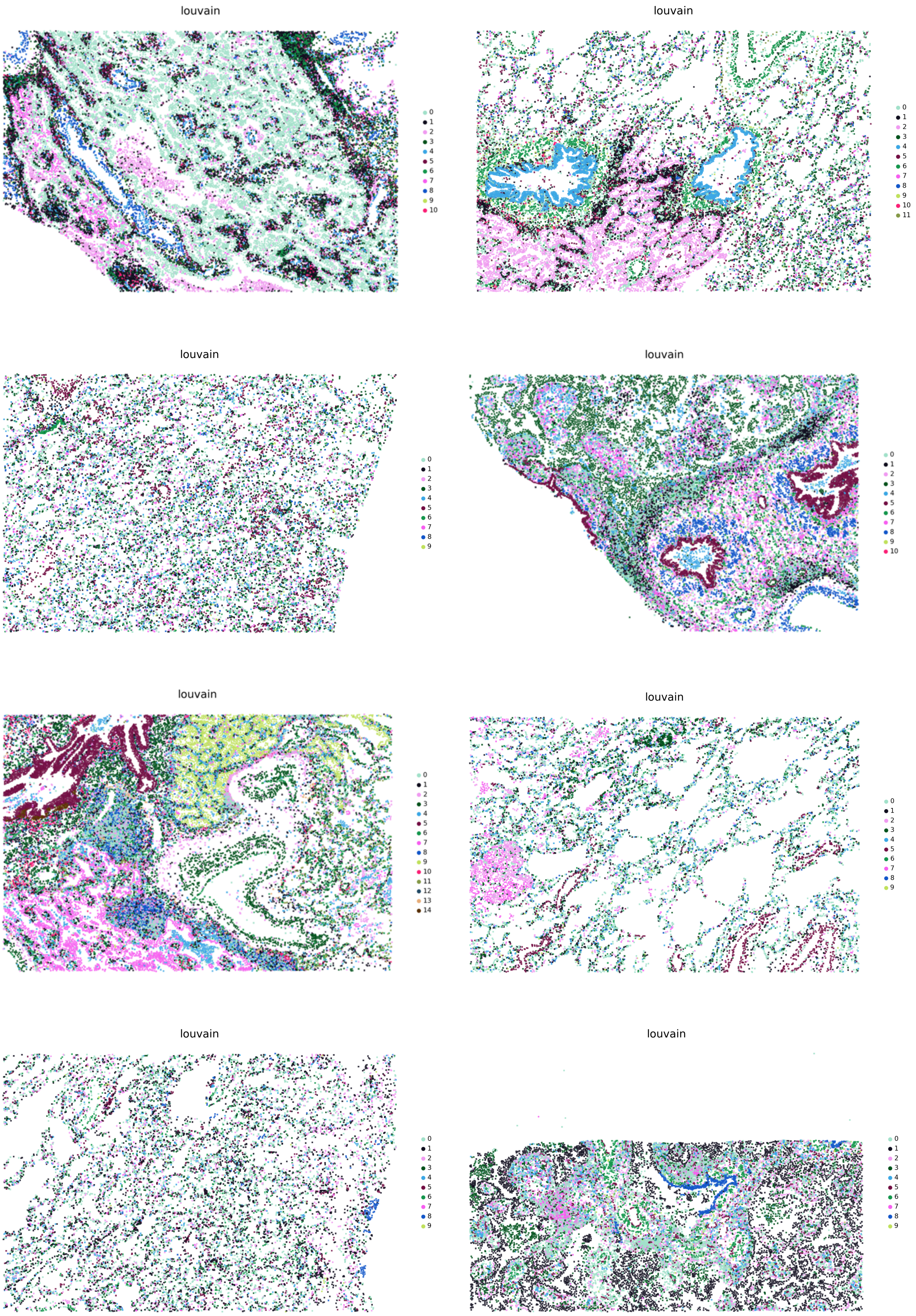
louvain
0 1 2 3 4 5 6 7 8 9 10
louvain
0 1 2 3 4 5 6 7 8 9 10 11
louvain
0 1 2 3 4 5 6 7 8 9
louvain
0 1 2 3 4 5 6 7 8 9 10
louvain
0 1 2 3 4 5 6 7 8 9 10 11 12 13 14
louvain
0 1 2 3 4 5 6 7 8 9
louvain
0 1 2 3 4 5 6 7 8 9
louvain
0 1 2 3 4 5 6 7 8 9

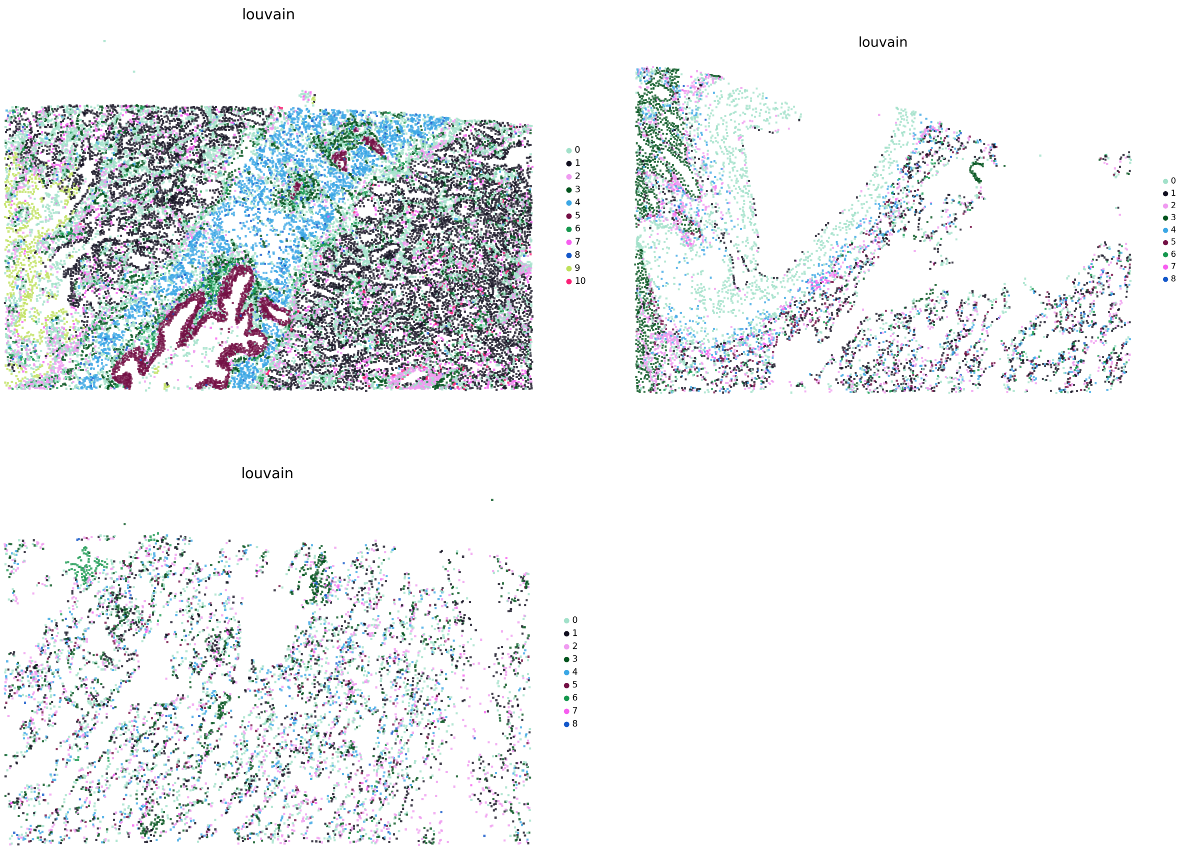
louvain
0
1
2
3
4
5
6
7
8
9
10
louvain
0
1
2
3
4
5
6
7
8
louvain
0
1
2
3
4
5
6
7
8

C

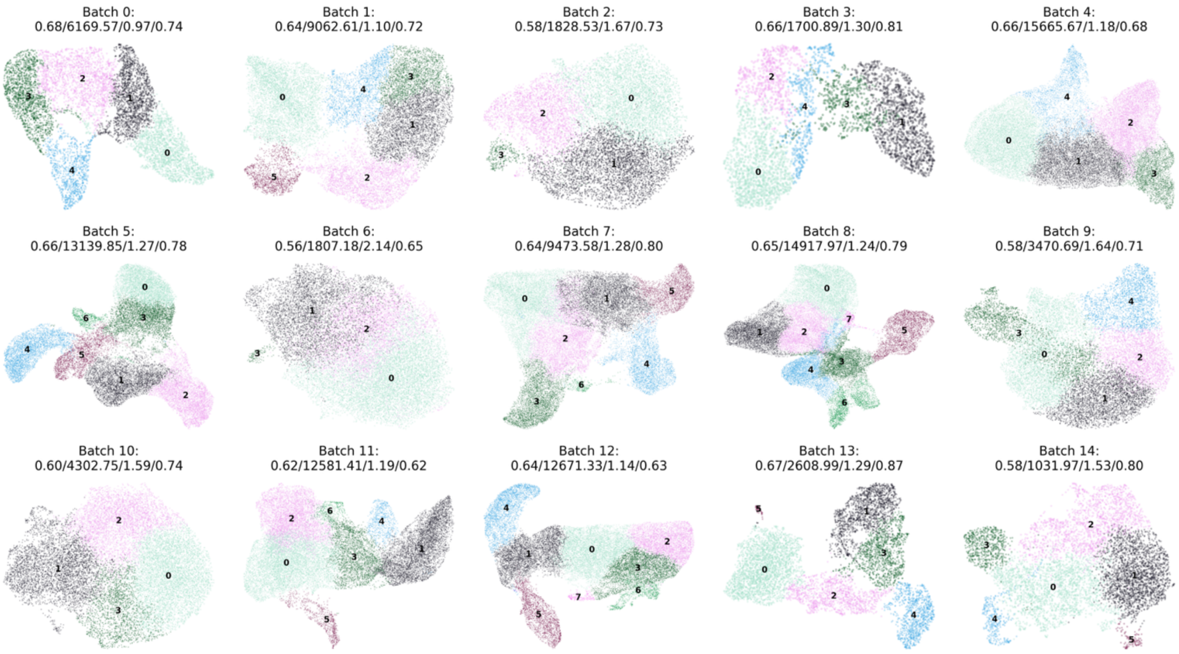
Batch 0:
0.68/6169.57/0.97/0.74
Batch 1:
0.64/9062.61/1.10/0.72
Batch 2:
0.58/1828.53/1.67/0.73
Batch 3:
0.66/1700.89/1.30/0.81
Batch 4:
0.66/15665.67/1.18/0.68
Batch 5:
0.66/13139.85/1.27/0.78
Batch 6:
0.56/1807.18/2.14/0.65
Batch 7:
0.64/9473.58/1.28/0.80
Batch 8:
0.65/14917.97/1.24/0.79
Batch 9:
0.58/3470.69/1.64/0.71
Batch 10:
0.60/4302.75/1.59/0.74
Batch 11:
0.62/12581.41/1.19/0.62
Batch 12:
0.64/12671.33/1.14/0.63
Batch 13:
0.67/2608.99/1.29/0.87
Batch 14:
0.58/1031.97/1.53/0.80

D

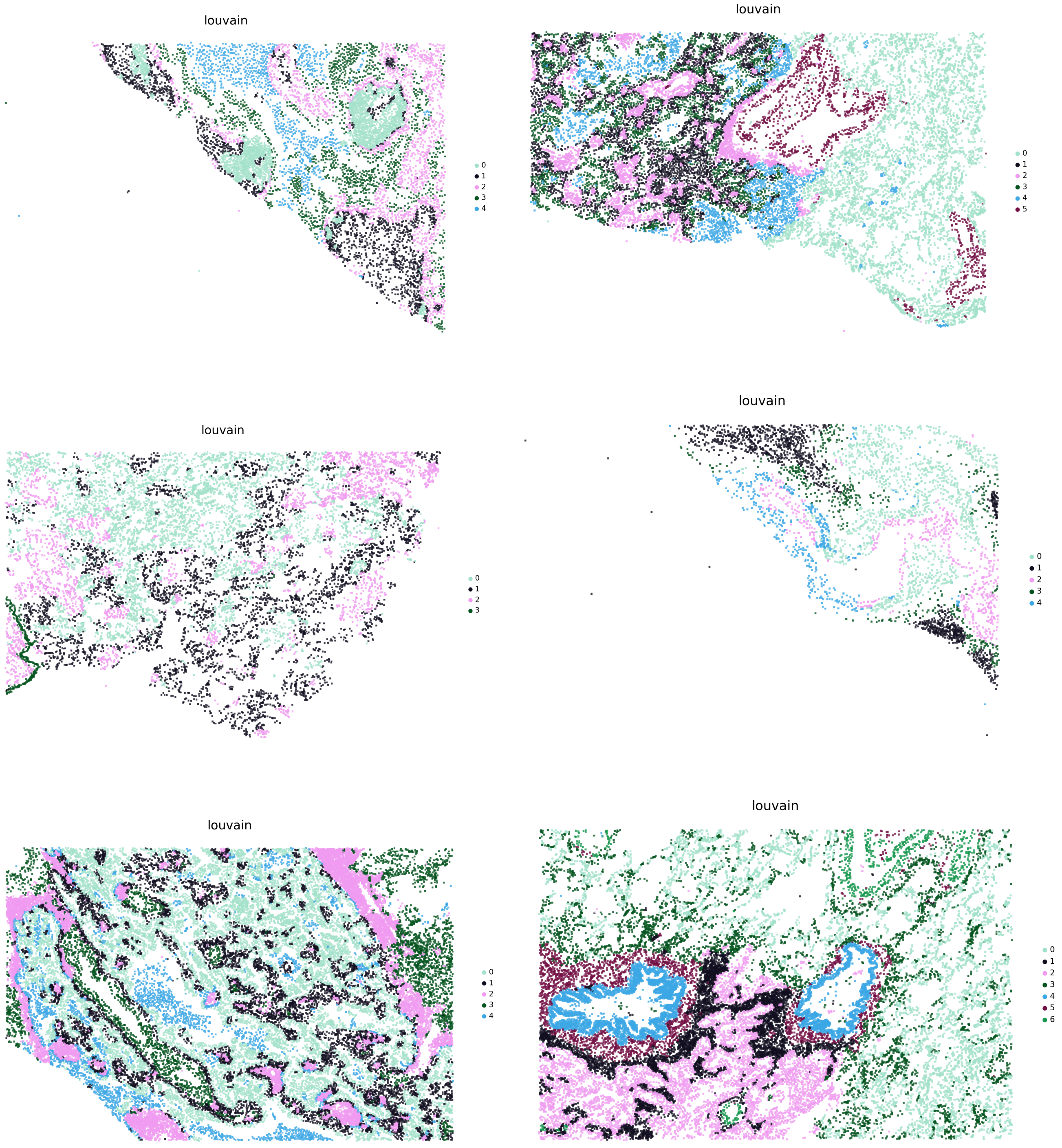
louvain
0
1
2
3
4
louvain
0
1
2
3
4
5
louvain
0
1
2
3
louvain
0
1
2
3
4
louvain
0
1
2
3
4
louvain
0
1
2
3
4
5
6

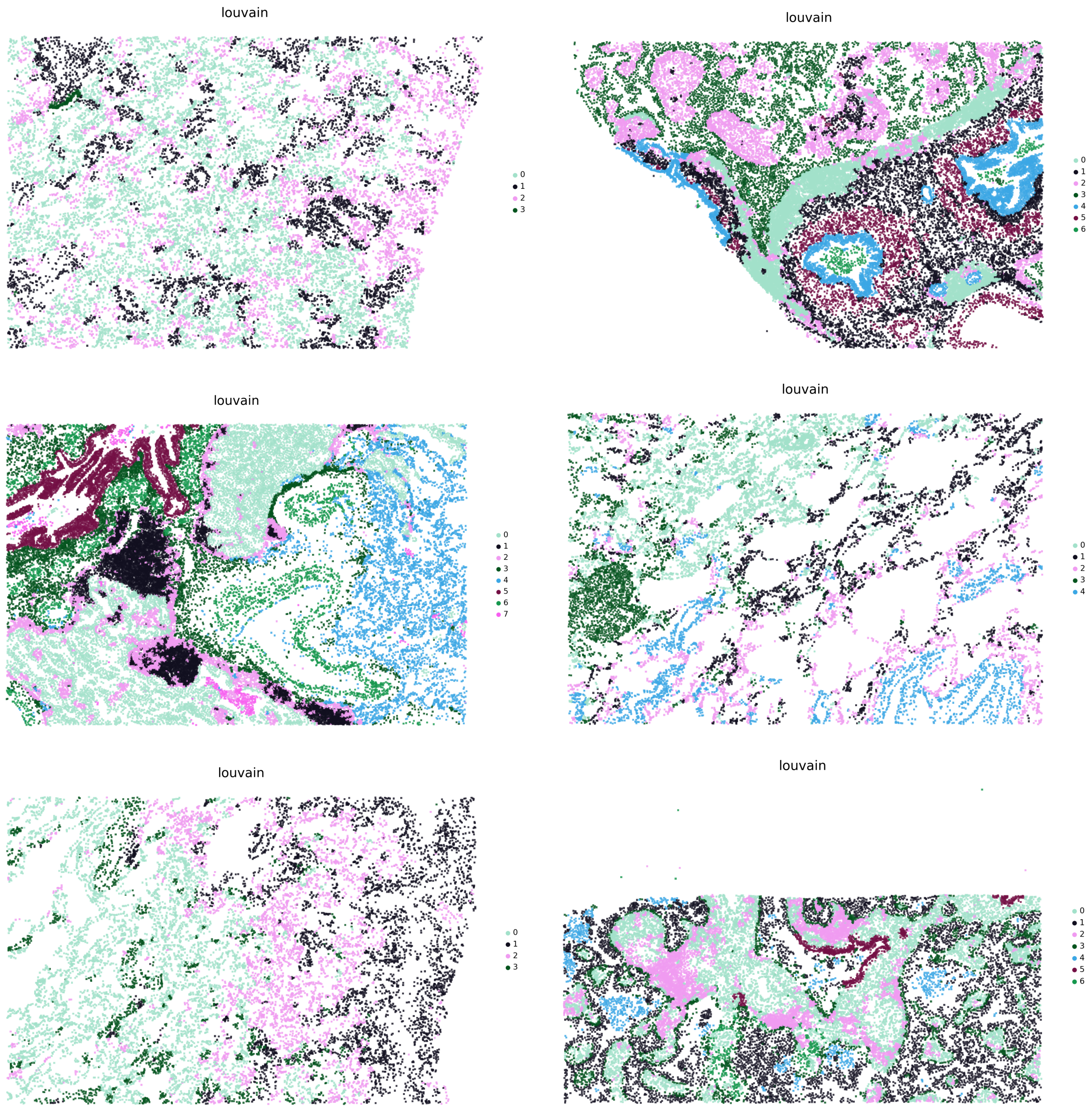
louvain
0
1
2
3
louvain
0
1
2
3
4
5
6
louvain
0
1
2
3
4
5
6
7
louvain
0
1
2
3
4
louvain
0
1
2
3
louvain
0
1
2
3
4
5
6

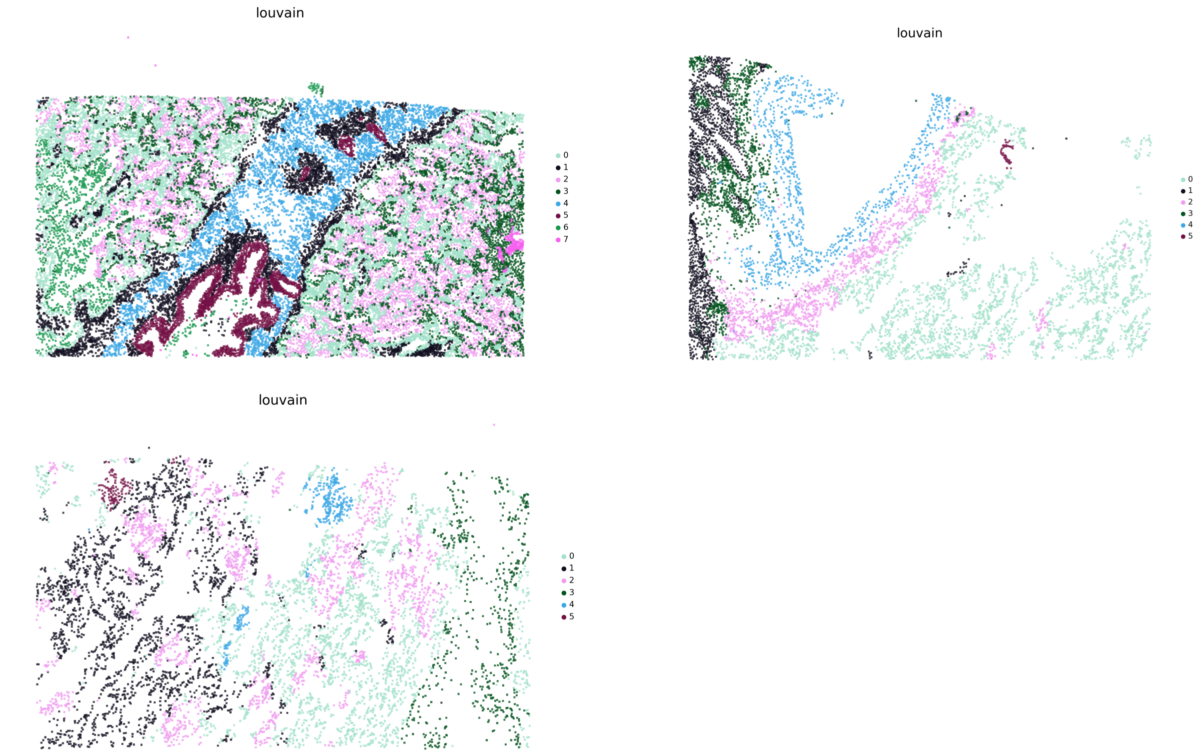
louvain
louvain
louvain

E

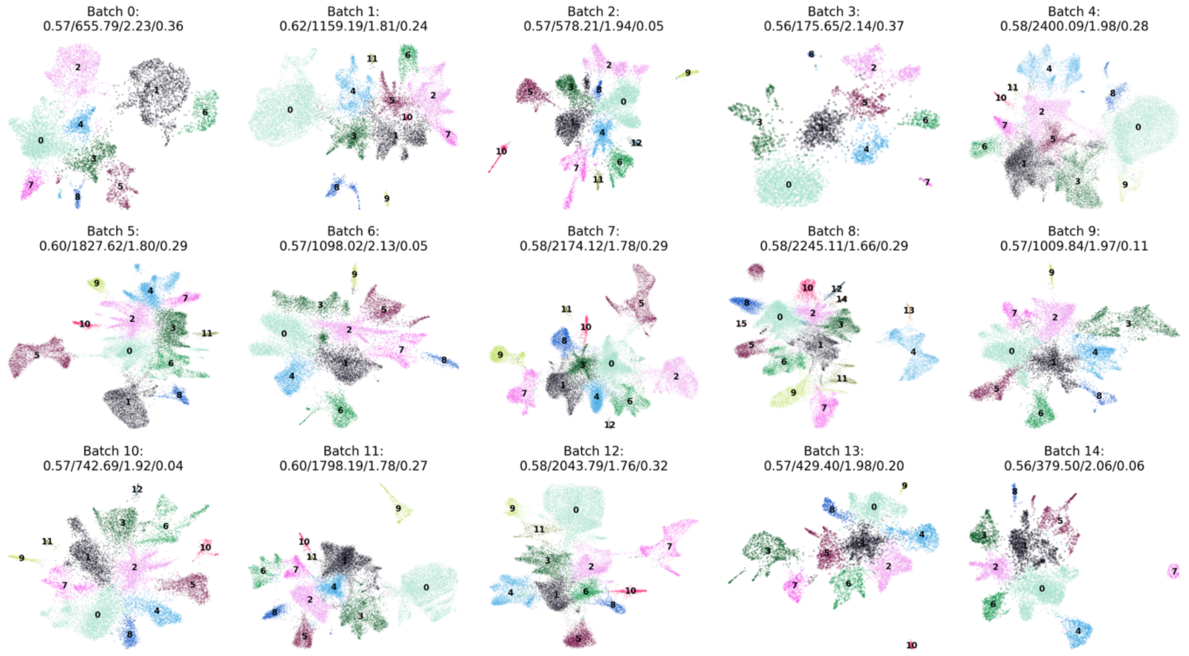
Batch 0:
0.57/655.79/2.23/0.36
Batch 1:
0.62/1159.19/1.81/0.24
Batch 2:
0.57/578.21/1.94/0.05
Batch 3:
0.56/175.65/2.14/0.37
Batch 4:
0.58/2400.09/1.98/0.28
Batch 5:
0.60/1827.62/1.80/0.29
Batch 6:
0.57/1098.02/2.13/0.05
Batch 7:
0.58/2174.12/1.78/0.29
Batch 8:
0.58/2245.11/1.66/0.29
Batch 9:
0.57/1009.84/1.97/0.11
Batch 10:
0.57/742.69/1.92/0.04
Batch 11:
0.60/1798.19/1.78/0.27
Batch 12:
0.58/2043.79/1.76/0.32
Batch 13:
0.57/429.40/1.98/0.20
Batch 14:
0.56/379.50/2.06/0.06

F

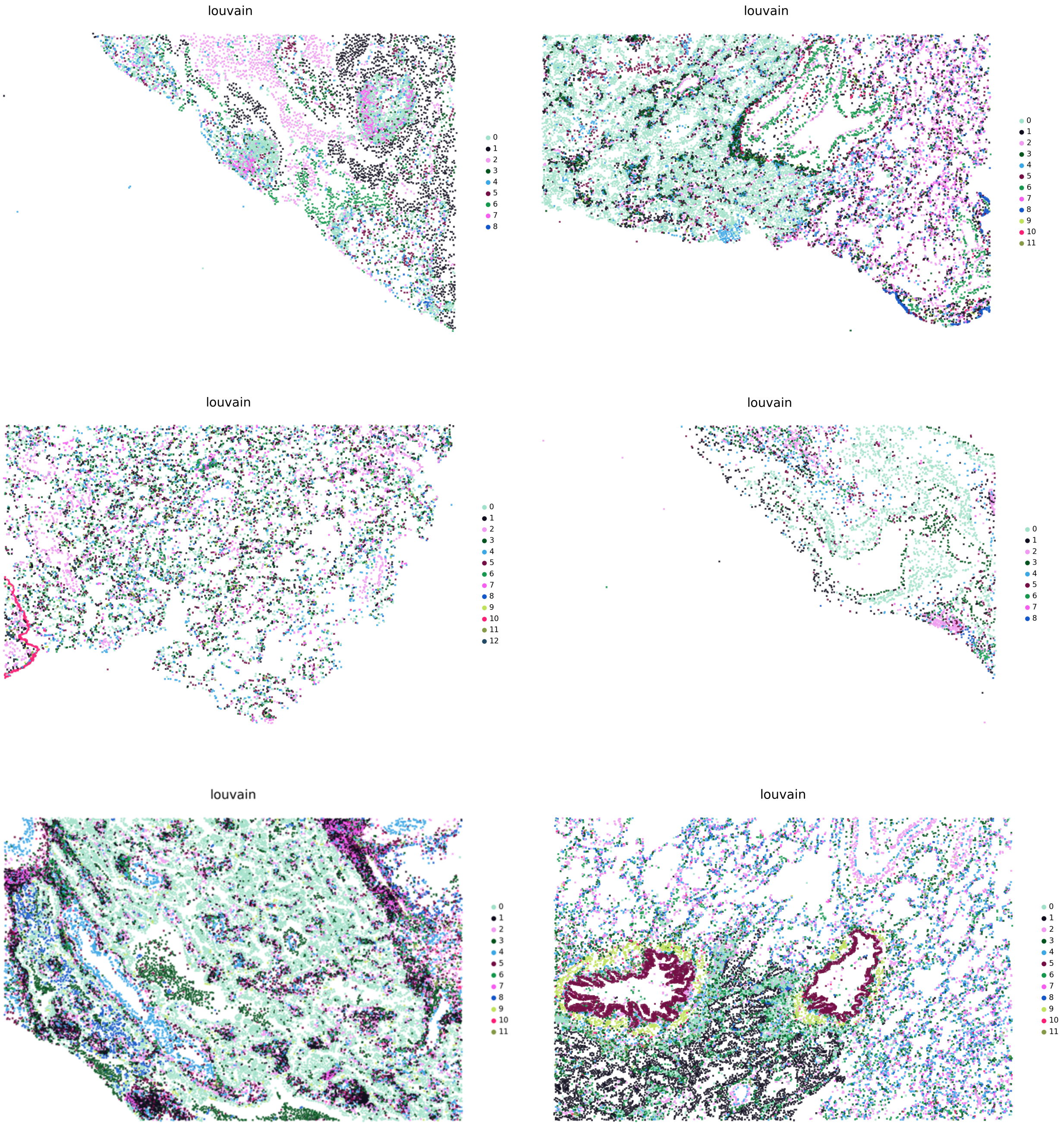
louvain
0
1
2
3
4
5
6
7
8
louvain
0
1
2
3
4
5
6
7
8
9
10
11
louvain
0
1
2
3
4
5
6
7
8
9
10
11
12
louvain
0
1
2
3
4
5
6
7
8
louvain
0
1
2
3
4
5
6
7
8
9
10
11
louvain
0
1
2
3
4
5
6
7
8
9
10
11

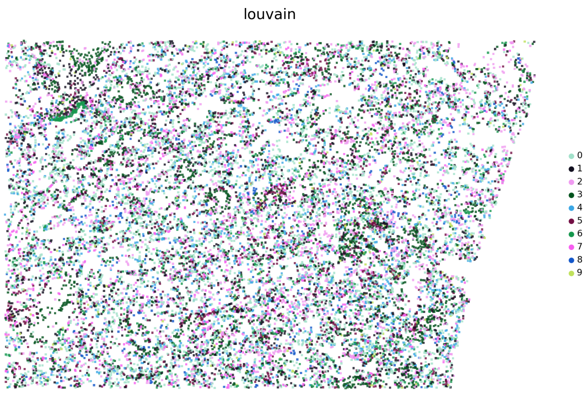
louvain
0
1
2
3
4
5
6
7
8
9

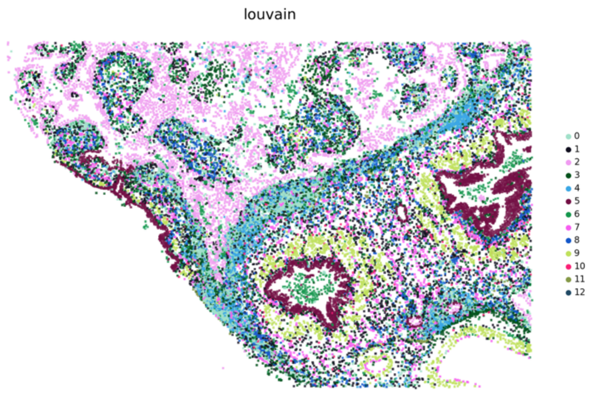
louvain
0
1
2
3
4
5
6
7
8
9
10
11
12

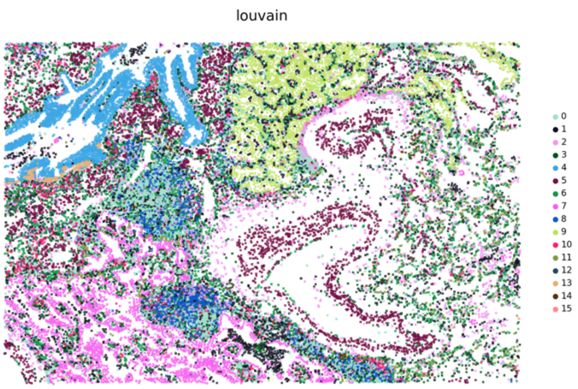
louvain
0
1
2
3
4
5
6
7
8
9
10
11
12
13
14
15

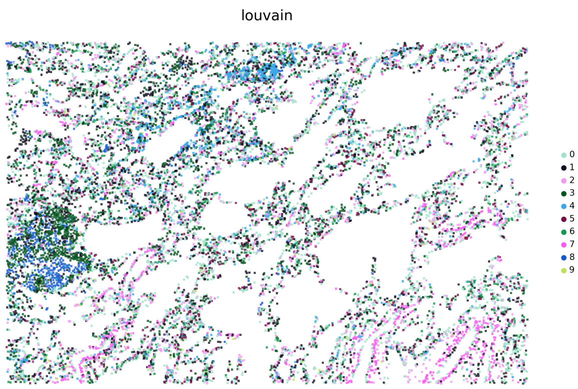
louvain
0
1
2
3
4
5
6
7
8
9

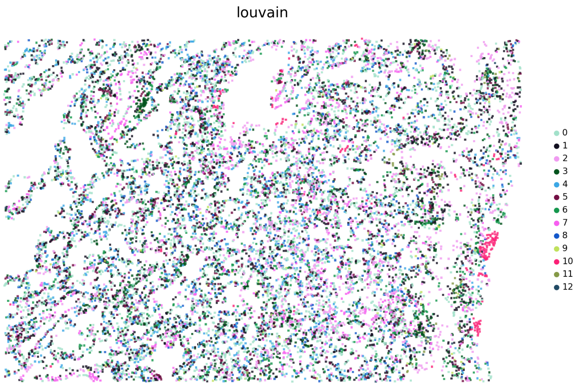
louvain
0
1
2
3
4
5
6
7
8
9
10
11
12

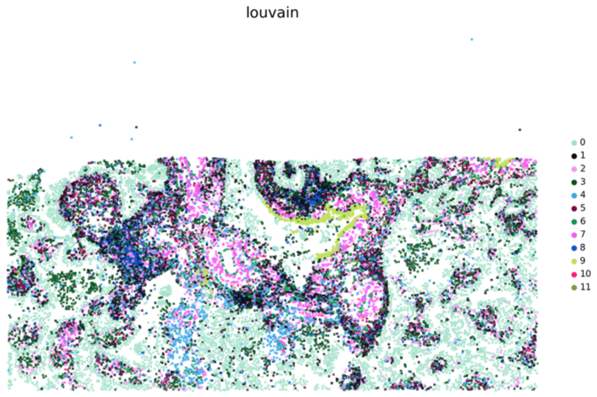
louvain
0
1
2
3
4
5
6
7
8
9
10
11

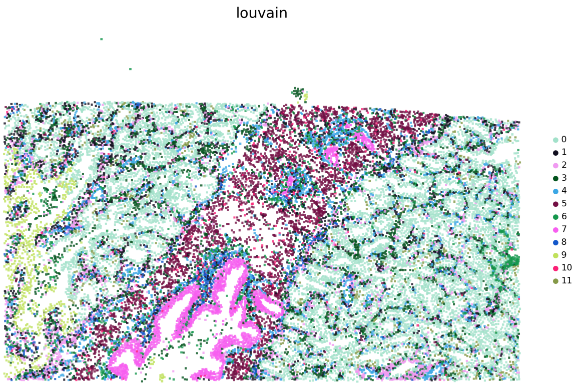
louvain
0
1
2
3
4
5
6
7
8
9
10
11

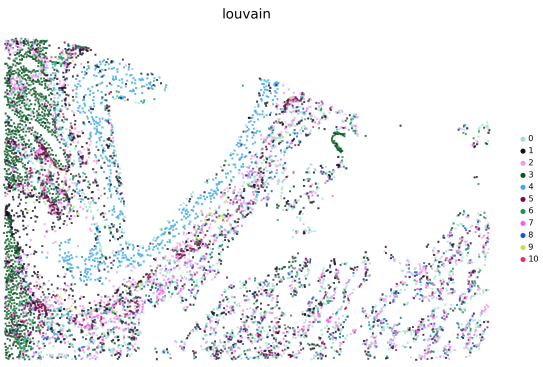
louvain
0
1
2
3
4
5
6
7
8
9
10

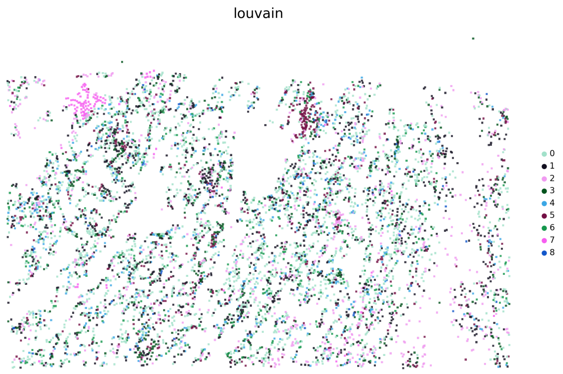

Supplementary Figure S4. Visualization of the SPICEiST clustering results compared to the GEX clustering in the Xenium lung cancer dataset with the 5K panel and 10x cell segmentation method at a resolution of 0.3.

UMAP plots and the spatial distribution of cells illustrate the cell-level embedding and spatial distribution derived from SPICEiST-based analysis (SPICEiST; A: UMAP, B: spatial distribution of cells), SpaceFlow (SpaceFlow; C: UMAP, D: spatial distribution of cells) and cell-level gene expression-based clustering (GEX; E: UMAP, F: spatial distribution of cells), respectively. The tissue samples, obtained from patch numbers 0 to 14, are presented serially from left to right and top to bottom. The four numerical values at the top of the UMAP plot represent the ASW, CHI, DBI, and assortativity coefficient. Each dot in the plot corresponds to a cell, with color indicating the identity of the clusters.

A

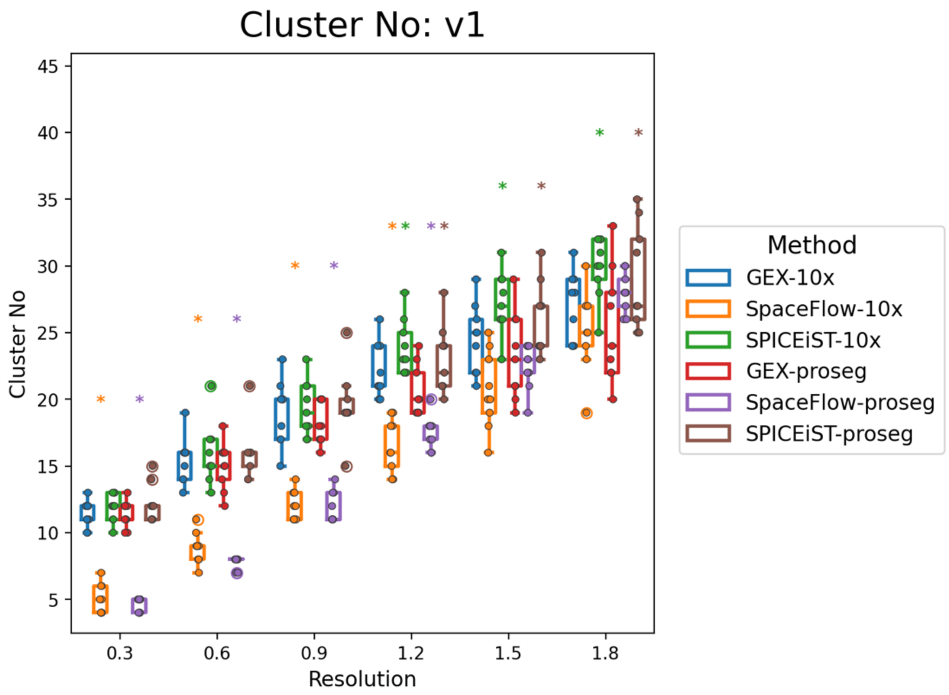

Cluster No: v1
Cluster No
Resolution
0.3
0.6
0.9
1.2
1.5
1.8
5
10
15
20
25
30
35
40
45
Method
GEX-10x
SpaceFlow-10x
SPICEiST-10x
GEX-proseg
SpaceFlow-proseg
SPICEiST-proseg

B

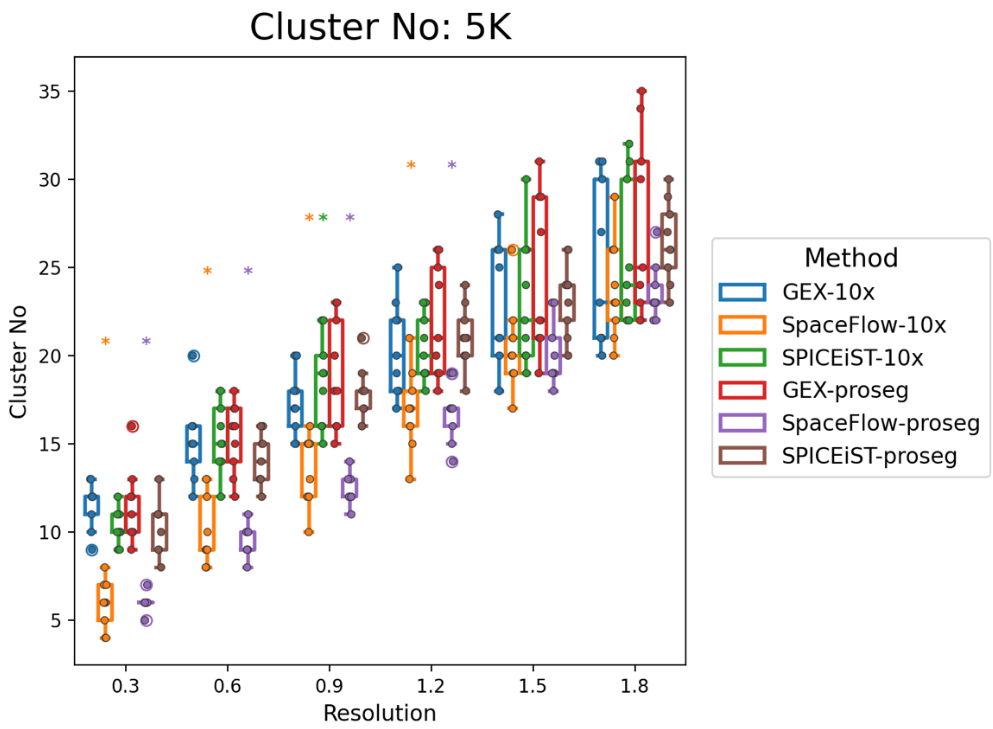

Cluster No: 5K
Cluster No
Resolution
0.3
0.6
0.9
1.2
1.5
1.8
5
10
15
20
25
30
35
Method
GEX-10x
SpaceFlow-10x
SPICEiST-10x
GEX-proseg
SpaceFlow-proseg
SPICEiST-proseg

Supplementary Figure S5. Comparison of the cluster number from SPICEiST, SpaceFlow, and GEX across different imaging-based ST platforms (v1 and Prime 5K) and cell segmentation methods in the Xenium human lung cancer dataset.

The boxplots compare cell cluster number, across varying resolutions of cell clusters in (A) Xenium v1 and (B) Prime 5K platforms. The alpha weight for the loss was set to 0.25 during training. The notation X-Y represents the cell clustering method X and the cell segmentation method Y. For example, SPICEiST-10x indicates that the SPICEiST clustering method was applied with the 10x cell segmentation. The stars above the boxplots show statistical comparisons (Wilcoxon signed-rank test) of GEX-10x with SpaceFlow-10x and SPICEiST-10x, and of GEX-proseg with SpaceFlow-proseg and SPICEiST-proseg. Multiple comparison correction was performed using the Benjamini-Hochberg method. *: $p \leq 0.05$, **: $p \leq 0.01$, ***: $p \leq 0.001$, ****: $p \leq 0.0001$.

A

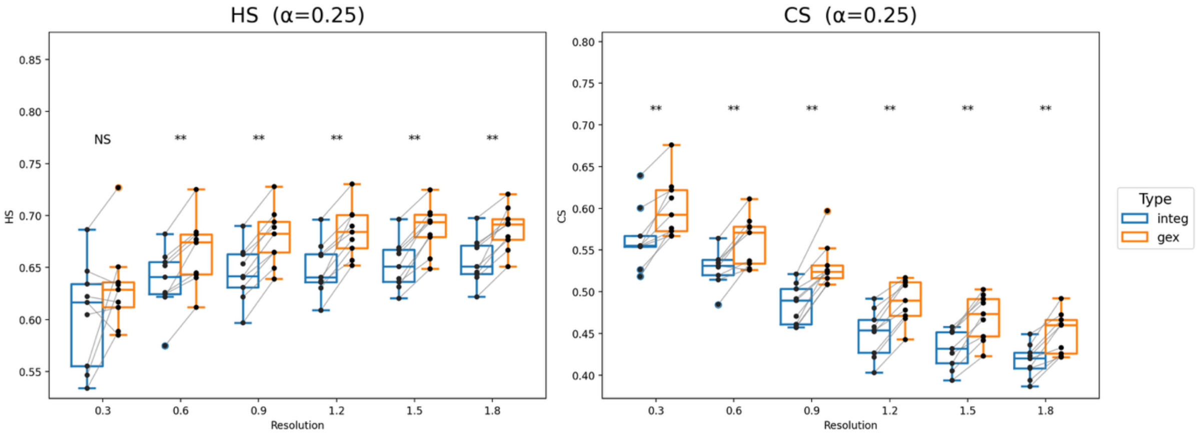

B

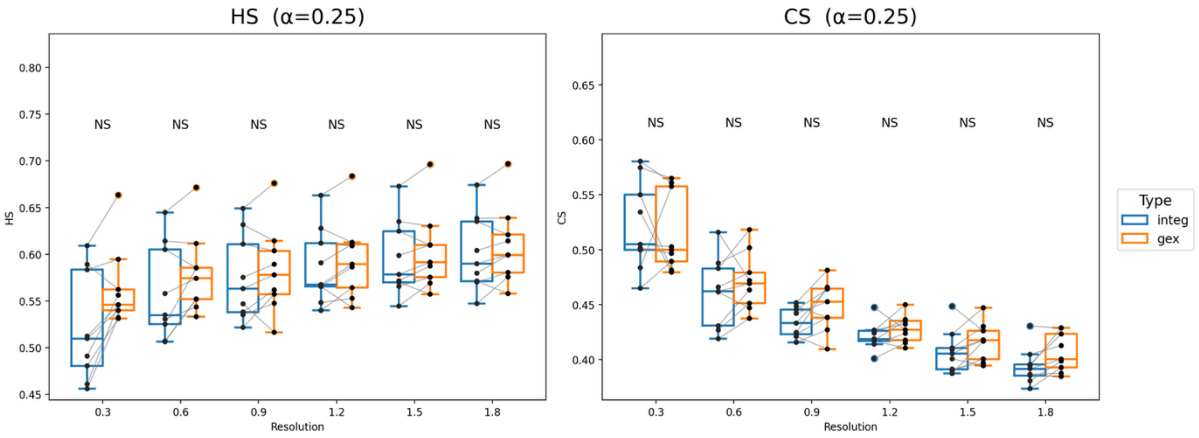

Supplementary Figure S6. Comparison of the homogeneity and completeness scores from SPICEiST and GEX across different imaging-based ST platforms (v1 and Prime 5K) in the Xenium human lung cancer dataset.

The boxplots compare homogeneity and completeness scores (HS and CS), across varying resolutions of cell clusters in (A) Xenium v1 and (B) Prime 5K platforms. The alpha weight for the loss was set to 0.25 during training. The stars above the boxplots show statistical comparisons (Wilcoxon signed-rank test) of GEX (annotated as "gex") with SPICEiST (annotated as "integ"). Multiple comparison correction was performed using the Benjamini-Hochberg method. NS: Not significant, *: $p \le 0.05$, **: $p \le 0.01$, ***: $p \le 0.001$, ****: $p \le 0.0001$.

A

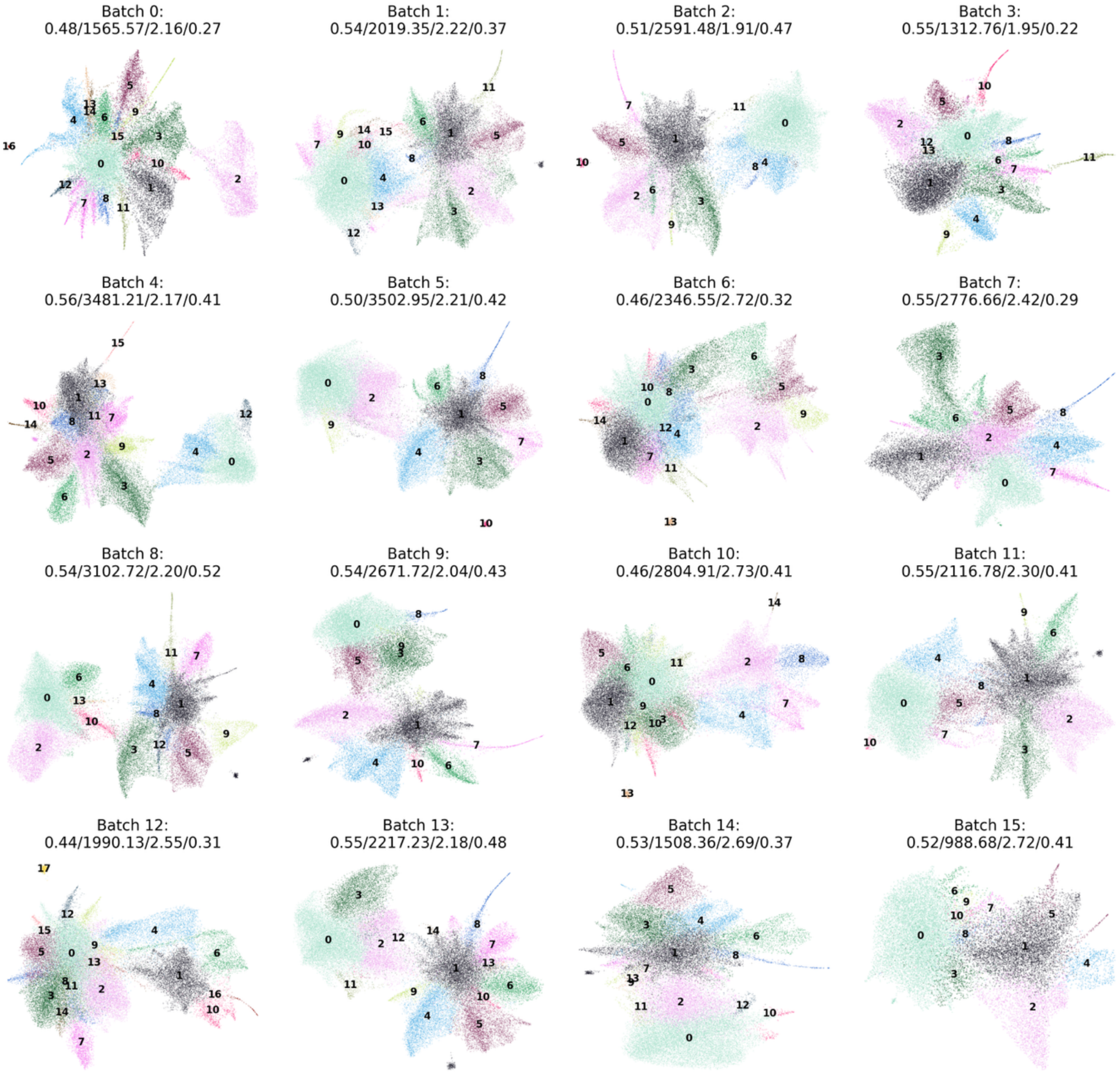
Batch 0:
0.48/1565.57/2.16/0.27
Batch 1:
0.54/2019.35/2.22/0.37
Batch 2:
0.51/2591.48/1.91/0.47
Batch 3:
0.55/1312.76/1.95/0.22
Batch 4:
0.56/3481.21/2.17/0.41
Batch 5:
0.50/3502.95/2.21/0.42
Batch 6:
0.46/2346.55/2.72/0.32
Batch 7:
0.55/2776.66/2.42/0.29
Batch 8:
0.54/3102.72/2.20/0.52
Batch 9:
0.54/2671.72/2.04/0.43
Batch 10:
0.46/2804.91/2.73/0.41
Batch 11:
0.55/2116.78/2.30/0.41
Batch 12:
0.44/1990.13/2.55/0.31
Batch 13:
0.55/2217.23/2.18/0.48
Batch 14:
0.53/1508.36/2.69/0.37
Batch 15:
0.52/988.68/2.72/0.41

B

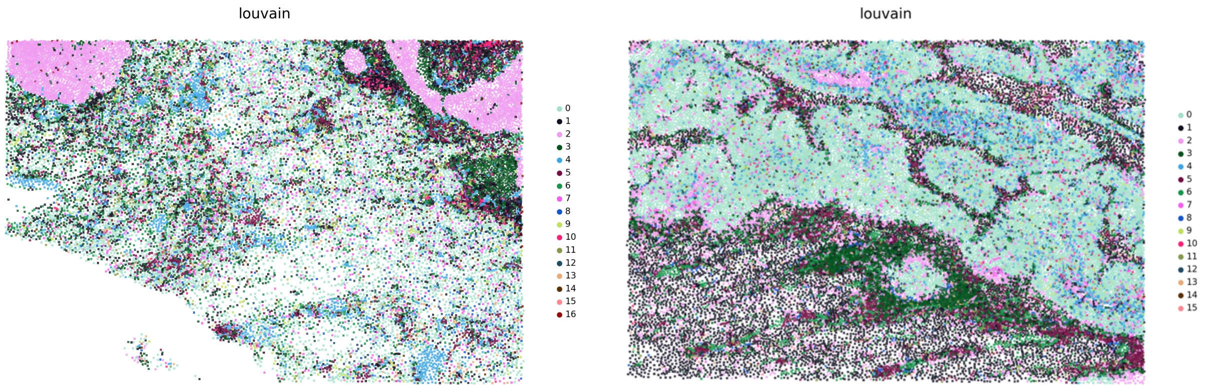
louvain
louvain

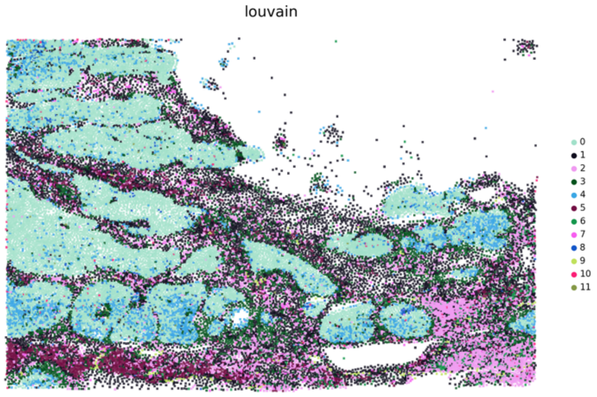
louvain
0
1
2
3
4
5
6
7
8
9
10
11

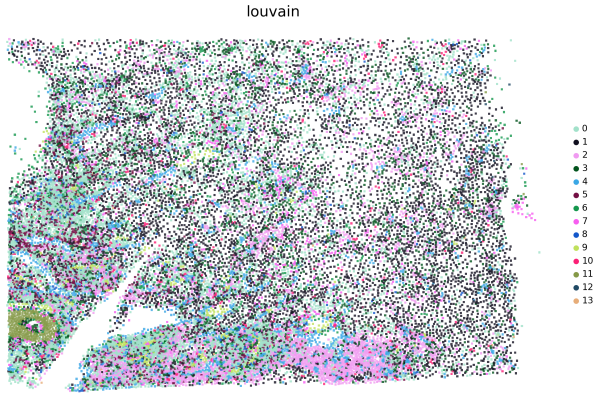
louvain
0
1
2
3
4
5
6
7
8
9
10
11
12
13

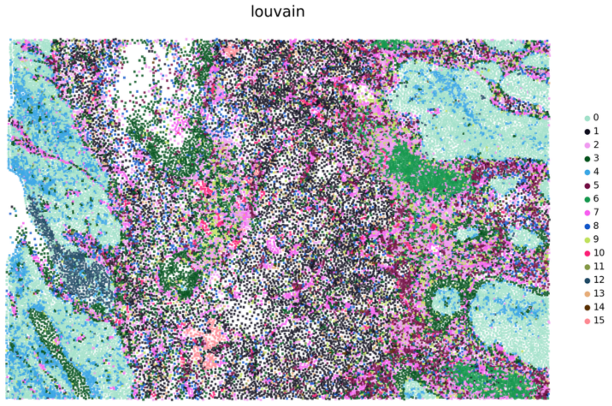
louvain
0
1
2
3
4
5
6
7
8
9
10
11
12
13
14
15

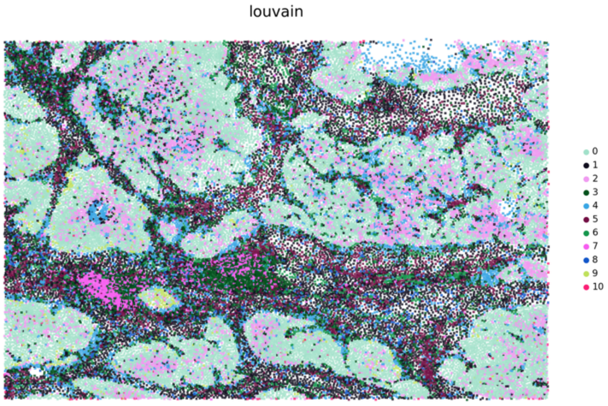
louvain
0
1
2
3
4
5
6
7
8
9
10

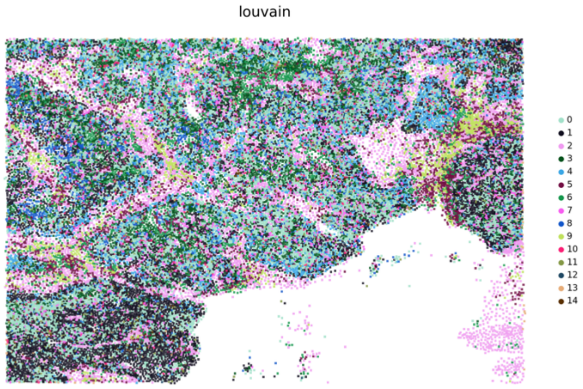
louvain
0
1
2
3
4
5
6
7
8
9
10
11
12
13
14

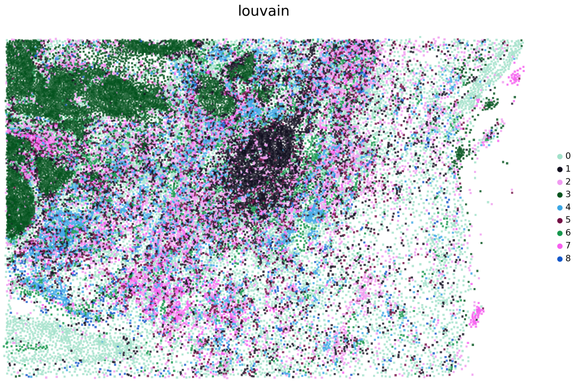
louvain
0
1
2
3
4
5
6
7
8

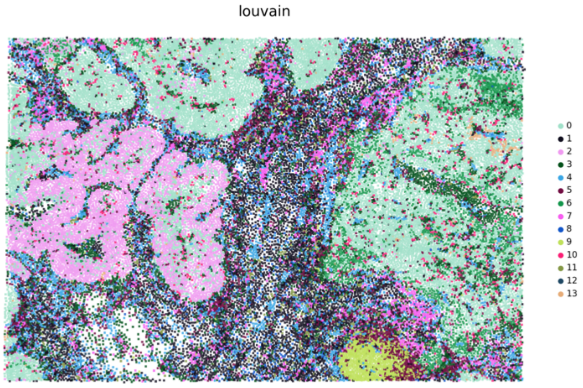
louvain
0
1
2
3
4
5
6
7
8
9
10
11
12
13

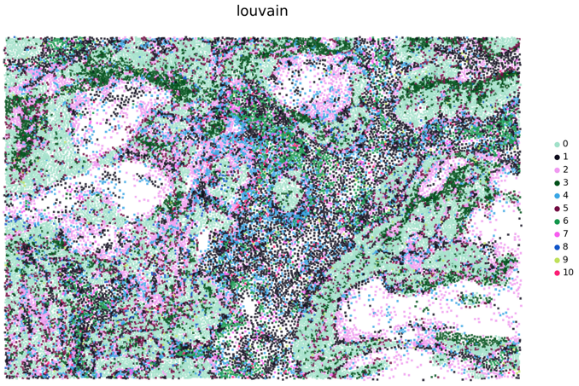
louvain
0
1
2
3
4
5
6
7
8
9
10

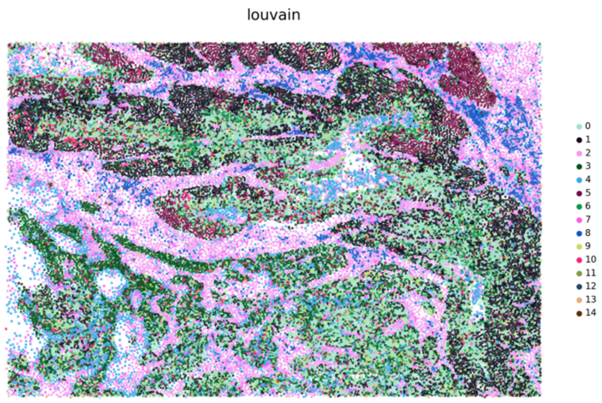
louvain
0
1
2
3
4
5
6
7
8
9
10
11
12
13
14

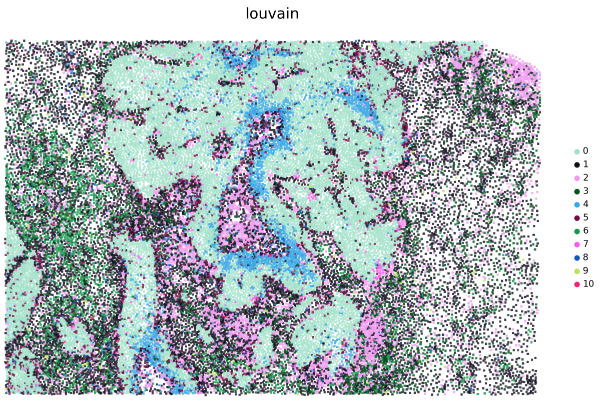
louvain
0
1
2
3
4
5
6
7
8
9
10

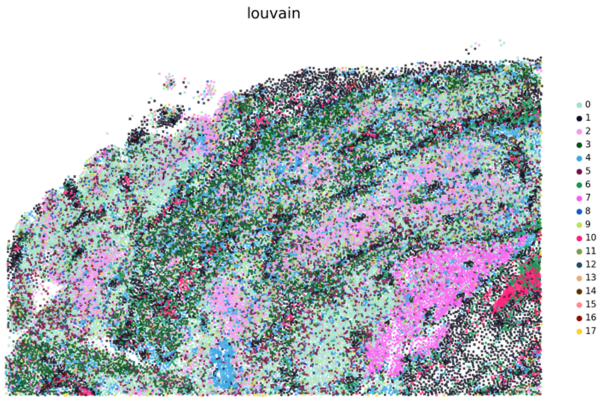
louvain
0
1
2
3
4
5
6
7
8
9
10
11
12
13
14
15
16
17

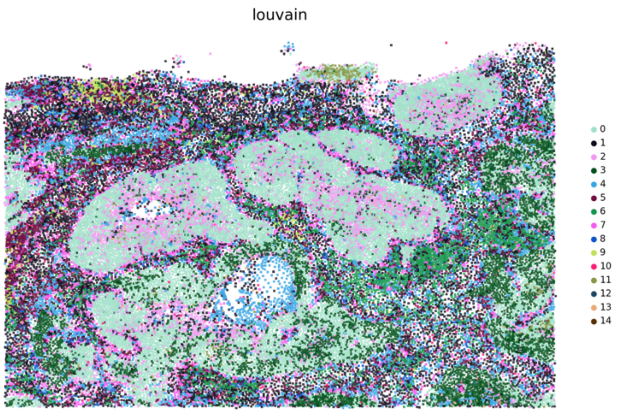
louvain
0
1
2
3
4
5
6
7
8
9
10
11
12
13
14

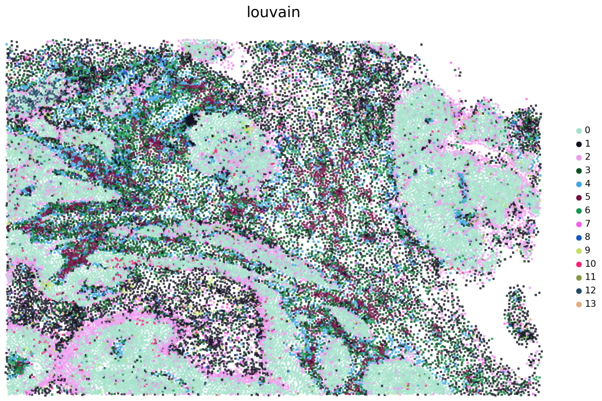
louvain
0
1
2
3
4
5
6
7
8
9
10
11
12
13

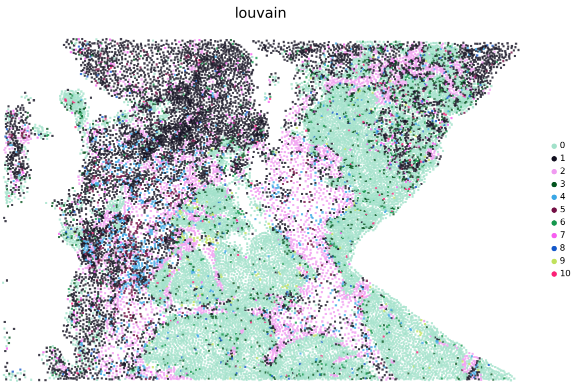
louvain
0
1
2
3
4
5
6
7
8
9
10

C

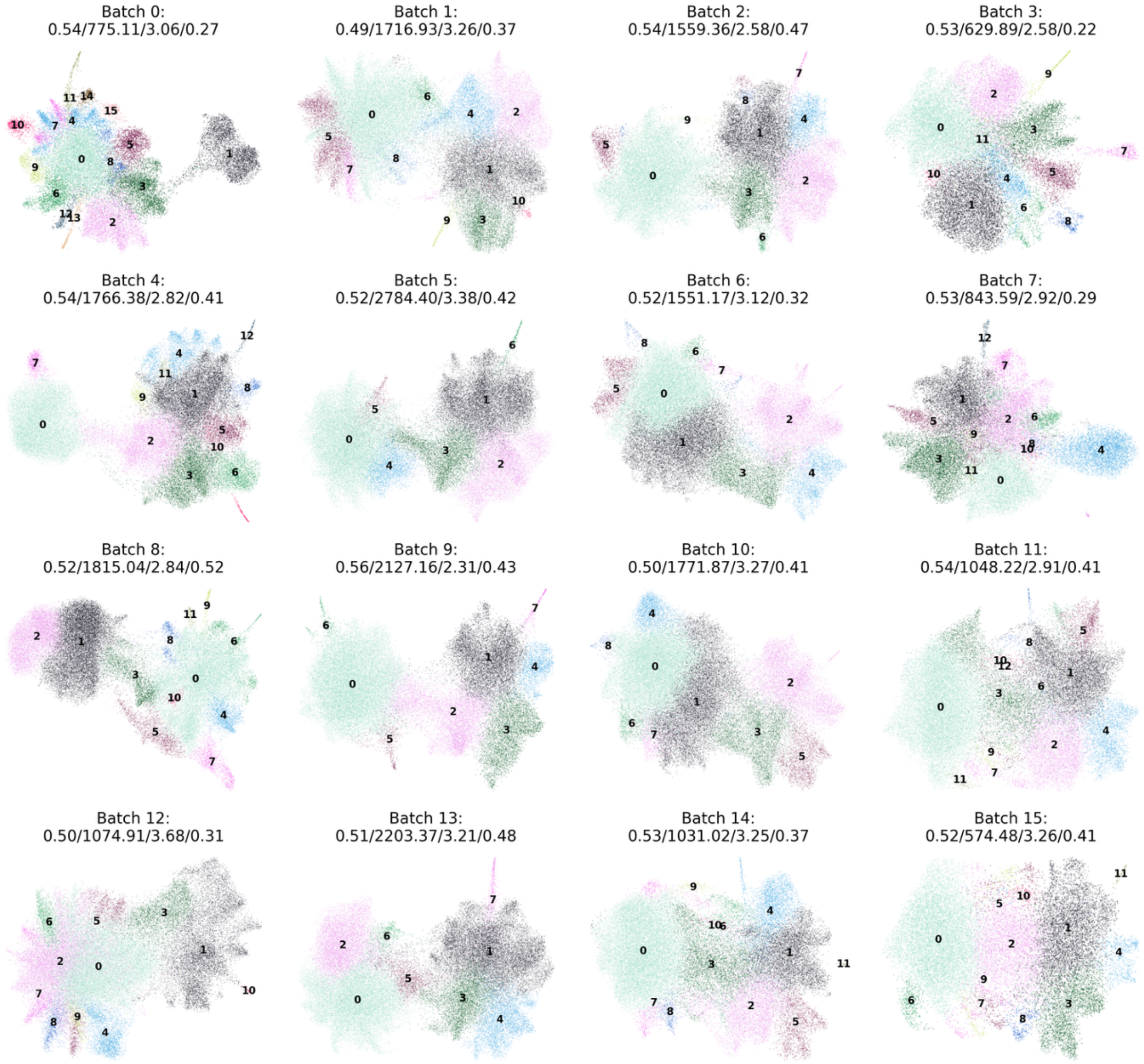
Batch 0:
0.54/775.11/3.06/0.27
Batch 1:
0.49/1716.93/3.26/0.37
Batch 2:
0.54/1559.36/2.58/0.47
Batch 3:
0.53/629.89/2.58/0.22
Batch 4:
0.54/1766.38/2.82/0.41
Batch 5:
0.52/2784.40/3.38/0.42
Batch 6:
0.52/1551.17/3.12/0.32
Batch 7:
0.53/843.59/2.92/0.29
Batch 8:
0.52/1815.04/2.84/0.52
Batch 9:
0.56/2127.16/2.31/0.43
Batch 10:
0.50/1771.87/3.27/0.41
Batch 11:
0.54/1048.22/2.91/0.41
Batch 12:
0.50/1074.91/3.68/0.31
Batch 13:
0.51/2203.37/3.21/0.48
Batch 14:
0.53/1031.02/3.25/0.37
Batch 15:
0.52/574.48/3.26/0.41

D

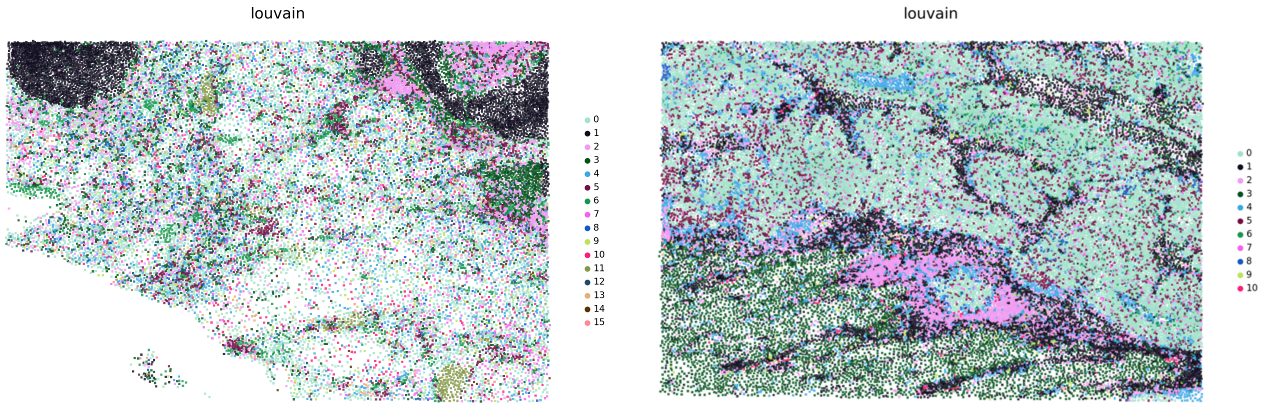
louvain
louvain
0
1
2
3
4
5
6
7
8
9
10
11
12
13
14
15

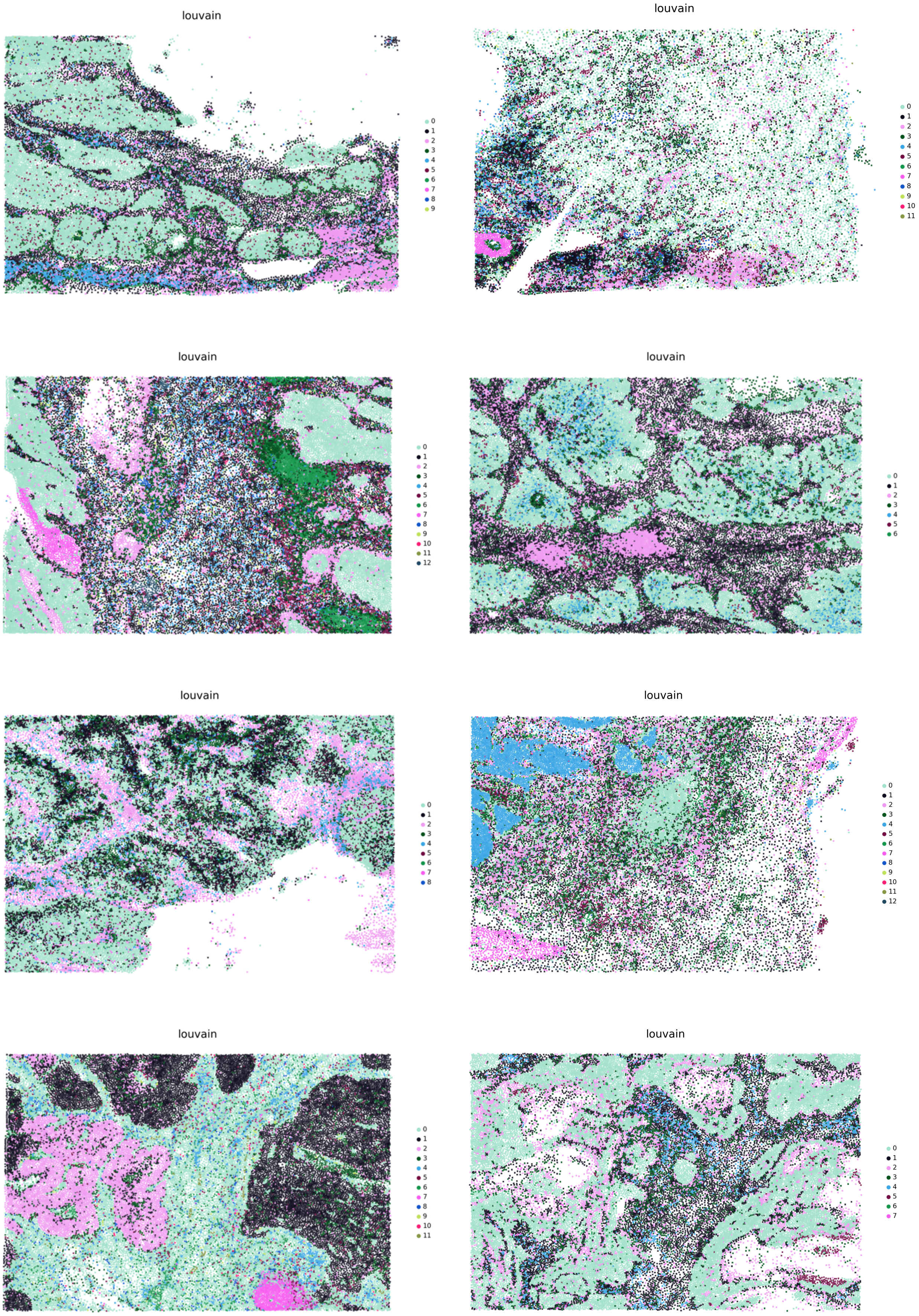
louvain
0 1 2 3 4 5 6 7 8 9
louvain
0 1 2 3 4 5 6 7 8 9 10 11
louvain
0 1 2 3 4 5 6 7 8 9 10 11 12
louvain
0 1 2 3 4 5 6
louvain
0 1 2 3 4 5 6 7 8
louvain
0 1 2 3 4 5 6 7 8 9 10 11 12
louvain
0 1 2 3 4 5 6 7 8 9 10 11
louvain
0 1 2 3 4 5 6 7

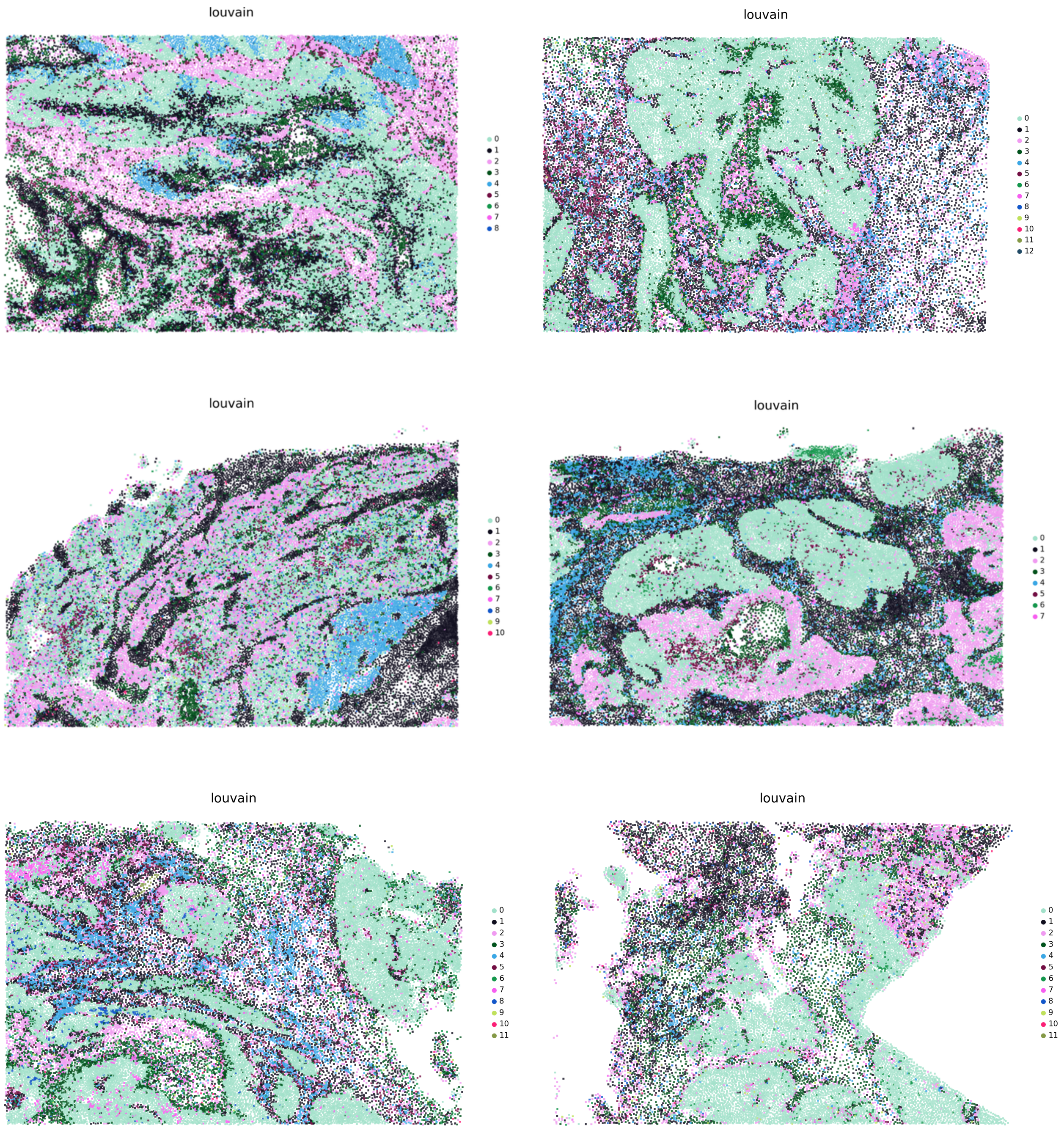

Supplementary Figure S7. Visualization of the SPICEiST clustering results compared to the GEX-based clustering in the Xenium colorectal cancer dataset with the v1 panel and 10x cell segmentation method at a resolution of 0.6.

UMAP plots and the spatial distribution of cells illustrate the cell-level embedding and spatial distribution derived from SPICEiST-based analysis (SPICEiST; A: UMAP, B: spatial distribution of cells) and cell-level gene expression-based clustering (GEX; C: UMAP, D: spatial distribution of cells), respectively. The tissue samples, obtained from patch numbers 0 to 15, are presented serially from left to right and top to bottom. The four numerical values at the top of the UMAP plot represent the ASW, CHI, DBI, and assortativity coefficient. Each dot in the plot corresponds to a cell, with color indicating the identity of the clusters.

A

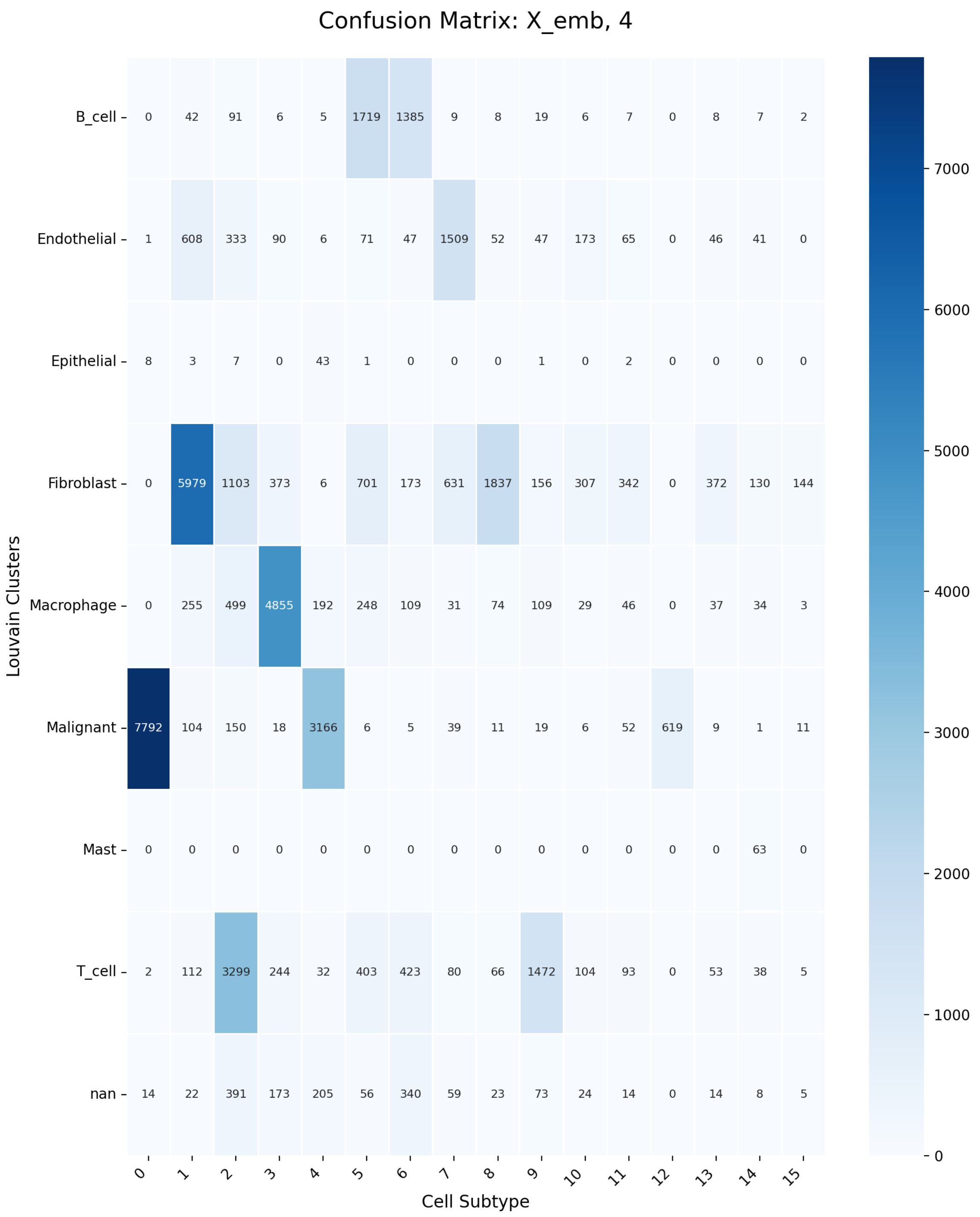
Confusion Matrix: X_emb, 4
Louvain Clusters
Cell Subtype
B_cell 0 42 91 6 5 1719 1385 9 8 19 6 7 0 8 7 2
Endothelial 1 608 333 90 6 71 47 1509 52 47 173 65 0 46 41 0
Epithelial 8 3 7 0 43 1 0 0 0 1 0 2 0 0 0 0
Fibroblast 0 5979 1103 373 6 701 173 631 1837 156 307 342 0 372 130 144
Macrophage 0 255 499 4855 192 248 109 31 74 109 29 46 0 37 34 3
Malignant 7792 104 150 18 3166 6 5 39 11 19 6 52 619 9 1 11
Mast 0 0 0 0 0 0 0 0 0 0 0 0 0 0 63 0
T_cell 2 112 3299 244 32 403 423 80 66 1472 104 93 0 53 38 5
nan 14 22 391 173 205 56 340 59 23 73 24 14 0 14 8 5
0 1 2 3 4 5 6 7 8 9 10 11 12 13 14 15
7000
6000
5000
4000
3000
2000
1000
0

B

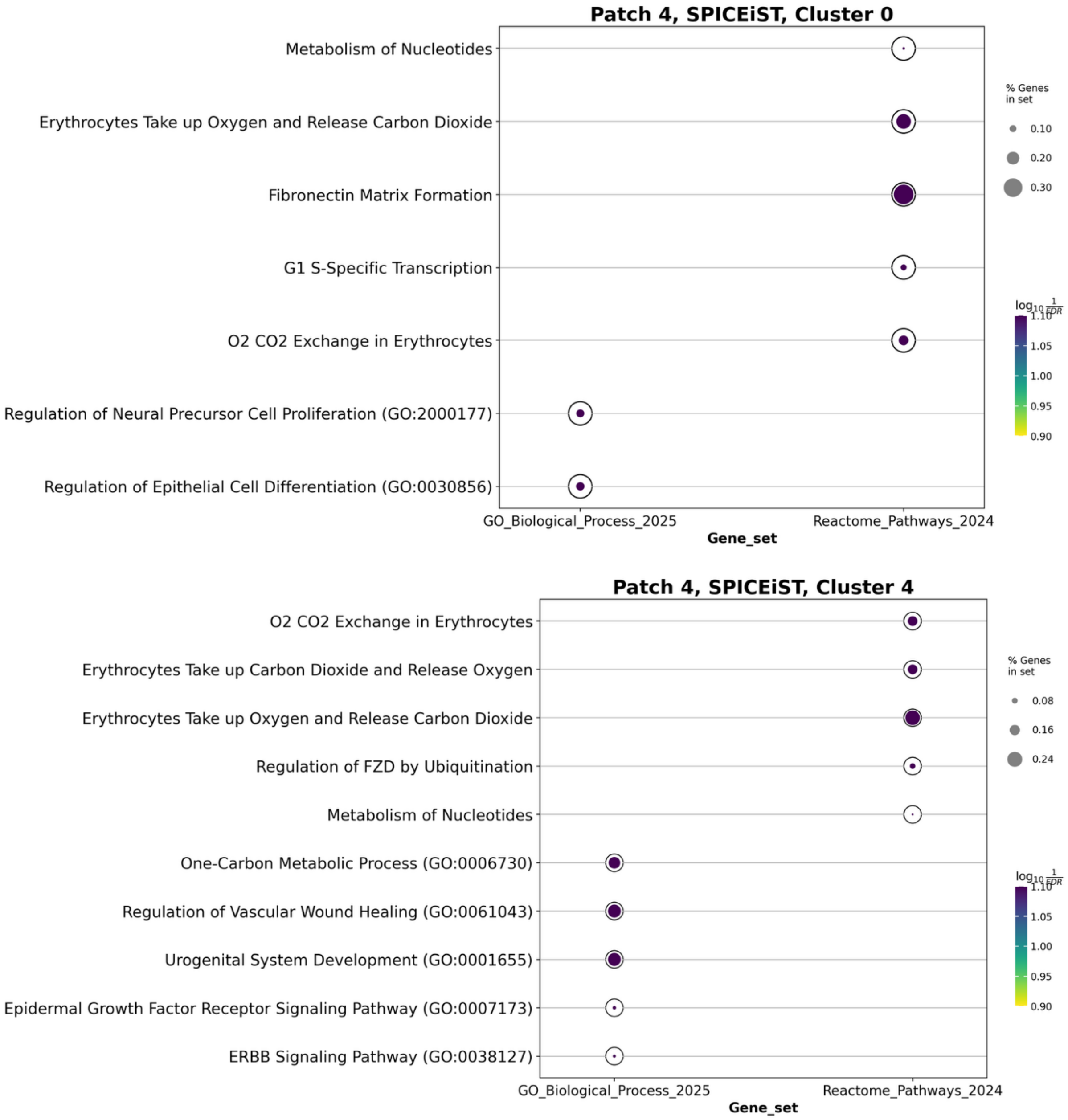
Patch 4, SPICEiST, Cluster 0
Metabolism of Nucleotides
Erythrocytes Take up Oxygen and Release Carbon Dioxide
Fibronectin Matrix Formation
G1 S-Specific Transcription
O2 CO2 Exchange in Erythrocytes
Regulation of Neural Precursor Cell Proliferation (GO:2000177)
Regulation of Epithelial Cell Differentiation (GO:0030856)
GO_Biological_Process_2025
Reactome_Pathways_2024
Gene_set
% Genes in set
0.10
0.20
0.30
log10 1/FDR
1.10
1.05
1.00
0.95
0.90
Patch 4, SPICEiST, Cluster 4
O2 CO2 Exchange in Erythrocytes
Erythrocytes Take up Carbon Dioxide and Release Oxygen
Erythrocytes Take up Oxygen and Release Carbon Dioxide
Regulation of FZD by Ubiquitination
Metabolism of Nucleotides
One-Carbon Metabolic Process (GO:0006730)
Regulation of Vascular Wound Healing (GO:0061043)
Urogenital System Development (GO:0001655)
Epidermal Growth Factor Receptor Signaling Pathway (GO:0007173)
ERBB Signaling Pathway (GO:0038127)
GO_Biological_Process_2025
Reactome_Pathways_2024
Gene_set
% Genes in set
0.08
0.16
0.24
log10 1/FDR
1.10
1.05
1.00
0.95
0.90

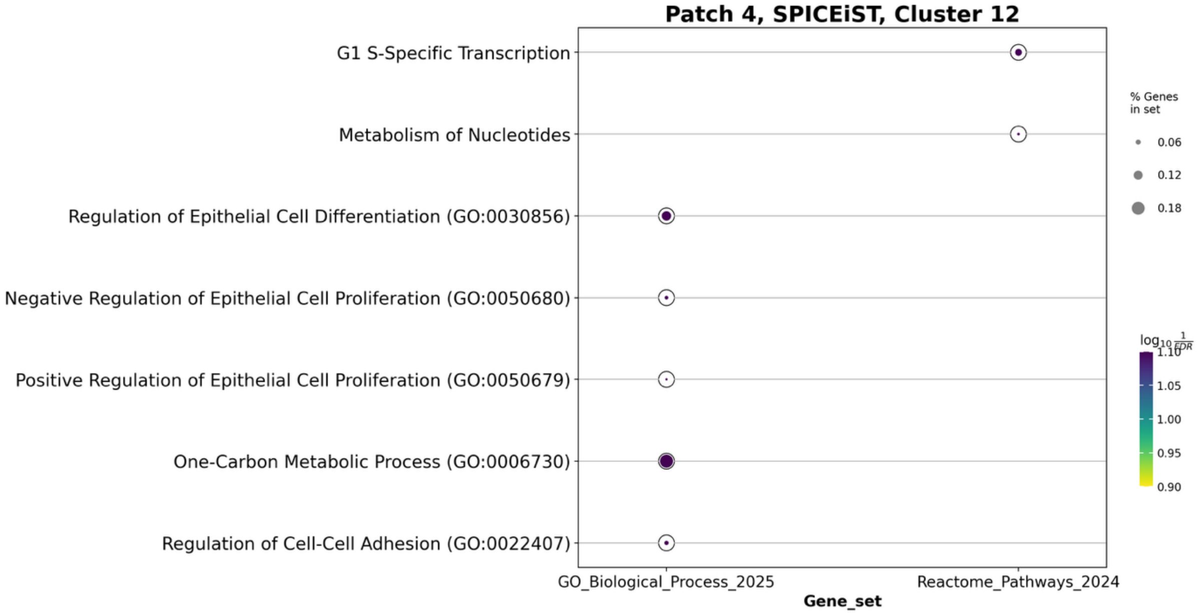

Supplementary Figure S8. Analysis of cell type correspondence and functional implications of malignant cell subtypes in patch number 4 of the Xenium colorectal cancer dataset.

(A) A confusion matrix showing the number of cells assigned to annotated cell types versus cell clusters derived from SPICEiST. The resolution of the cell clusters is 0.6. (B) The dot plot represents the Gene Ontology (GO) and Reactome enrichment analysis results. The analysis was performed for marker genes of clusters 0, 4, and 12, which have the highest proportion of malignant cells. The size of the dot represents percentage of marker genes that overlap with functional terms, and the color of the dot represents the statistical significance on -log scale.

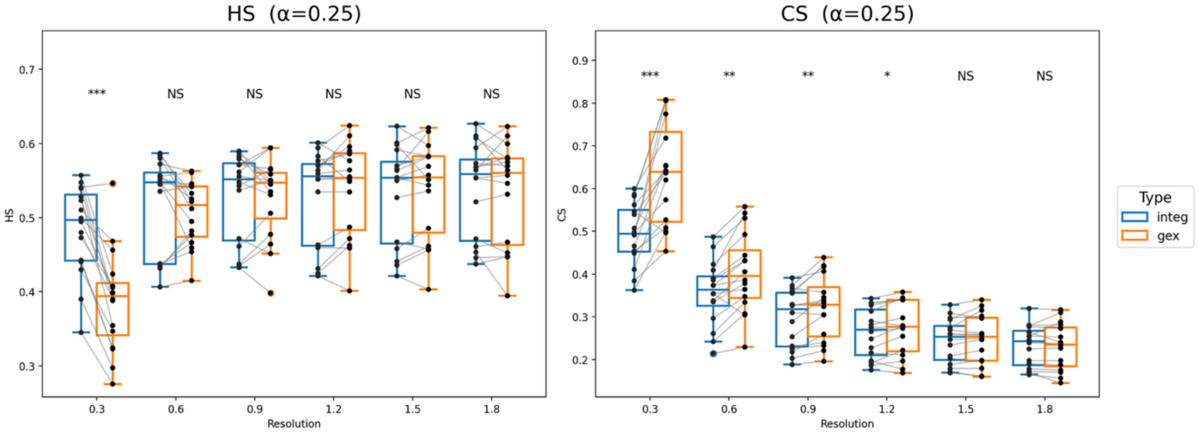

Supplementary Figure S9. Comparison of the homogeneity and completeness scores from SPICEiST and GEX in the Xenium colorectal cancer dataset.

The boxplots compare homogeneity and completeness scores (HS and CS), across varying resolutions of cell clusters. The alpha weight for the loss was set to 0.25 during training. The stars above the boxplots show statistical comparisons (Wilcoxon signed-rank test) of GEX (annotated as "gex") with SPICEiST (annotated as "integ"). Multiple comparison correction was performed using the Benjamini-Hochberg method. NS: Not significant, *: $p \le 0.05$, **: $p \le 0.01$, ***: $p \le 0.001$, ****: $p \le 0.0001$.

A

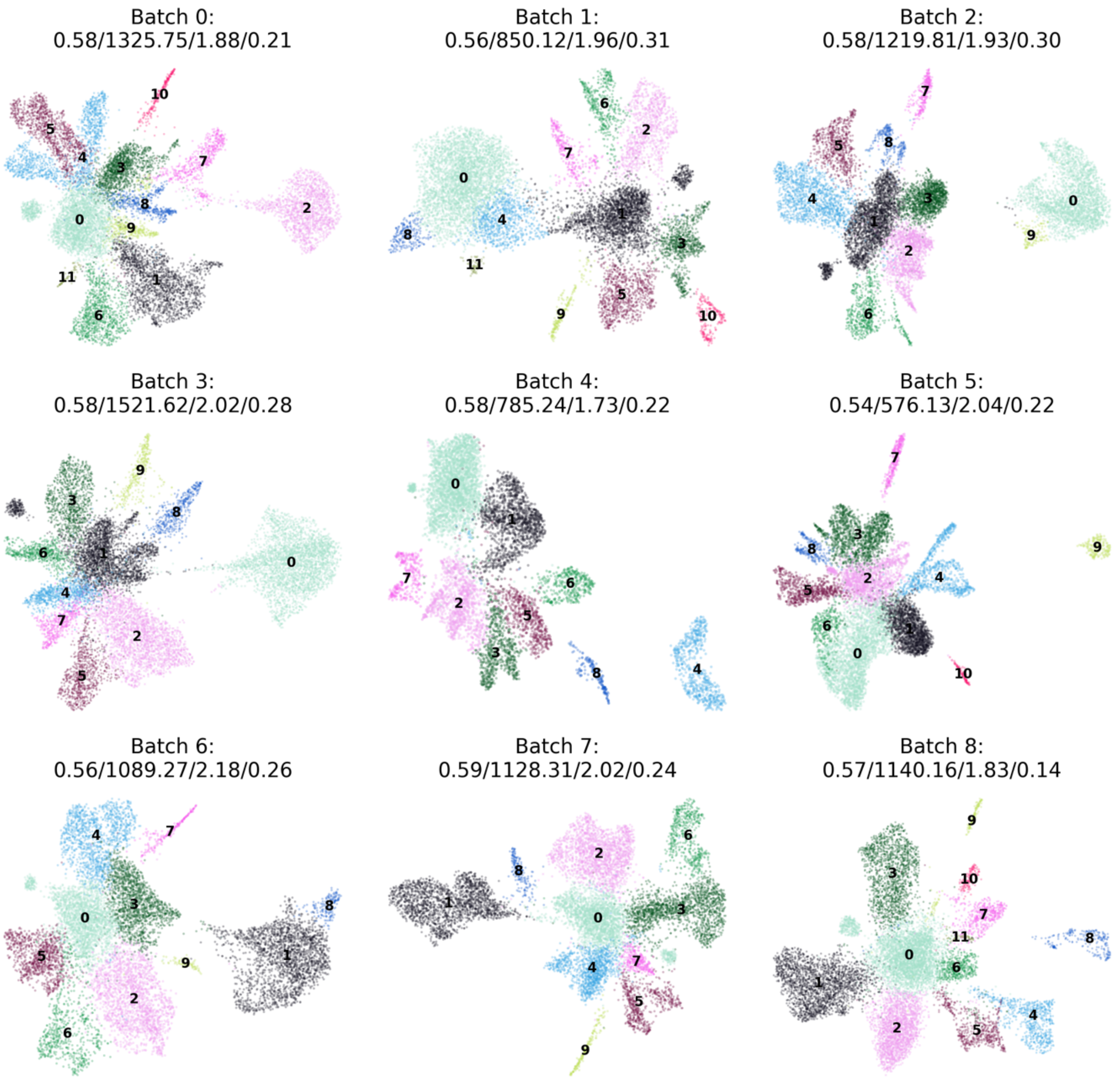
Batch 0:
0.58/1325.75/1.88/0.21
Batch 1:
0.56/850.12/1.96/0.31
Batch 2:
0.58/1219.81/1.93/0.30
Batch 3:
0.58/1521.62/2.02/0.28
Batch 4:
0.58/785.24/1.73/0.22
Batch 5:
0.54/576.13/2.04/0.22
Batch 6:
0.56/1089.27/2.18/0.26
Batch 7:
0.59/1128.31/2.02/0.24
Batch 8:
0.57/1140.16/1.83/0.14

B

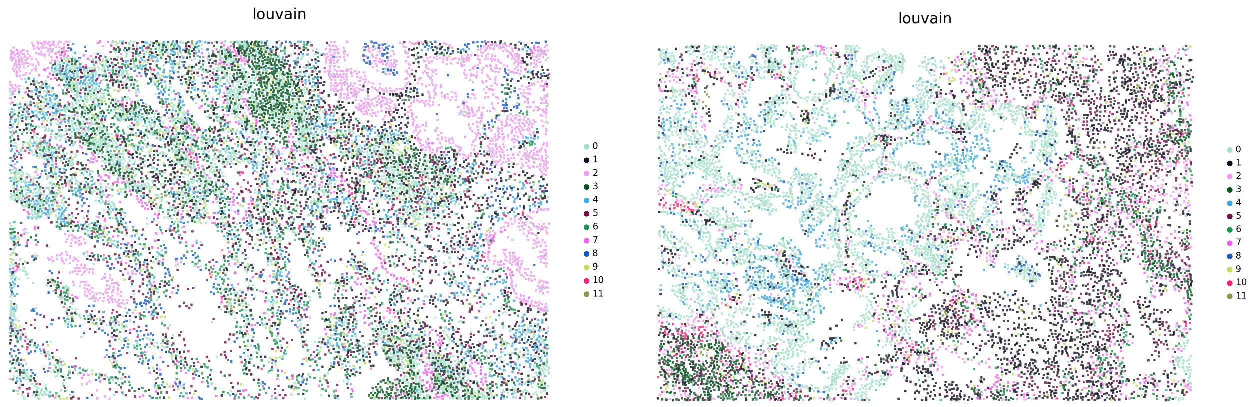
louvain
louvain

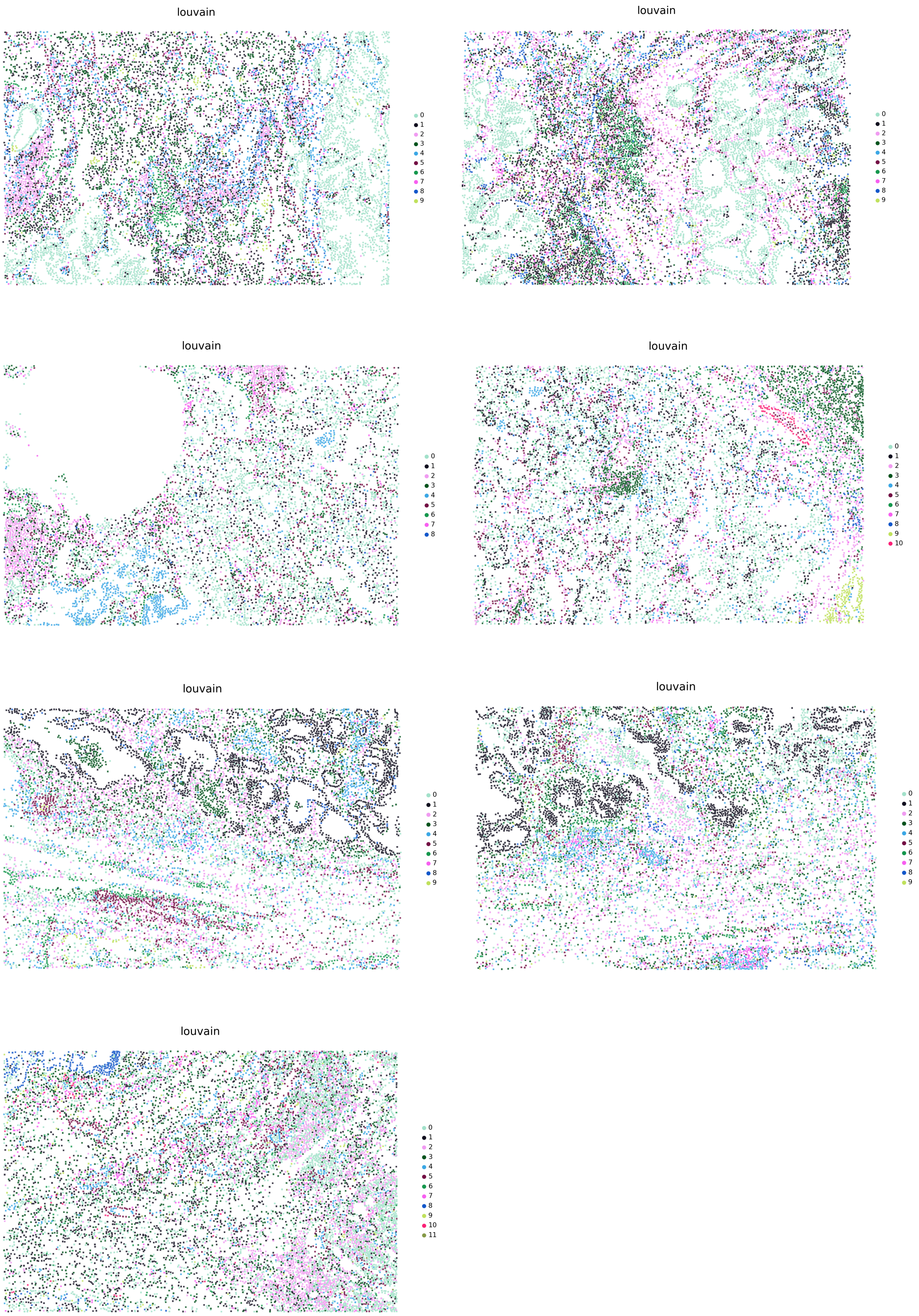
louvain
0
1
2
3
4
5
6
7
8
9
louvain
0
1
2
3
4
5
6
7
8
9
louvain
0
1
2
3
4
5
6
7
8
louvain
0
1
2
3
4
5
6
7
8
9
10
louvain
0
1
2
3
4
5
6
7
8
9
louvain
0
1
2
3
4
5
6
7
8
9
louvain
0
1
2
3
4
5
6
7
8
9
10
11

C

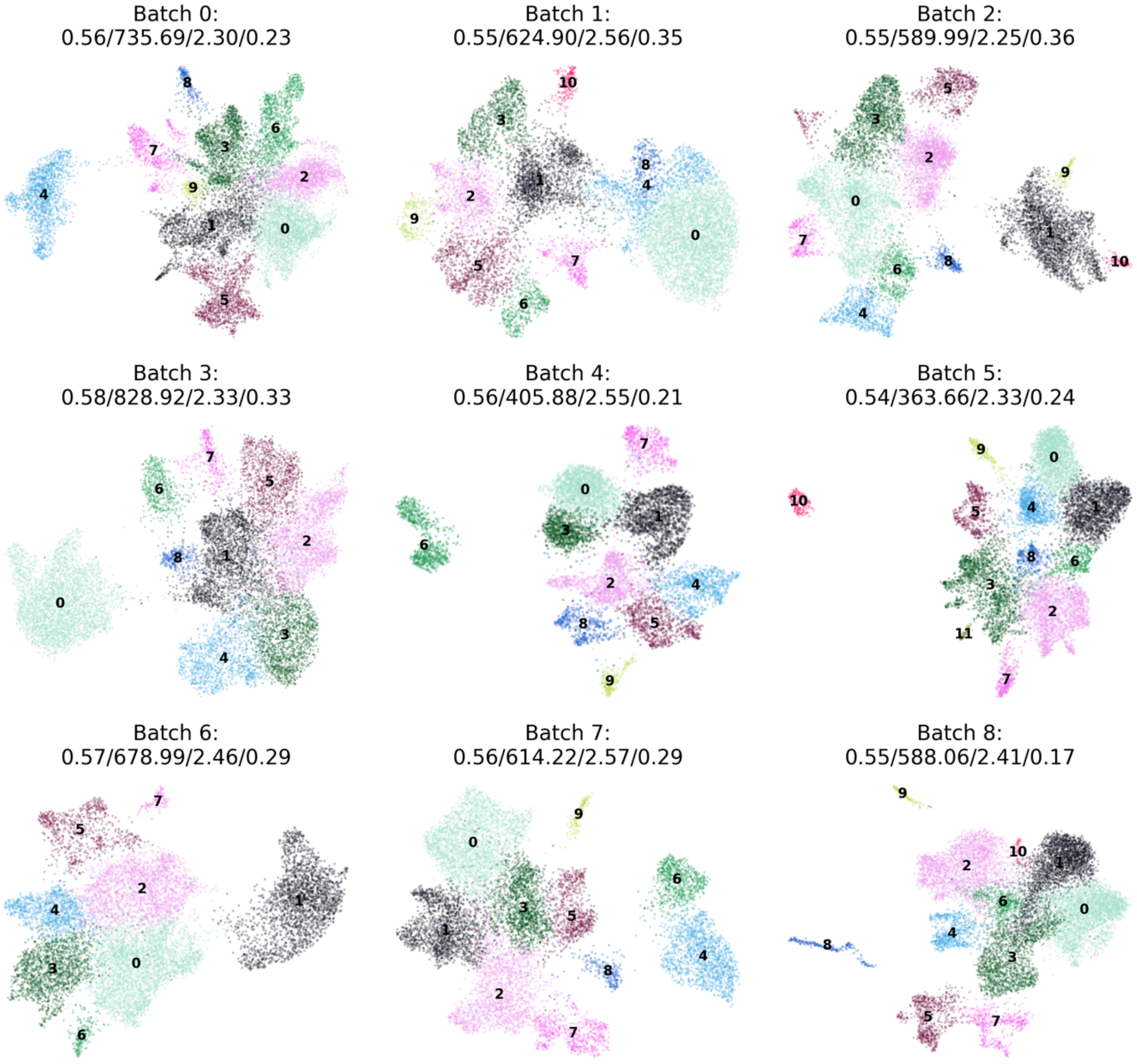
Batch 0:
0.56/735.69/2.30/0.23
Batch 1:
0.55/624.90/2.56/0.35
Batch 2:
0.55/589.99/2.25/0.36
Batch 3:
0.58/828.92/2.33/0.33
Batch 4:
0.56/405.88/2.55/0.21
Batch 5:
0.54/363.66/2.33/0.24
Batch 6:
0.57/678.99/2.46/0.29
Batch 7:
0.56/614.22/2.57/0.29
Batch 8:
0.55/588.06/2.41/0.17

D

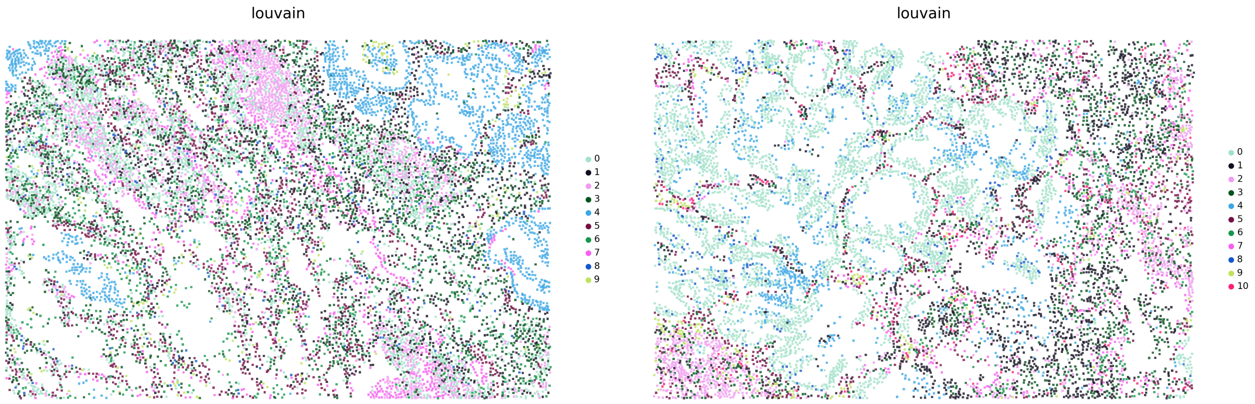
louvain
0 1 2 3 4 5 6 7 8 9
louvain
0 1 2 3 4 5 6 7 8 9 10

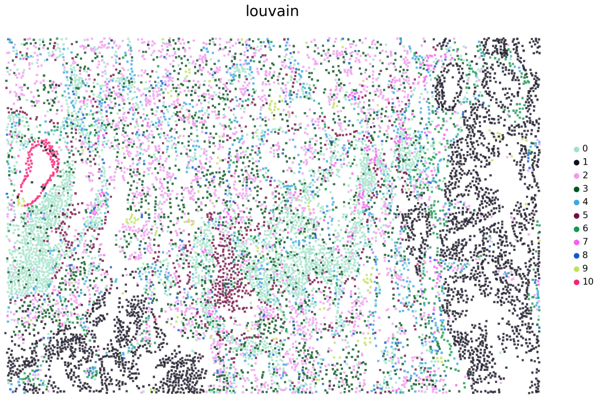
louvain
0
1
2
3
4
5
6
7
8
9
10

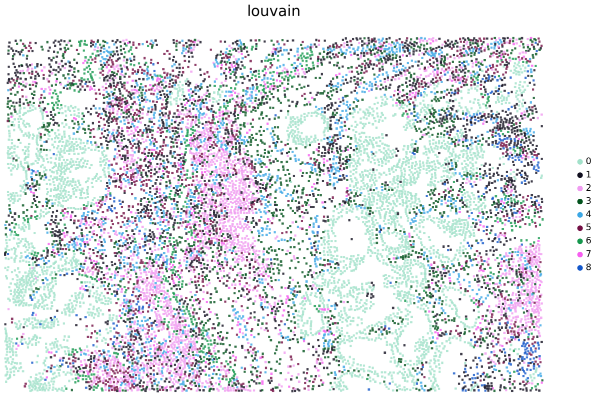
louvain
0
1
2
3
4
5
6
7
8

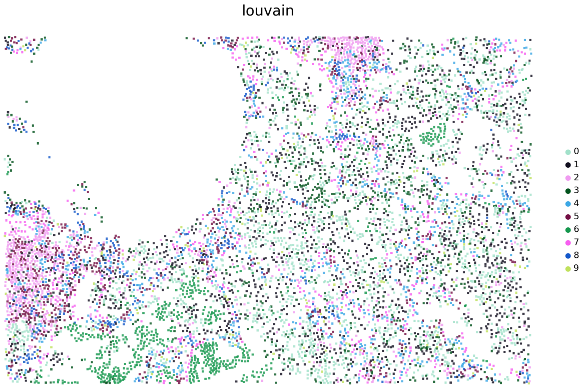
louvain
0
1
2
3
4
5
6
7
8
9

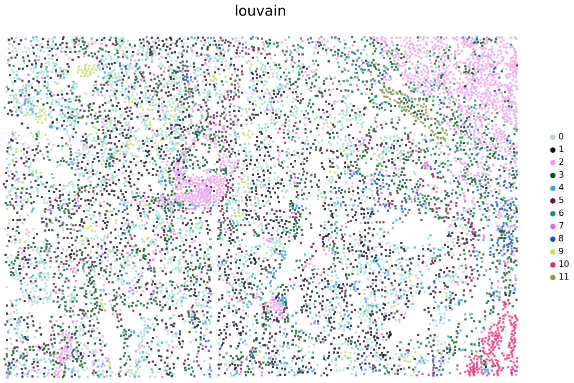
louvain
0
1
2
3
4
5
6
7
8
9
10
11

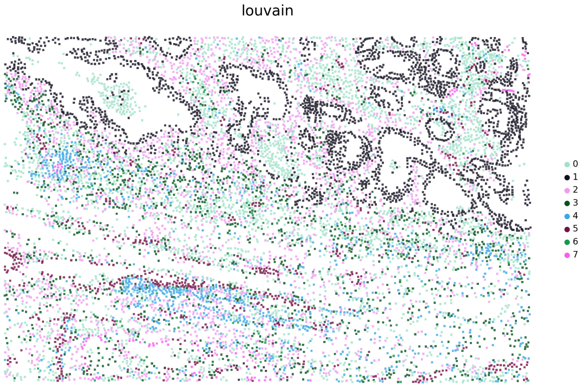
louvain
0
1
2
3
4
5
6
7

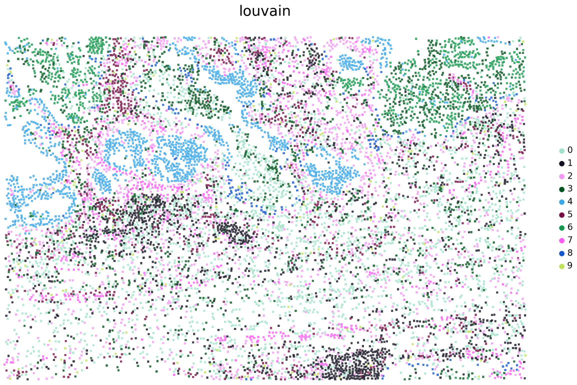
louvain
0
1
2
3
4
5
6
7
8
9

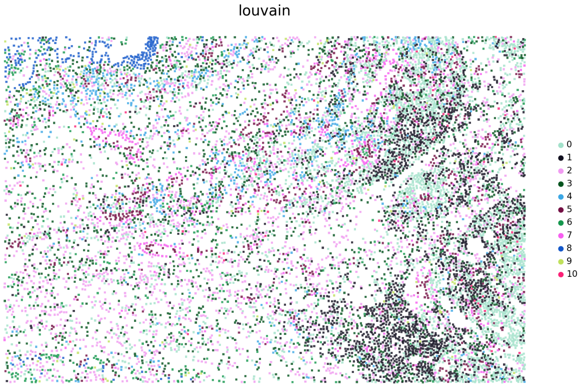
louvain
0
1
2
3
4
5
6
7
8
9
10

Supplementary Figure S10. Visualization of the SPICEiST clustering results compared to the GEX-based clustering in the CosMx SMI lung cancer dataset with 10x cell segmentation method at a resolution of 0.6.

UMAP plots and the spatial distribution of cells illustrate the cell-level embedding and spatial distribution derived from SPICEiST-based analysis (SPICEiST; A: UMAP, B: spatial distribution of cells) and cell-level gene expression-based clustering (GEX; C: UMAP, D: spatial distribution of cells), respectively. The tissue samples, obtained from patch numbers 0 to 8, are presented serially from left to right and top to bottom. The four numerical values at the top of the UMAP plot represent the ASW, CHI, DBI, and assortativity coefficient. Each dot in the plot corresponds to a cell, with color indicating the identity of the clusters.

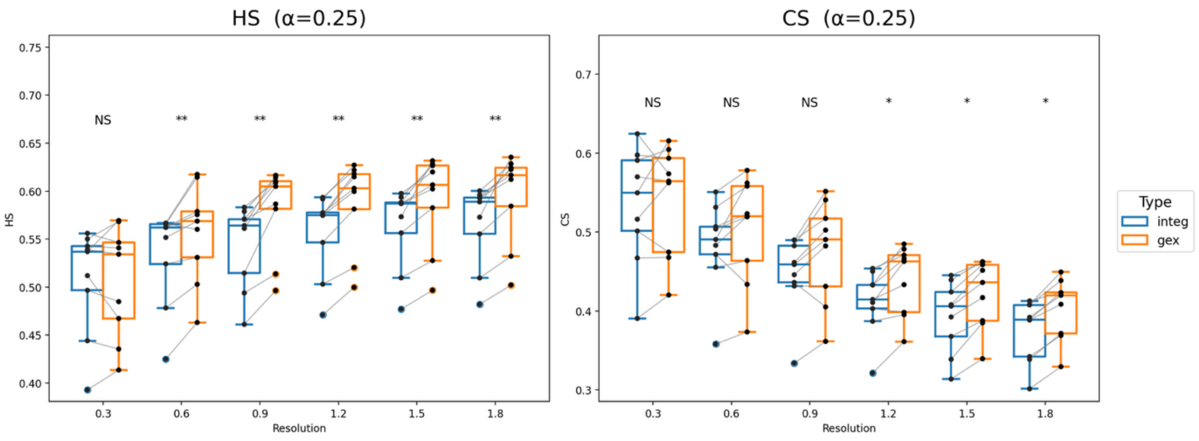

Supplementary Figure S11. Comparison of the homogeneity and completeness scores from SPICEiST and GEX in the CosMx SMI lung cancer dataset.

The boxplots compare homogeneity and completeness scores (HS and CS), across varying resolutions of cell clusters. The alpha weight for the loss was set to 0.25 during training. The stars above the boxplots show statistical comparisons (Wilcoxon signed-rank test) of GEX (annotated as "gex") with SPICEiST (annotated as "integ"). Multiple comparison correction was performed using the Benjamini-Hochberg method. NS: Not significant, *: $p \leq 0.05$, **: $p \leq 0.01$, ***: $p \leq 0.001$, ****: $p \leq 0.0001$.
